# Functional traits mediate the effect of land use on drivers of community stability within and across trophic levels

**DOI:** 10.1126/sciadv.adp6445

**Published:** 2025-01-24

**Authors:** Marta Gaia Sperandii, Manuele Bazzichetto, Lars Götzenberger, Marco Moretti, Rafael Achury, Nico Blüthgen, Markus Fischer, Norbert Hölzel, Valentin H. Klaus, Till Kleinebecker, Felix Neff, Daniel Prati, Ralph Bolliger, Sebastian Seibold, Nadja K. Simons, Michael Staab, Wolfgang W. Weisser, Francesco de Bello, Martin M. Gossner

**Affiliations:** ^1^ Centro de Investigaciones sobre Desertificación (CSIC-UV-GV), Valencia, Spain.; ^2^ Department of Botany and Zoology, Faculty of Science, Masaryk University, Brno, Czech Republic.; ^3^ Faculty of Environmental Sciences, Department of Spatial Sciences, Czech University of Life Sciences Prague, Kamýcka 129, Praha-Suchdol, Czech Republic.; ^4^ Department of Botany, Faculty of Science, University of South Bohemia, České Budějovice, Czech Republic.; ^5^ Czech Academy of Sciences, Institute of Botany, Třeboň, Czech Republic.; ^6^ Conservation Biology, Swiss Federal Research Institute WSL, Birmensdorf, Switzerland.; ^7^ Terrestrial Ecology Research Group, Department of Life Science Systems, School of Life Sciences, Technical University of Munich, Freising, Germany.; ^8^ Ecological Networks, Technical University Darmstadt, Schnittspahnstraße 3, Darmstadt, Germany.; ^9^ Institute of Plant Sciences, University of Bern, Bern, Switzerland.; ^10^ Institute of Landscape Ecology, University of Münster, Münster, Germany.; ^11^ Forage Production and Grassland Systems, Agroscope, Reckenholzstrasse 191, Zürich, Switzerland.; ^12^ Ruhr University Bochum, Institute of Geography, Bochum, Germany.; ^13^ Institute for Landscape Ecology and Resources Management (ILR), Justus Liebig University Giessen, Heinrich-Buff-Ring 26, Giessen, Germany.; ^14^ Centre for International Development and Environmental Research (ZEU), Justus Liebig University Giessen, Senckenbergstrasse 3, Giessen, Germany.; ^15^ Agroecology and Environment, Agroscope, Zurich, Switzerland.; ^16^ Forest Entomology, Swiss Federal Research Institute WSL, Birmensdorf, Switzerland.; ^17^ Forest Zoology, TUD Dresden University of Technology, Pienner Str. 7, Tharandt, Germany.; ^18^ Applied Biodiversity Science, Chair of Conservation Biology and Forest Ecology, Biocenter, University of Würzburg, Würzburg, Germany.; ^19^ Institute of Terrestrial Ecosystems, ETH Zurich, Zurich, Switzerland.

## Abstract

Understanding how land use affects temporal stability is crucial to preserve biodiversity and ecosystem functions. Yet, the mechanistic links between land-use intensity and stability-driving mechanisms remain unclear, with functional traits likely playing a key role. Using 13 years of data from 300 sites in Germany, we tested whether and how trait-based community features mediate the effect of land-use intensity on acknowledged stability drivers (compensatory dynamics, portfolio effect, and dominant species variability), within and across plant and arthropod communities. Trait-based plant features, especially the prevalence of acquisitive strategies along the leaf-economics spectrum, were the main land-use intensity mediators within and across taxonomic and trophic levels, consistently influencing dominant species variability. Functional diversity also mediated land-use intensity effects but played a lesser role. Our analysis discloses trait-based community features as key mediators of land-use effects on stability drivers, emphasizing the need to consider multi-trophic functional interactions to better understand complex ecosystem dynamics.

## Teaser

Functional traits, notably leaf-related trade-offs, shape land-use impacts on stability drivers within and across trophic levels.

## INTRODUCTION

Temporal community stability, i.e., its invariability in defined ecosystem properties such as biomass or abundance over time (see Box 1), is key to supporting multiple ecosystem functions and services over time (*1*, *2*), the provision of which is greatly challenged by the current biodiversity crisis (*2*–*4*). Currently, scientists agree that (i) compensatory dynamics, (ii) portfolio effects, and (iii) dominant species variability are key stability drivers (*1*, *5*–*8*) particularly controlling interannual constancy [used here as a basic estimator of stability, in line with previous research (*7*, *8*); see Box 1]. Compensatory dynamics occur when fluctuations of individual species compensate for each other, i.e., when year-to-year changes in the abundance of some species in a community are offset by changes in the abundance of other species. This mechanism, often measured by the degree of asynchrony (i.e., lack of temporal synchrony), positively affects community stability and is generally attributed to (i) discordant or time-lagged species responses to environmental (e.g., weather) fluctuations (*9*, *10*), (ii) demographic stochasticity (*11*), and (iii) competitive interactions (*12*, *13*). The “portfolio” or “averaging” effect (*14*, *15*) is the positive effect of species richness on stability, which is boosted under particular conditions including asynchronous species fluctuations, even abundances or due to overyielding and/or the so-called mean-variance scaling (*16*). The rationale behind is that more species increase the likelihood of different behaviors leading to environmental tolerance, which should result in an overall less fluctuating community. Last, dominant species variability (generally measured by a weighted average of species variability; see Box 1) should affect community stability in line with Grime’s “mass ratio hypothesis” (*17*), which states that the impact of a species in a community is proportional to its abundance. Therefore, species that are both locally abundant and have more stable populations are expected to stabilize the entire community (*18*).

Despite general consensus on the ecological mechanisms affecting stability, it is still unclear through which paths and to what extent land-use intensification, as a major driver of biodiversity loss (*19*), modulates the relative effects of these mechanisms (*20*). There is some recent evidence that land-use intensity (LUI) has a negative indirect impact on community stability through decreased community asynchrony and species diversity (*21*) as well as increased dominant species variability (*22*). At the same time, changes at one trophic level, such as primary producers, will also affect higher trophic levels (*23*, *24*). Consequently, higher trophic levels are expected to be affected by changes in LUI directly and indirectly through changes in community properties of lower trophic levels. Linking LUI to known drivers of community stability (i.e., compensatory dynamics, portfolio effect, and dominant species variability) within and across trophic levels, therefore, remains an urgent task of high relevance for both theoretical and applied ecological research.

Functional traits, i.e., morphological, physiological, or phenological features that determine the response of organisms to the environment and/or their effects on ecosystem properties (*25*), may be the missing piece of the jigsaw puzzle (*26*) that reveals the mechanistic links between LUI and drivers of community stability. LUI was shown to shape the functional trait structure of communities of different organisms (*19*, *27*, *28*). In addition, there is growing evidence of a stabilizing effect of key functional features, such as dominant species traits [as summarized by indices such as community-weighted mean (CWM); see Box 1] (*7*, *18*) or functional diversity, i.e., trait dissimilarity among species in communities (*10*, *29*, *30*). The dominance of species with more conservative strategies in a community [for plants often summarized within the leaf economics spectrum; (*31*)] should support temporal stability either through reduced dominant species variability or by directly acting on stability components, i.e., mean and SD of the targeted ecosystem function (*26*). High functional diversity in a community should lead to greater stability by increasing compensatory dynamics among species, i.e., community asynchrony. This is because a highly diversified set of strategies in a community should result in diverse responses to environmental perturbations, e.g., drought, flooding, storms, windstorms, and, more generally, interannual weather fluctuations (*26*). The positive effect of functional diversity on community stability could also be related to overyielding, where diversified strategies in a community arising through niche differentiation among species eventually lead to increased total abundance (*32*). At the same time, functional diversity (i.e., the temporal coexistence of different ecological strategies) could also affect dominant species variability, although very little is known on the direction of this effect.

Functional traits can also explain multi-trophic interactions (*33*) and cascading effects of land use on different types of organisms (*34*, *35*). Changes in the trait composition within a given trophic level in response to environmental drivers will likely alter traits that are linked to higher trophic levels [see the multi-trophic response-effect framework; (*36*)]. LUI is known to affect plant traits determining the response to resource availability (*19*), e.g., traits related to the leaf economics spectrum associated to growth rate and resource use (*31*). In turn, these plant traits could affect [in combination with direct effects of LUI; (*37*)] arthropod traits also involved in species responses to resource-acquisition and land-use pressure, such as morphometric, feeding, and dispersal traits, and, ultimately, influence the total abundance (and, in a temporal context, the stability) of arthropod communities.

Although many aspects of the stability jigsaw puzzle have been explored [e.g., the diversity-stability relationship (*8*, *12*, *14*, *15*), the influence of land use and management on stability (*6*, *10*, *21*), or the role of functional traits (*10*, *29*, *30*)], a comprehensive analysis that connects all these aspects is still largely missing. So far, the few analytical attempts made (*7*, *21*) mostly relied on short time-series or experimental data and/or lacked integrating critical parts of the puzzle [i.e., land use (*7*) or functional traits (*21*)]. Specifically, while the importance of functional traits for ecosystem stability has already been suggested [see (*26*) and the references therein], the few existing empirical studies have mainly focused on their direct effects on community stability (*38*–*40*), thereby overlooking the possibility that their contribution operates through widely recognized stability drivers, rather than directly influencing stability. Furthermore, the role of functional traits in multi-trophic interactions has not yet been tested in a temporal context, i.e., it is still not clear to what extent the functional features of a particular trophic group determine the community stability of higher trophic groups (e.g., plants–arthropod herbivores or arthropod herbivores–arthropod carnivores). Last, an open question remains as to whether the complex relationships among the ecological mechanisms underpinning stability, particularly the role of traits, can be generalized across habitats or whether observed patterns differ between, e.g., grasslands and forests (*21*).

**Fig. 1.**
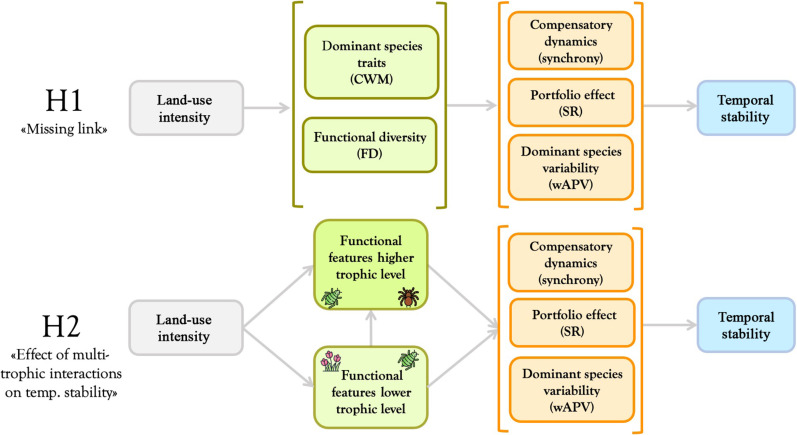
Graphical representation of the hypothesized causal flow. In H1, functional community features (represented as green boxes) mediate the effect of LUI on acknowledged stability drivers (orange boxes) within trophic levels. In H2, functional traits of the lower trophic level mediate the effects of LUI by influencing stability drivers both directly and indirectly through changes in the functional composition/diversity of higher trophic levels. Note that arthropod herbivores (represented in the figure by an aphid) can be considered both higher trophic level (with respect to plants) and lower trophic level with respect to arthropod carnivores (represented in the figure by a spider). For functional community features and acknowledged stability drivers, the relative metrics are specified in brackets. CWM, community-weighted mean; FD, functional diversity; SR, species richness; wAPV, weighted average population variability.

We used long-term (13 years) and large-scale (150 grassland and 150 forest sites) plant and arthropod data (three trophic levels) sampled along LUI gradients in three regions of Germany to test the following, not mutually exclusive, hypotheses (Fig. 1):

H1) “Missing link hypothesis”: The functional features of a community are key in linking LUI and direct stability drivers within individual taxonomic groups (i.e., plants and arthropods). Specifically, we predict that functional diversity will most strongly influence compensatory dynamics, whereas the dominant species’ traits will preferentially affect dominant species variability.

H2) “Effect of multi-trophic interactions on temporal stability hypothesis”: Direct stability drivers in higher trophic levels (e.g., arthropod herbivores and predators) respond to LUI both directly (i.e., via changes in their functional features; see H1) and indirectly, i.e., via changes in the functional features of lower trophic levels (e.g., plants and arthropod herbivores).

With this study, we aim to achieve a better understanding of the mechanisms underlying land-use effects on temporal community stability and its driving mechanisms, which is crucial for the development of more targeted strategies to preserve biodiversity and ecosystem functions.

Box 1 **Working definitions.**

**Table:** 

Stability	We refer to “temporal community stability” (hereafter, community stability or, simply, stability) as the ability of a community (in our case, all plants or arthropods recorded in a permanent sampling unit) to minimize the variability of its total abundance over time (*26*). Temporal stability is frequently measured with constancy (*7*, *8*), which is computed as the inverse of the coefficient of variation (CV; SD/mean). Here, stability is computed using summed vegetation cover for plants and total abundance for arthropods. The first represents the stability of vegetation structure, reflecting the balance and persistence of various plant species over time.
Dominant species variability and dominant species traits	Dominant species variability is here intended as the prevailing value of population variability in a community, frequently summarized by the weighted average population variability, corresponding to the community-weighted mean (CWM) computed on the CV of all species abundances in a community. Dominant species’ traits refer here to the traits of the “most abundant” species in a community. We summarize them using the first two axes of a principal components analysis (PCA) run on the CWM of ecologically relevant functional traits, reflecting the mass ratio hypothesis (also known as dominant species effect) (*17*). As CWMs weight species traits by their relative abundances, they serve as a proxy for the traits of the most abundant species.
Direct, indirect, total, and mediator effects	In a SEM, a direct effect is the effect of a variable (*x*) on another variable (*y*) to which it connects by means of a direct pathway. An indirect effect is the effect of *x* on *y* that is mediated by a third variable (*z*) that is directly connected to *x* and *y* and is computed by multiplying the effects of *x* on *y* and of *y* on *z*. A total effect is the sum of direct and indirect pathways connecting two variables and is computed by adding up the direct effect *x → y* and the indirect effect *x → z → y*. Mediators are all variables connecting a source (here, land-use intensity) to a target variable (here, direct stability drivers). The effect of a certain land-use mediator on a certain direct stability driver can be computed as the sum of all indirect paths linking land-use intensity to the target variable and going through that mediator.

## RESULTS

### Stability drivers within plant and arthropod communities

**Fig. 2.**
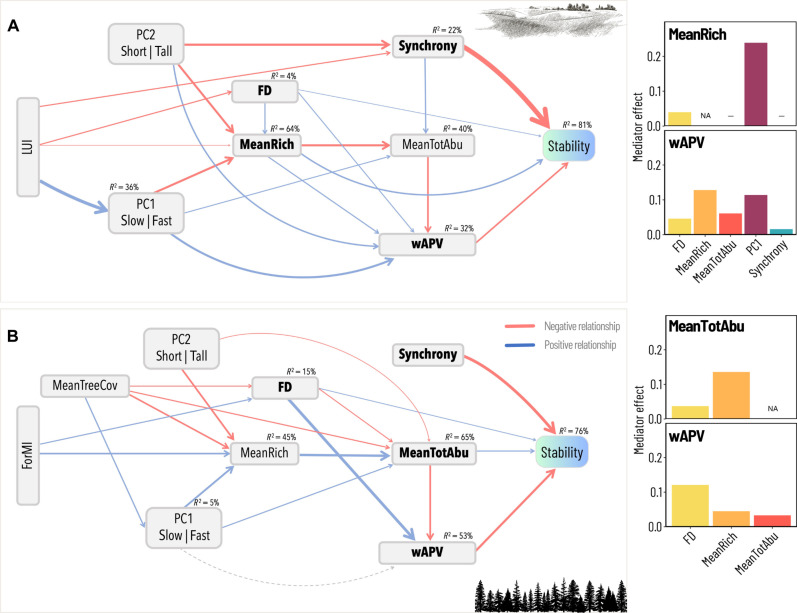
Functional features are key LUI mediators in plant communities. Structural equation models showing the interrelationships between LUI, functional features, acknowledged drivers of stability (compensatory dynamics, portfolio effect, and dominant species variability), mean total abundance, and stability (i.e., the inverse of the coefficient of variation) in grassland (**A**) and forest (**B**) plant communities. The thickness of the arrows is proportional to the slope of the relationship. Gray dashed lines indicate nonsignificant relationships. Both models showed a good fit (grasslands: Fisher’s *C* = 32.064, df = 26, *P* = 0.191, *n* = 150; and forests: Fisher’s *C* = 39.647, df = 36, *P* = 0.311, *n* = 144). *R*^2^ (coefficient of determination) values for constituent models are reported on top of each box and in section S4. For each SEM, the effect of individual LUI mediators (i.e., all variables connecting LUI and a stability driver; see Box 1) on direct stability drivers is shown as a bar plot on the right. A dash replacing a bar indicates that the variable on the *x* axis does not mediate the effect of LUI on a certain direct stability driver (represented on the *y* axis). NAs are for the mediating effect of a variable on itself. Bootstrapped mediator effects (i.e., the sum of all indirect paths operating through each mediator; see Box 1) are shown as absolute values for the purpose of comparing their strength irrespective of the direction. MeanTreeCov, mean tree cover; PC1 and PC2, first and second PCA axes used to define the dominant species traits; MeanRich, mean species richness; FD, functional diversity; MeanTotAbu, mean total abundance; wAPV, weighted average population variability. Top right and bottom right drawings: @vector-trend via canva.com.

**Fig. 3.**
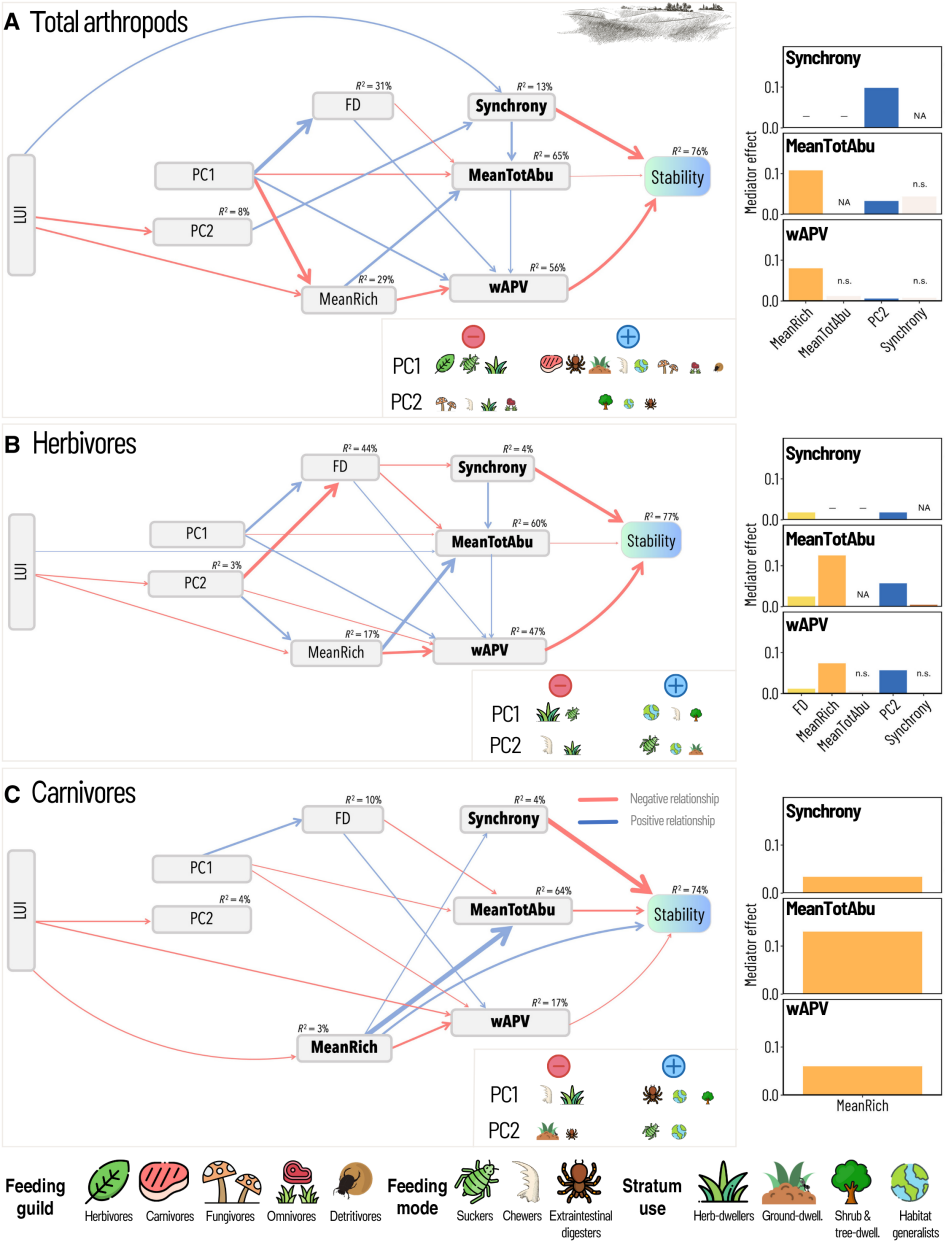
In arthropods, the mediating effects of functional features are less pronounced than in plants. Structural equation models showing the interrelationships between LUI, functional features, acknowledged drivers of stability (compensatory dynamics, portfolio effect, and dominant species variability), mean total abundance, and stability (inverse of the coefficient of variation) in grasslands for total arthropod (**A**), herbivore (**B**), and carnivore (**C**) communities. The thickness of the arrows reflects the slope of the relationship. All models showed a good fit (arthropods: Fisher’s *C* = 39.344, df = 34, *P* = 0.243, *n* = 150; herbivores: Fisher’s *C* = 40.074, df = 30, *P* = 0.103, *n* = 150; and carnivores: Fisher’s *C* = 50.672, df = 38, *P* = 0.082, *n* = 149). *R*^2^ values are reported above each box and in section S4. For each SEM, the effect of individual LUI mediators (variables connecting LUI and a direct stability driver; see Box 1) on direct stability drivers is shown as a bar plot on the right. Bootstrapped mediator effects (sum of all indirect paths operating through each mediator; see Box 1) are shown as absolute values to compare their strength irrespective of the direction. Transparent bars labeled “n.s.” indicate nonsignificant mediator effects. A dash replacing a bar indicates that the variable on the *x* axis does not mediate the effect of LUI on a certain stability driver (represented on the *y* axis). NAs are for the mediating effect of a variable on itself. PC1 and PC2, first and second PCA axes used to define the dominant species traits; MeanRich, mean species richness; FD, functional diversity; MeanTotAbu, mean total abundance; wAPV, weighted average population variability. Symbols in insets are scaled according to the strength of correlation between PC axes and CWM values of arthropod traits (see section S2C for interpretation of correlation matrices and axes). Symbols: “Flaticon.com.” Top right drawing: @vector-trend via canva.com.

Community synchrony and dominant species variability (summarized by weighted average population variability, hereafter wAPV) were generally the most frequent factors directly influencing temporal stability, followed by mean total abundance, species richness, and functional diversity (Figs. 2 and 3 and table S1). Concerning their total effect (i.e., the sum of direct and indirect effects; table S2), increased community synchrony and wAPV resulted in decreased temporal stability in both plant and arthropod communities. Mean total abundance was associated with increased plant and decreased arthropod stability. Overall, the total effect of species richness on stability was positive and particularly strong in grassland plants and forest arthropods.

### Effect of LUI on temporal stability and stability drivers

LUI had an indirect impact on community stability, varying in strength across the different groups (table S2 and Figs. 2 and 3). Significant negative effects were observed for total arthropods and herbivores in grasslands and for carnivores in forests (table S2).

Furthermore, the total effect of LUI on the different stability drivers varied in strength and direction depending on the variable and habitat considered (table S3). When significant, LUI decreased synchrony (plants, herbivores, and carnivores in grasslands) and species richness (plants and carnivores in grasslands) while increasing wAPV (total arthropods in grasslands and herbivores in both habitats). Its effect on mean total abundance was habitat and group dependent (positive for plant and carnivore communities in forests and negative for carnivores in grasslands).

### Mediating role of trait-based community features within single taxonomic and trophic groups (H1)

Within each taxonomic group, functional features, i.e., dominant species traits [summarized by the first two axes of a principal components analysis (PCA) run on the plot-specific CWMs] and functional diversity, were significant and most important mediators of the effect of LUI on direct stability drivers, especially for plants (Figs. 2 and 3; results for forest arthropods illustrated in fig. S11). In plant communities (Fig. 2), the role of the dominant species traits was evident only in grasslands, where it significantly mediated the effects of land use on species richness and wAPV, being the principal mediator for the species richness and the second most important mediator (after species richness) for wAPV. Specifically, increasing LUI resulted in the prevalence of acquisitive strategies (summarized by the first PCA axis; see sections S2B and S2C), which was associated with lower species richness and higher wAPV. Conversely, the mediating role of functional diversity was variable but visible in both habitats. In grasslands (Fig. 2A), functional diversity mediated the influence of LUI on species richness and wAPV. In forests (Fig. 2B), functional diversity mediated the effect of LUI on mean total abundance and wAPV. However, it was the principal mediator only for wAPV, with a strong, positive effect on the latter.

In arthropods, both functional features (i.e., dominant species traits and functional diversity) mediated the influence of LUI on stability drivers, but the mediating effects were weaker compared to those in plants, and showed marked differences depending on trophic group and habitat (Fig. 3; results for forests illustrated in fig. S11). For total arthropods in grasslands (Fig. 3A), higher LUI resulted in lower proportion of shrub/tree dwellers (summarized by the second PCA axis), which, in turn, was positively associated with all direct stability drivers (via synchrony), ultimately resulting in decreased community stability. In forests (fig. S11), all functional components were involved in the causal chain connecting LUI and stability drivers, yet the mediating action of functional diversity was not significant. The first PCA axis was the most important mediator for species richness and mean total abundance, whereas the second PCA axis was the key mediator for synchrony and wAPV. Here, on the one hand, higher LUI reduced the abundance of sucking arthropods, herbivores, and shrub/tree dwellers (PC1 in fig. S11). Their reduced abundance (corresponding to high values of PC1), in turn, decreased synchrony (via increased functional diversity) while increasing mean richness and mean total abundance, with an ultimate positive effect on community stability. On the other hand, higher LUI resulted in fewer herb dwellers and habitat generalists (PC2), which decreased wAPV but increased synchrony (both via reduced functional diversity), with almost no overall effect on stability. As yearly time series for forest arthropods were only available for 30 plots, bootstrapped effects were calculated, but results should be interpreted cautiously. When analyzing individual trophic groups, a weak mediating role of the functional features was only visible in herbivore communities (Fig. 3B). In carnivore communities, the paths linking LUI to functional features were excluded because of model selection, and species richness was the only mediator of the effect of land use on direct stability drivers (Fig. 3C).

### Mediating role of trait-based community features across taxonomic groups and trophic levels (H2)

**Fig. 4.**
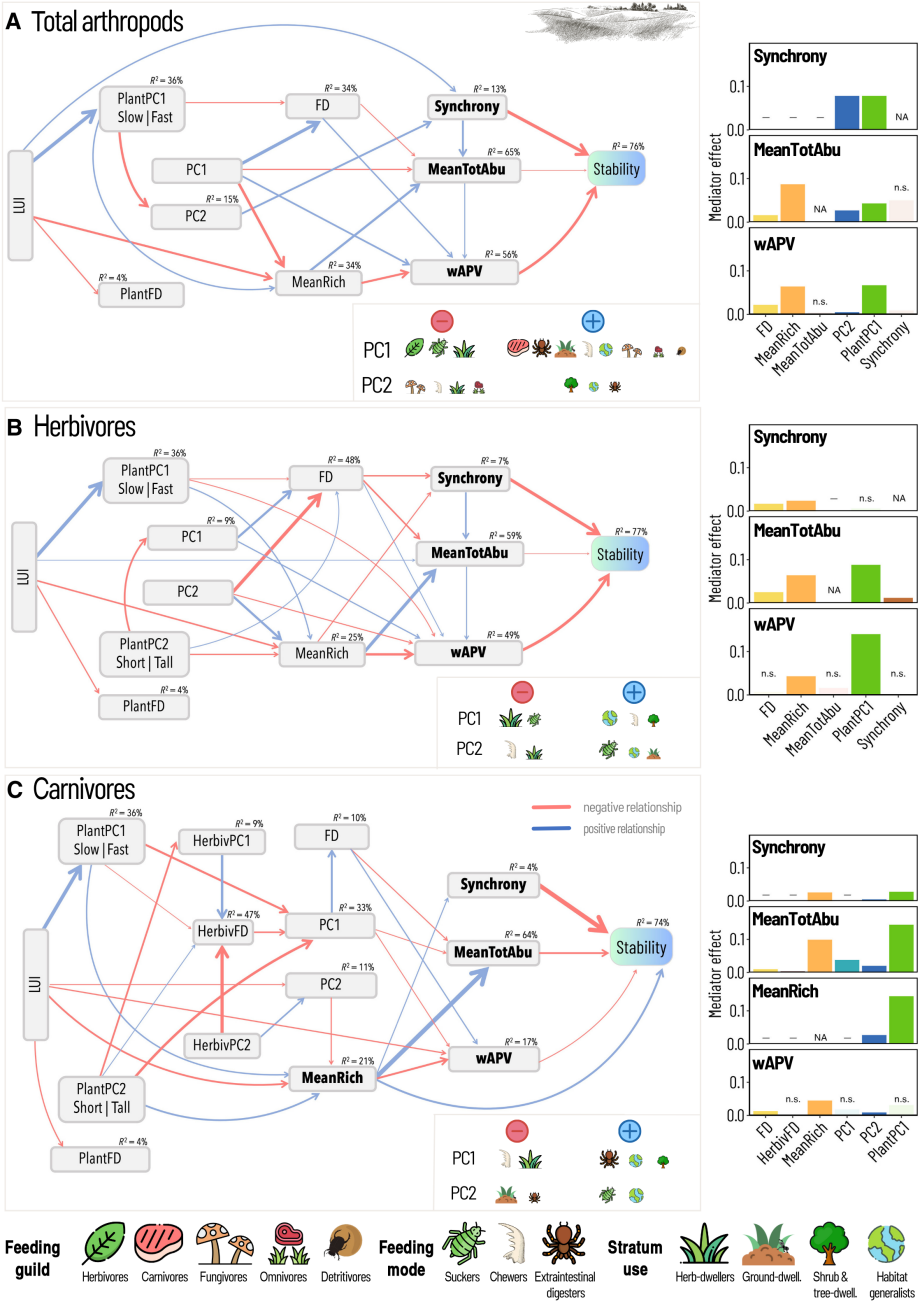
Leaf economics spectrum trade-offs mediate LUI effects across trophic levels. Structural equation models showing the interrelationships between LUI, functional features, acknowledged drivers of stability (compensatory dynamics, portfolio effect, and dominant species variability), mean total abundance, and stability (inverse of the coefficient of variation) in grasslands for total arthropod (**A**), herbivore (**B**), and carnivore (**C**) communities. The thickness of the arrows reflects the slope of the relationship. All models showed a good fit (arthropods: Fisher’s *C* = 54.344, df = 62, *P* = 0.745, *n* = 150; herbivores: Fisher’s *C* = 78.18, df = 68, *P* = 0.187, *n* = 150; and carnivores: Fisher’s *C* = 141.569, df = 140, *P* = 0.447, *n* = 149). *R*^2^ values are reported above each box and in section S5. For each SEM, the effect of individual LUI mediators (variables connecting LUI and a direct stability driver; see Box 1) on direct stability drivers is shown as a bar plot on the right. Bootstrapped mediator effects (sum of all indirect paths operating through each mediator; see Box 1) are shown as absolute values to compare their strength irrespective of the direction. Transparent bars labeled “n.s.” indicate nonsignificant mediator effects. A dash replacing a bar indicates that the variable on the *x* axis does not mediate the effect of LUI on a certain stability driver (represented on the *y* axis). NAs are for the mediating effect of a variable on itself. PC1 and PC2, first and second PCA axes used to define the dominant species traits; MeanRich, mean species richness; FD, functional diversity; MeanTotAbu, mean total abundance; wAPV, weighted average population variability. Symbols in insets are scaled according to the strength of correlation between PC axes and CWM values of arthropod traits (see section S2C for interpretation of correlation matrices and axes). Symbols: “Flaticon.com.” Top right drawing: @vector-trend via canva.com.

The role of functional traits in mediating the effect of LUI on direct stability drivers was evident across both taxonomic groups and trophic levels. Structural equation models (SEMs) including trophic functional links showed that, in grasslands, the dominant species traits in plants significantly mediated the effect of LUI on all direct drivers of total arthropod stability, being actually the main mediator for wAPV and (together with the dominant species traits in arthropods, PC2) synchrony (Fig. 4). In most cases, this mediation occurred through arthropod functional features. Higher LUI promoted plant communities dominated by species featuring acquisitive strategies [high specific leaf area (SLA), low leaf dry matter content (LDMC), and high content of leaf nutrients]. On the one hand, such prevalence of acquisitive strategies in plants was negatively associated with the proportion of shrub/tree-dwelling arthropods (PC2), resulting in decreased arthropod synchrony and, via the latter, in decreased mean total abundance and wAPV (Fig. 4A). On the other hand, prevalence of acquisitive strategies reduced arthropod functional diversity, eventually resulting in increased mean total abundance and wAPV (indirectly via mean total abundance) and decreased wAPV. Through these two paths, the prevalence of acquisitive strategies in plants ultimately led to increased arthropod stability (total effect, 0.115; 95% confidence interval [0.074, 0.164]). When considering individual trophic groups (Fig. 4, B and C), the dominant species traits of plant communities remained the most important mediator for almost all direct stability drivers in herbivorous and carnivorous arthropod communities, even surpassing their own (arthropod) functional features. The only exceptions were synchrony (for herbivores) and wAPV (for carnivores), for which the main land-use mediator was mean richness. Again, the path linking plant functional features to direct stability drivers often went through arthropod functional features. In herbivore communities (Fig. 4B), the land-use–driven prevalence of acquisitive strategies in plants was associated with a reduced functional diversity in herbivores, ultimately resulting in increased mean total abundance, decreased wAPV, and almost no net effect on synchrony. In carnivore communities (Fig. 4C), the prevalence of acquisitive strategies in plants translated into a reduced dominance by extraintestinal digesters and habitat generalists (both directly and weakly via a reduction in herbivore functional diversity), with an ultimate negative effect on (carnivore) functional diversity. Eventually, this resulted in enhanced synchrony, species richness, and mean total abundance.

In total arthropods, as well as in herbivore and carnivore communities, species richness was also a frequent mediator of the effects of LUI on direct stability drivers. However, the mediating effect of the dominant species traits along the leaf-economics spectrum in plant communities was stronger than that of species richness for most direct stability drivers (Fig. 4 and section S7).

## DISCUSSION

The long-standing question of what drives temporal stability in ecological communities has sparked a lively and controversial debate (*12*, *15*). By analyzing well-replicated plant and arthropod time-series data, we aimed to deepen our understanding of how community stability and its key driving mechanisms are shaped by varying land-use intensities in real-world ecosystems. Specifically, we tested the hypothesis that functional traits mediate the effect of LUI by acting on widely recognized stability-driving mechanisms and that this effect occurs both within (H1) and across (H2) taxonomic and trophic levels. While providing further evidence of an indirect, largely negative effect of LUI on community stability (*21*), our results confirm compensatory dynamics and dominant species variability as key stability-driving mechanisms. In line with our hypotheses, our results highlight community functional features, namely, dominant species traits and functional diversity, as key mediators of LUI effects on stability drivers, rather than direct determinants of stability. However, the strength of this mediating effect strongly varied across taxonomic/trophic groups and habitats, with plant traits emerging as the main mediators of LUI effects on community stability drivers. The mediating role of dominant species traits appeared somehow stronger than that of functional diversity. While we confirmed that dominant species traits mainly contribute to stability via dominant species variability, particularly for plants in grassland, the link between functional diversity and compensatory dynamics was less consistent and primarily observed for plants in forest. This suggests that LUI affects different functional components in these two habitats (*41*), potentially due to intrinsic ecological differences or divergent management practices. Consistent with our second hypothesis (H2), the mediating role of functional traits extended across trophic levels, with leaf economics spectrum trade-offs emerging as important mediators of LUI effects on stability drivers for both total arthropods and herbivores in grasslands. Overall, our results revealed a composite picture characterized by complex interrelations that require detailed discussion.

So far, compensatory dynamics (i.e., low synchrony), the portfolio effect (i.e., increased species richness), and dominant species variability (i.e., high community-weighted average of species variability) were suggested as key drivers directly influencing stability (*1*, *5*–*8*). In line with previous research (*8*, *21*, *30*), we found that compensatory dynamics and dominant species variability were the most important mechanisms controlling community stability of both plants and arthropods directly: compensatory dynamics enhancing stability, while dominant species variability reducing it. However, indirectly, increased LUI has also significantly reduced community stability via its effect on stability drivers. Although no significant effect was found for plants [consistently with (*21*), and possibly due to different management practices having contrasting effects on stability (*10*, *42*)], LUI decreased the stability of total arthropods in grassland communities. This was also true for herbivores in grasslands and carnivores in forests, suggesting that the detrimental effect of LUI on stability might affect key ecosystem functions, as suggested by studies analyzing herbivory and predation in response to LUI (*43*–*45*). The fact that, in grasslands, higher LUI resulted not only in poorer plant communities but also in less stable communities of herbivorous arthropods supports findings from biodiversity experiments (*23*, *46*) and provides evidence that increased LUI has cascading effects through trophic levels in natural communities.

Functional features had only few direct effects on community stability (functional diversity in grassland and forest plants and dominant species traits in forest herbivores), which supports recent findings from avian (*47*) and plant (*48*) communities. In contrast, previous research (*7*) found that dominance by conservative plant species [so-called “slow” species according to the leaf economics spectrum; (*49*)] was directly and positively linked to stability. In our study, dominance of conservative species was positively related to stability in plant communities, but this effect was indirect as it was mediated by the portfolio effect (i.e., species richness), mean total abundance, and dominant species variability (wAPV). While confirming that conservative strategies ultimately promote temporal stability in plant communities (*18*, *39*), our findings highlight the need to better elucidate the nature of this relationship (i.e., direct or indirect).

Including functional traits improved the explanation of the causal paths linking LUI and direct stability-driving mechanisms within taxonomic/trophic groups, but this mediating effect was mainly visible in plants (Figs. 2 and 3). Our results show that the functional features of a community, namely, dominant species traits and functional diversity, are mostly significant and often the principal mediators of LUI effects on direct drivers of plant community stability (see Fig. 2). This was not so evident in arthropod communities, except for total arthropods in forests. Nevertheless, the mediating effect of community functional features was not exclusive, i.e., other variables, especially mean species richness (summarizing the portfolio effect), were also important land-use mediators both in plant and arthropod communities, and their individual role was not completely unambiguous.

Dominant species traits acted as important mediators in grassland plant communities and in forest total arthropods. This is in line with findings that land-use intensification substantially alters the functional composition of biological communities (*19*, *27*). It further suggests that functional composition can strongly affect stability-driving mechanisms (*30*) and, thus, ultimately affect ecosystem stability. In line with our initial hypothesis, the dominant species traits (represented by variation along the leaf-economics spectrum) proved to be a key land-use mediator for dominant species variability in grassland plant communities. Here, increasing LUI was associated with communities dominated by fast-growing plant species, characterized by acquisitive strategies along the leaf economics spectrum (*28*). This, in turn, increased dominant species variability [probably because “fast” species rapidly respond to environmental changes; (*18*, *26*, *49*, *50*)], which ultimately resulted in reduced community stability. This finding provides additional evidence that the dominant strategy along the leaf economics spectrum in a community is a fair predictor of its temporal stability (*7*, *18*, *39*). Also in forest total arthropods, the dominant species traits (summarizing variation in prevalent feeding guild, mode, and habitat use) were a key land-use mediator for dominant species variability (fig. S11). In this case, LUI had a contrasting effect on dominant species variability: positive via a reduction in the abundance of sucking arthropods, herbivores, and shrub/tree dwellers (PC1), and negative via a decrease in herb dwellers and habitat generalists (PC2). Although the net effect of LUI on dominant species variability was close to zero, its impact on all other stability drivers was still mediated by the dominant species traits (through the two principal component axes). This suggests that land use can substantially affect important stability mechanisms by altering arthropod community composition in terms of feeding preferences, feeding habits, and habitat suitability. For most arthropod groups (total arthropods in both habitats, and herbivores and carnivores in grasslands), PC2 correlated positively with the CWM of body size and/or negatively with the CWM of dispersal ability (section S8). Given the consistent negative relationship between LUI and PC2 in those groups, this seems to suggest that LUI promotes communities dominated by smaller species and with high dispersal ability, which have been recently pointed out as strategies characterizing fast arthropod communities (*51*).

The mediating role of functional diversity appeared somehow weaker than that of the dominant species traits. While functional diversity often played a significant mediating role (especially in plant communities), it was the principal land-use mediator only for dominant species variability in forest plant communities and for compensatory dynamics (summarized by synchrony) in grassland herbivorous arthropods where it shared first place with dominant species traits. Besides, functional diversity was unexpectedly linked to dominant species variability, which overall contradicts our initial hypothesis of a consistent link with compensatory dynamics. Functional diversity was found to be significantly positively associated with dominant species variability across habitats and taxonomic groups, with a few exceptions (forest herbivores and carnivores). If we assume the diversity of ecological strategies in a community to be a proxy for interspecific competition [see the limiting similarity theory; (*52*)], then this relationship might support findings that competitive interactions increase population variability (*12*, *53*) while also pointing to an ultimate, negative effect on community stability.

Our results highlight that the functional features of lower trophic levels (e.g., plants and arthropod herbivores) not only affect their own direct stability drivers but also mediate the influence of LUI on the stability-driving mechanisms of higher trophic levels (e.g., arthropod herbivores and predators). This mediating role of functional traits across trophic levels was particularly evident in grasslands (Fig. 4). Here, the inclusion of trophic functional linkages revealed the leaf economics spectrum as the actual link between LUI and the dominant species traits (summarized by PC2) in total arthropods (Fig. 4A) and between LUI and functional diversity in herbivores (Fig. 4B). Although this does not suggest a direct effect of plant functional traits on ecosystem properties related to higher trophic levels, consistent with recent findings (*54*), it still demonstrates that plant traits, by influencing traits at higher trophic levels, play a crucial role in the causal chain linking LUI and community stability through their effect on its drivers. Further, it highlights that considering multi-trophic interactions while also accounting for the environmental context is crucial to increase our understanding of complex ecosystem dynamics (*55*, *56*).

The dominance of acquisitive strategies in plant communities, driven by increasing LUI, resulted in a shift in the dominant species traits of predators (i.e., it reduced the proportion of suckers and habitat generalists), mostly directly but partly also via reduced herbivore functional diversity (i.e., enhanced similarity in terms of average body size, variability in body size, and dispersal ability). Our findings provide additional evidence for tight functional linkages between plants and arthropods (*23*, *24*). They also show that environmental and disturbance gradients imposed by land use can cascade along the trophic chain and alter ecosystem processes (*33*, *34*, *57*). In arthropod communities in grasslands, the dominant species traits of plants were the strongest land-use mediator on almost all stability-driving mechanisms, suggesting that across-level functional linkages may outweigh the mediating effect of within-level functional features. On the one hand, this confirms that primary producers play a vital role in structuring multi-trophic interactions (*34*, *57*) and may indicate “bottom-up control” mechanisms (*58*). On the other hand, it suggests that, besides taxonomic features [e.g., species richness; (*59*)], plant functional features also induce shifts in the functional composition of arthropod communities. Exceptions to the observed strong mediating role of plant functional features were mean total abundance for total arthropods, compensatory dynamics (summarized by synchrony) for herbivores, and dominant species variability for carnivores. In these cases, the most important land-use mediator was species richness, which requires further investigations to clarify the possible role of the portfolio effect as a stability-mediating mechanism rather than a direct driver. For instance, fast-growing, highly productive plant communities, resulting from increased LUI (see Fig. 1), were positively associated with total arthropod richness and especially with herbivore richness (Fig. 3). This, in turn, had positive total effects on their stability, which may potentially and indirectly suggest that mechanisms related to overyielding in plants translate into higher richness and stability in arthropods (*46*).

In conclusion, our study provides a comprehensive picture of the complex and multifaceted biotic mechanisms driving temporal stability. Our analysis provides first evidence that functional traits related to the leaf economics spectrum in plants and to morphometrics as well as ecological preference in arthropods mediate the effect of land use on direct stability drivers. This interplay may ultimately affect stability in plant and arthropod communities, although the role of individual trait-based features appears to be context dependent. Moreover, our results suggest that the mediating role of the dominant species traits in plants can propagate through trophic levels and even override that of arthropod functional features. Therefore, our study provides important contributions to a better understanding of the mechanistic interplay between land-use intensification, functional traits, and stability drivers, which is essential for developing strategies for maintaining biodiversity-related ecosystem functions and services.

Nature is inherently complex, and understanding ecological patterns necessitates engaging with the complexity, which can be a challenging task, particularly in observational studies that span multiple habitats and trophic levels. In our study, the observed relationships unveil a complex interplay, which might not be easily generalized to other ecosystems or land-use modes and might vary depending on the available set of functional traits used. Moreover, the documented relationships in forest arthropod communities would especially benefit from repeating the analysis with a larger sample size in the future. Yet, our results open up avenues for deepening our understanding on the drivers of temporal stability and disentangling the specific role of trait-based features in natural systems subject to land-use intensification. In this regard, future research should focus on (i) closing existing gaps in trait databases to investigate the role of additional informative functional traits, such as belowground plant traits and quantitative arthropod effect traits, which are critical for advancing our understanding of ecosystem dynamics (*60*); and (ii) the potential role of altered plant-herbivore interactions (*59*) in linking environmental perturbations and ecosystem stability given the growing evidence of their importance to many ecosystem processes and functions.

## METHODS

### Study area

This study was conducted within the framework of the Biodiversity Exploratories (BE) project (*41*). The BE is a long-term and large-scale project covering three regions in Germany: the UNESCO biosphere reserve Schwäbische Alb [South-west Germany; 48°340 to 48°530 N; 9°180 to 9°600 E; 460 to 860 m above sea level (asl)], the National Park Hainich-Dün and surrounding areas (Central Germany; 50°940 to 51°380 N; 10°170 to 10°780E; 285 to 550 m asl), and the UNESCO biosphere reserve Schorfheide-Chorin (Northeast Germany; 52°470 to 53°130 N; 13°230 to 14°090E; 3 to 140 m asl). While the three regions differ in climate, geology, topography, and dominant soil types, they cover comparable gradients of LUI representative for grasslands and forests in most of temperate Europe. Within each region, 50 grassland and 50 forest sites (total 300) were selected to cover the whole range of land-use intensities (from unmanaged forests and low-intensively used grasslands to intensively used forests and grasslands) and to minimize confounding effects of spatial position and soil properties [so called experimental plots; (*61*)]. Each experimental plot (1 ha in forests and 0.25 ha in grasslands) was established within a larger management unit.

### Land use

In grasslands, land use was assessed yearly for each plot through standardized questionnaires sent to landowners (*61*) considering three components: fertilization, mowing, and grazing. For each plot, we used the mean value of the LUI index (*61*) for the years 2006 to 2019, standardized across the three regions. LUI values were extracted using the LUI calculation tool (*62*) implemented in the Biodiversity Exploratories Information System (BExIS; http://doi.org/10.17616/R32P9Q). In forests, LUI was quantified with the Forest Management Intensity (ForMI) index (*63*), which was derived from two inventories (2008–2011 and 2014–2016) of living trees, stumps, and dead wood and consists of three components: the proportion of harvested tree volume, the proportion of tree species that are not part of the natural forest community, and the proportion of dead wood showing signs of saw cuts. To measure LUI, plot-specific values of the ForMI index were extracted from BexIS and averaged over the two inventories. Note that, between 2008 and 2020 (time span of our study), LUI in the plots remained largely constant in both grasslands and forests, leaving the relative differences between plots unaltered (see section S1A).

### Data collection

#### Vegetation sampling

In grasslands, vegetation was recorded on 150 permanent plots of 4 m by 4 m once a year (mid-May to mid-June, simultaneously in all regions) from 2008 to 2020. In forests, annual surveys were conducted on 150 permanent plots of 20 m by 20 m twice a year, in spring and summer from 2009 to 2020. The two records were combined using the higher cover value for each species. The use of different plot sizes for the two habitats follows general recommendations and common practices for assessing vegetation in grassland and forest understory (*64*, *65*). For the following analyses, we only considered plant species found in the herbaceous layer, because analyses of community dynamics in tree and shrub layers usually require longer time series and arthropod data were only available for this stratum. Because we only retained the herbaceous layer, six forest plots (located in particularly poor understories) had zero cover in several years. Therefore, we filtered them out to keep only plots with any species present in the herbaceous layer in at least 8 years (corresponding to two-thirds of the time-series length in forests). Species cover (used as a proxy for plant abundance) was visually estimated as percentage cover. Specifically, the ground cover of each vascular plant species was estimated in 1% increments. To ensure consistency and minimize interobserver bias, field-work participants received a joint training and worked in pairs, especially in the first days. Additional details on vegetation sampling are included in section S1B.

#### Arthropod sampling

Arthropods were sampled on 150 grassland and 30 forest sites annually from 2008 to 2017 (*66*). On the remaining 120 forest plots, arthropods were only sampled every third year (in 2008, 2011, 2014, and 2017), and these plots were thus excluded from the analysis. In grasslands, arthropods were sampled in June and August by sweep netting along three 50-m-long plot-border transects, with 60 double sweeps per plot (*67*). Sweep netting was only conducted on days without rain, low wind speed, and after morning dew had dried. In forest plots, arthropods were sampled using two flight-interception traps placed close to two randomly selected corners of each plot and operating from March to October. Traps consisted of two crossed transparent plastic shields (40 cm by 60 cm) with funnels opening into sampling jars below and above the shields, filled with a 3% copper sulfate solution and a drop of detergent. Sampled arthropods were stored in 70% ethanol and sorted to the order level in the laboratory. Adult specimens of Coleoptera, Hemiptera (Heteroptera and Auchenorrhyncha) (forest and grasslands), Orthoptera, and Araneae (both only grasslands) were then identified by taxonomic experts (*68*–*70*). These groups represent on average 20 and 40% of all sampled arthropod individuals (including juveniles and nontarget taxa such as Diptera) in forest and grasslands, respectively. Individuals not identified to the species level were excluded from the analyses. These represent 0.01% of all sampled individuals in forests and 8.7% in grasslands and are mostly female Hemiptera belonging to the family Cicadellidae. For the analysis, data from different sampling dates and traps were pooled per plot and year. To check the congruence of results and to address our question related to multi-trophic interactions, we not only analyzed all arthropods together but also performed separate analyses for herbivores and carnivores.

#### Functional traits

Because of our focus on broad taxonomic units (total arthropods) and trophic groups (herbivores and carnivores), a traditional “trait-matching” approach would be conceptually inappropriate and practically difficult to implement. Therefore, we selected traits that are broadly important across various groups (e.g., body size for arthropods) and largely indicative of community-level ecological strategies in response to resource availability and disturbance. For plants, we used trait data on plant height (meters), SLA (square meters per kilogram), LDMC (milligrams per gram), seed mass (milligrams), leaf nitrogen (milligrams per gram), and phosphorus (milligrams per gram) content. These traits are associated with growth, resource acquisition, life history, and reproductive strategies (*31*, *49*), and their potential to predict population and community stability has been suggested by several studies (*7*, *10*, *18*, *40*). Because of limited data availability for informative belowground plant traits (and especially root traits), these could not be included in the analysis. Concerning arthropods, we collected information on the following six morphological and ecological traits: mean body size (millimeters), variation in body size (%), dispersal ability (0 to 1 by steps of 0.25), feeding guild (main food source during the larval and adult stage: herbivores, predators, fungivores, detritivores, and omnivores), feeding mode (describing the way nutrients are ingested: extraintestinal digestion, chewing, and sucking), and vertical stratum use (shrub/tree dwellers, herb dwellers, ground dwellers, soil dwellers, species linked to water bodies, and unspecific). These traits also play a key role in species response to resource availability and disturbance (*71*) and are, therefore, expected to directly respond, alongside plant traits, to LUI. At the same time, evidence suggests that the selected plant traits, particularly leaf traits related to nutrient content and toughness, can influence herbivory and, consequently, affect arthropod abundance both directly (*72*) and indirectly, in line with our second hypothesis (H2), by acting on the selected arthropod traits.

Details on trait data sources and building of a trait matrix are provided in section S2A. In case of multiple records, values were averaged within species.

### Data preparation

For each plot, community stability was quantified as the inverse of the coefficient of variation (i.e., μ/σ, corresponding to the ratio between the mean and the SD; see Box 1) for total vegetation abundance (measured as sum of percentage cover of all individual species) or the total abundance of arthropods over time. The first reflects the stability of vegetation structure over time and is closely related to several ecosystem functions including productivity, litter and soil organic matter production, evapotranspiration, soil erosion and flood regulation, and pollination. The use of cover instead of biomass to quantify stability in plant communities reflects a trade-off between data availability and consistency of the framework. Because biomass data were only available in grasslands (and for a shorter time series), using a cover-derived stability metric allows for consistent analyses and comparisons across habitats. It also enables us to interpret our findings in light of one of the few studies in real-world ecosystems that considers indirect effect of LUI in grasslands and forests (*21*). Last, and more generally, it follows a substantial body of literature using stability metrics derived from vegetation attributes different than biomass (e.g., plant cover) (*5*, *8*, *39*, *40*). We then computed plot-specific biotic features as follows.

#### Community features

Mean species richness, often used as proxy for the averaging or portfolio effect (*5*, *7*, *8*) was computed as the average number of species found in each plot over the time series. Mean total abundance, which is the numerator in the stability formula (see Box 1) and thus inevitably influences stability (*7*, *21*), was calculated as the average total abundance found in each plot over the years. For community synchrony, we used the 𝜂_w_ index as implemented in (*21*), which is defined as the mean correlation coefficient between the abundances of each species (weighted by their relative total abundances over all years) and the rest of the community. wAPV (summarizing dominant species variability) was quantified, for each plot, as the sum of all species’ coefficient of variations multiplied by their mean relative abundances over the time series (*30*). In forests, we also computed mean tree cover as the average total cover recorded for the species belonging to the two tree layers (big trees and small trees).

#### Functional features

For each plot, functional diversity and dominant species traits (see Box 1) were calculated on pooled communities where each species was present with the mean relative abundance shown across the time series. Functional diversity was quantified using Rao’s *Q* index (*73*) applied to a dissimilarity matrix. Species dissimilarity was computed using the gawdis function in the gawdis package (*74*), which allows accounting for unequal trait contribution when computing Gower’s distance. By assessing the multivariate divergence in trait space, the multi-trait metric Rao’s *Q* allows capturing the global differentiation across a comprehensive set of strategies within a community. This allows testing the hypothesis that diversified strategies within a community lead to varied responses to environmental perturbations, resulting in asynchronous species fluctuations (i.e., reduced community synchrony). To define the dominant species traits, we used the first two axes of a PCA performed (separately for grasslands and forests) on the correlation matrix of the plot-specific CWM values of the abovementioned traits. Note that, for qualitative traits (feeding guild, feeding mode, and vertical stratum use in arthropods), the CWM represents the proportion of a given category of the trait in the community. Also note that, for arthropods, using the same set of traits to compute functional diversity and dominant species traits resulted in highly correlated values of the metrics. We thus decided to use morphometric traits and dispersal ability to compute functional diversity while using traits related to feeding preferences and habitat suitability to extract the dominant species traits. Results of the PCA performed on individual taxonomic groups and trophic levels, as well as further discussion on the ecological mechanisms underlying the relationship between plant traits, trait indices, and total vegetation abundance, are provided along with additional details in sections S2B to S2D. PCAs were performed using the rda function in the vegan package (*75*). Where necessary, traits were log transformed to meet normality criteria (*76*).

### Statistical analysis

To investigate the relative influence of the different drivers of stability, explore their interrelationships, and quantify the mediating role of functional features, we fitted piecewise SEMs using the psem function in the piecewiseSEM package (*77*). SEMs are powerful tools for testing conceptual models, quantifying the relative importance of explanatory variables and disentangling direct versus indirect effects (see Box 1) (*77*, *78*). Specifically, on the basis of our two broad hypotheses (H1 and H2), we conducted an exploratory path analysis by first building nearly saturated hypothetical SEMs and then using a model selection approach based on information theory to allow for the identification of the links that best explain the endogenous variables. To test H1, we fitted a total of eight SEMs: four main SEMs (two for plants and two for arthropods in grasslands and forests) and four additional SEMs to explore relationships in arthropod herbivore and carnivore communities (in grasslands and forests). Hypothetical causal models (meta-models) were built on the basis of a priori knowledge of the study systems (and grounded in the literature). In all SEMs, we included direct links from functional diversity and dominant species traits to community stability, as well as indirect paths through acknowledged stability drivers (species richness, synchrony, and dominant species variability) and through mean total abundance, due to its mathematical relationship to stability (*16*). Similarly, we tested both a direct land use–stability link and indirect connections via all other variables. In forests, we also accounted for the effect of mean tree cover (by including direct links to all variables except LUI), given its influence on understory diversity (*79*). To test for LUI-driven multi-trophic effects (H2) in total arthropod, herbivore, and carnivore communities (in grasslands and forests), we fitted six additional SEMs. Here, we included plant functional features (functional diversity and dominant species traits) as mediators (see Box 1) of LUI by specifying direct paths to stability as well as to arthropod community and functional features. For plants, we also explored the effect of soil moisture (here used to account for differences in soil type) on mean total vegetation abundance. However, because this pathway was discarded during model selection (see below), soil moisture was not included in the final set of predictors (see section S1C). Plant SEMs contained partial correlations between functional diversity and dominant species traits. Arthropod SEMs included partial correlations between functional diversity and richness. Before the analysis, variables were mean centered and scaled to 1 SD to allow direct comparison of effect sizes between predictors. SEMs were fitted using linear models (LMs). During model validation, missing paths (i.e., previously unconsidered significant relationships) were evaluated and included if considered causal, or otherwise left to freely covary. Fisher’s *C* (*P* > 0.05) was used to evaluate model fit. For each response variable, we fitted an a priori SEM (built according to the meta-model) and then fitted a reduced SEM resulting from a model selection process implemented as follows. For each of the constituent LMs in each SEM, we fitted all possible submodels including different combinations of the fixed effect terms using the dredge function [package MuMIn; (*80*)]. We then computed, for each term, the sum of Akaike weights and used the evidence ratio as a measure of the relative importance of variables, retaining those variables with an evidence ratio > 2.72, i.e., with a likely effect (*81*, *82*). A priori and reduced SEMs were then compared using corrected Akaike Information Criterion, which is a second-order variant of Akaike information criterion corrected for small *n*/*K* ratios (*81*). Bootstrapped standardized effects were computed for each SEM using package semEff (functions bootEff and semEff) (*83*), which allows extracting direct, indirect, mediator, and total effects (see Box 1). All SEM constituent models were checked for normality, homoscedasticity, and multicollinearity using the performance R package (function check_model) (*84*).

## Supplementary Materials

### This PDF file includes:

Sections S1 to S8 Figs. S1 to S15 Tables S1 to S29 References
