# Supplementary material for: Functional traits mediate the effect of land use on drivers of community stability within and across trophic levels
Source: Sci Adv. 2025 Jan 24;11(4):eadp6445. doi: 10.1126/sciadv.adp6445 (PMC11759044; doi:10.1126/sciadv.adp6445)
Supplement: Supplementary file 1 — Sections S1 to S8 Figs. S1 to S15 Tables S1 to S29 References [file sciadv.adp6445_sm.pdf]

Science Advances
AAAS

Supplementary Materials for

Functional traits mediate the effect of land use on drivers of community stability within and across trophic levels

Marta Gaia Sperandii *et al.*

Corresponding author: Marta Gaia Sperandii, mg.sperandii@gmail.com

Sci. Adv. **11**, eadp6445 (2025)
DOI: 10.1126/sciadv.adp6445

This PDF file includes:

Sections S1 to S8
Figs. S1 to S15
Tables S1 to S29
References

S1A. Plot-specific variation of land-use intensity over time

During the timespan of our study (2002-2008), land-use intensity remained largely constant in both grasslands and forests. Figure R1 shows the distribution of the coefficient of variation (CV) for the land-use intensity and forest management indices (Fig S1.1). While the distribution shows a slight right skew in forests compared to grasslands, the median CV values are very similar: 0.224 for grasslands and 0.222 for forests. This indicates that while land-use intensity was not completely constant, it remained stable enough that we expect the ranking of communities to have stayed relatively unchanged over time. Note that, whereas land-use intensity values are collected annually for grasslands, for forests they were derived from two inventories, which makes the use of the CV less meaningful in the latter case. However, considering the long-term and inherently more stable nature of forest ecosystems, particularly within the context of the Biodiversity Exploratories, we believe there were no significant changes in land-use intensity that alter the relative differences between plots. This is also suggested by (*85*), who reported that changes in stand structure (which are significantly influenced by forest management) were not substantial and were primarily observed in younger stands. Regarding grasslands, our findings align with previous research (*86*), who identified only weak within-plot trends in land-use intensity over time.

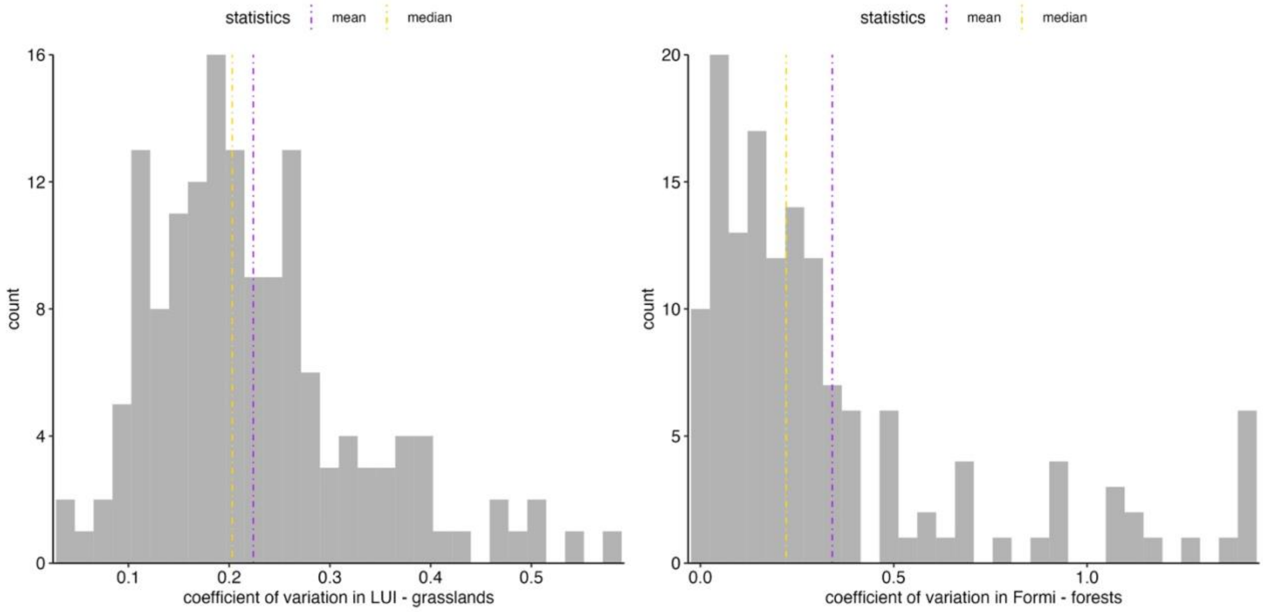

Figure S1. Distribution of the coefficient of variation (CV) for the grassland land-use intensity (LUI, left) and forest management intensity (Formi, right).

S1B. Vegetation sampling

The Biodiversity Exploratories were established in three different regions of Germany: the UNESCO Biosphere Reserve Schorfheide-Chorin (North-East Germany), the National Park Hainich and its surrounding areas (Central Germany), and the UNESCO Biosphere Reserve Schwäbische Alb (South-West Germany). Each region includes "most of the variation in land use typical for grasslands and forests in Germany, from hardly managed grasslands and unmanaged beech forests to highly fertilized and intensively used meadows and pastures, and intensively managed forests" (*41*). Within each region, three hierarchical levels of study plots were established. In this study, we focus on the so-called experimental plots (EPs). Following a stratified random sampling design, 100 EPs were established, 50 of which in grasslands and 50 in forest areas. The strata were built as to cover variation in land-use intensity and soil depth, while ensuring consistency of soil type, homogeneity of land use and vegetation composition within plots, slope lower than 20% and absence of water-logging (*41*). EPs have a size of 50 × 50 m in grasslands and 100 × 100 m in forests. Within each EP, vegetation was recorded in permanent plots of 4 x 4 m in grasslands and 20 x 20 m in forests. These plot sizes follow general recommendations and widely used practices for sampling vegetation (*45-46*). More specifically, these sampling areas are considered adequate to representatively cover the vegetation of grasslands and forests, while at the same time allowing careful consideration of all occurring plant species. Vegetation abundance was estimated using plant cover. Each vascular species present in a plot was recorded following the nomenclature of Wisskirchen & Häupler (*87*). In particular, ground cover was estimated for each species as percentage cover in 1% steps to be as accurate as possible. For very low cover values (below 1%), percentage cover was estimated as either 0.1% or 0.5%. This procedure has proven suitable in several previous analyses (*28, 88-91*) and, despite inevitable methodological errors, it is considered much more accurate than using abundance classes (*92-93*). Species cover was estimated by trained staff that received a joint training to gain comparable results within and among regions. Field work teams were kept as stable as possible over time. There were, however, for logistical reasons, different people involved in abundance estimations. Participants were experienced and, especially during the first days of sampling, cover values were estimated by groups of two people to calibrate against each other and therefore get the most accurate estimations and minimise interobserver bias.

S1C. Influence of soil type on mean total vegetation abundance

According to the experimental design used within the Biodiversity Exploratories, grassland and forest sites in all three regions were selected to cover a similarly large gradient in land-use intensity, while minimising variation in confounding factors such as soil type and spatial distribution (*41*). However, soil characteristics can be key in shaping total vegetation abundance, especially in open vegetation types such as grasslands, where different soils (e.g. shallow loamy soils vs sandy soils), can lead to varying degrees of vegetation cover. To address this issue, we ran the hypothesised grassland plant SEM including mean soil moisture (used here as a proxy for differences in soil type) as an exogenous predictor of mean total vegetation abundance, and included in the SEM a partial correlation between soil moisture and land-use intensity. Based on the test of direct separation (which evaluates important missing paths), we also added a causal link between PC2 (summarising a trade-off between communities dominated by tall vs short plants) and soil moisture. However, the model selection discarded the direct path between soil moisture and mean total vegetation abundance, suggesting that soil moisture did not significantly contribute in explaining patterns of mean total vegetation abundance. Besides, the effect of soil moisture was not statistically significant also in the hypothesised model. For this reason, and because testing the effect of soil moisture on PC2 was beyond the scope of this analysis, soil moisture was therefore not included in the final SEM (see Figure S1.2 below, showing the result of the reduced SEM including soil moisture).

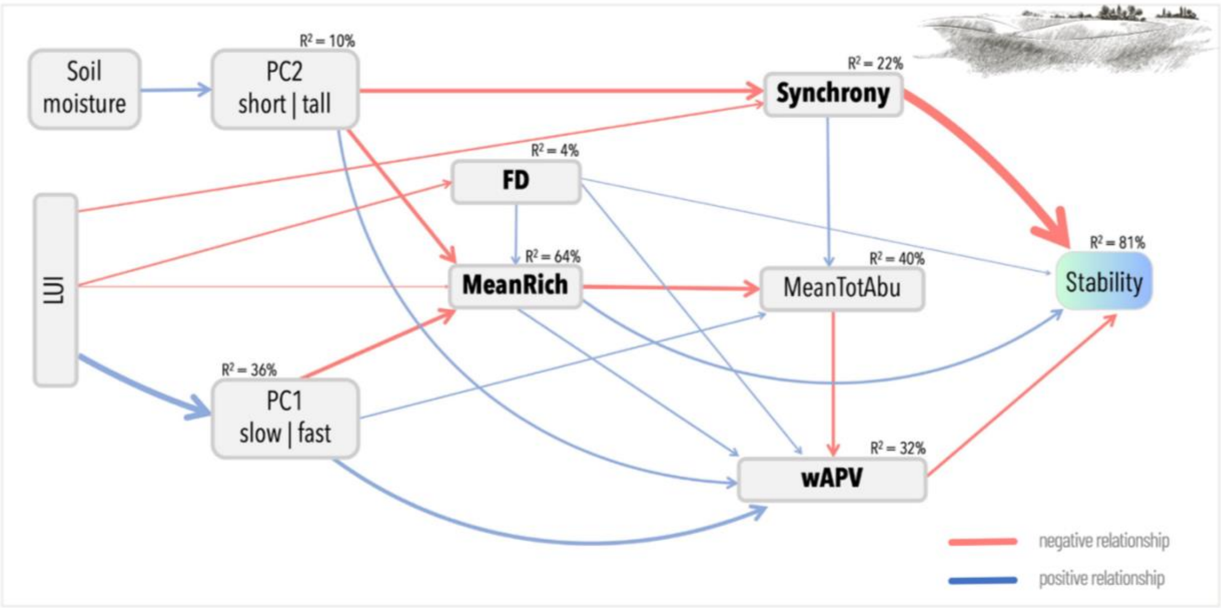

Figure S2. Results of the reduced Structural Equation Model (SEM) including soil moisture. The thickness of the arrows is proportional to the slope of the relationship. The model showed a good fit (Fisher's C = 44.276, df = 42, P = 0.376, n = 150). PC1 and PC2: First and second PCA axes used to define the dominant species traits. MeanRich: mean species richness. FD: functional diversity. MeanTotAbu: mean total abundance. wAPV: weighted average population variability. Top right drawing: canva.com.

S2A. Selecting functional traits and building the trait matrix

For plant traits, we used a combination of data measured on plants from the study plots (when available) and literature data. Measured values (*94-95*) were only available for LDMC and SLA, and for 287 out of the 590 species recorded). The trait matrix was built as follows: for LDMC and SLA, we gave preference to measured values and, when these were not available, we filled the gaps with data from the TRY database (*96*), cleaned and aggregated at the species level (*97*). Values for the other traits (plant height, seed mass, leaf nitrogen and leaf phosphorus) were extracted from Bexis dataset 27610 (*97*). Note that for LDMC and SLA, we checked the Pearson correlation between measured and literature data, which was 0.77 for SLA and 0.74 for LDMC.

For arthropod traits, selected traits (mean body size, variation in body size, dispersal ability, feeding guild, feeding mode, and vertical stratum use) were retrieved using published datasets (*98-101*). Whereas for plants, trait-based community attributes (dominant species traits and functional diversity) were all computed on the same set of traits, this was not possible for arthropods, as using the same set of traits resulted in extremely high correlated values of functional diversity and dominant species traits. We thus decided to use morphometric traits (mean body size, variation in body size) to compute functional diversity, while using traits related to feeding preferences and habitat suitability (feeding guild, feeding mode and stratum use) to extract the dominant species traits.

S2B. Results of the PCA performed on individual taxonomic groups and trophic levels. Figures S3 to S6 include biplots and correlation matrices showing the correlation between the first two axes of the Principal Component Analysis (PCA) and the community weighted mean (CWM) values of the functional traits included in the PCA for plants, total arthropods, herbivores and carnivores in grasslands and forests. Crossed cells represent Pearson correlation coefficients not significant at $p < 0.05$.

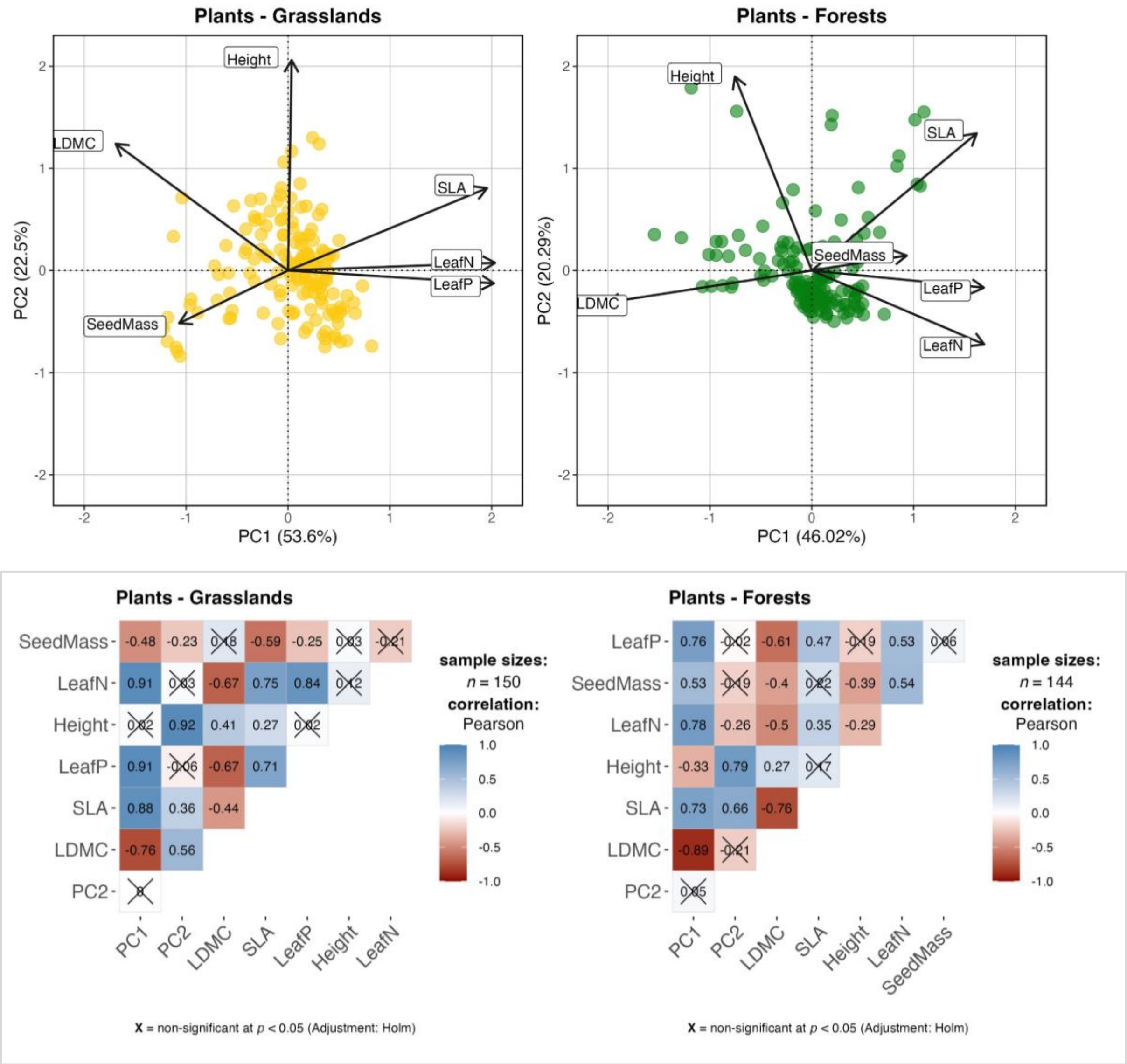

Figure S3. Biplots and correlation matrices showing the correlation between the first two PC axes and the CWMs of the functional traits for plants in grasslands and forests. Crossed cells represent Pearson correlation coefficients not significant at $p < 0.05$. SLA: specific leaf area; LDMC: leaf dry matter content; LeafN: leaf nitrogen; LeafP: leaf phosphorus; Height: plant height.

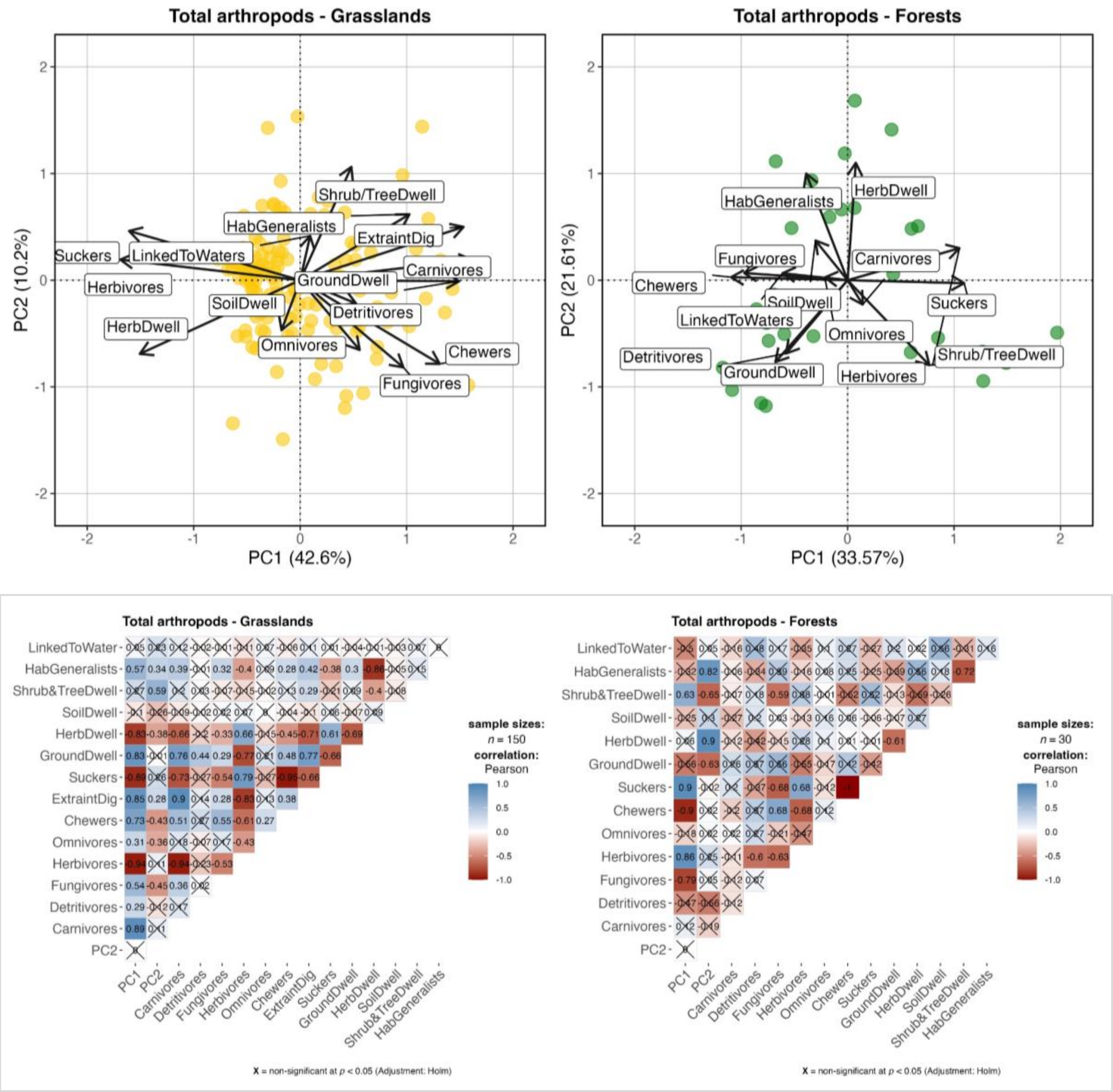

Figure S4. Biplots and correlation matrices showing the correlation between the first two PC axes and the CWMs of the functional traits for total arthropods in grasslands and forests. Crossed cells represent Pearson correlation coefficients not significant at $p < 0.05$.

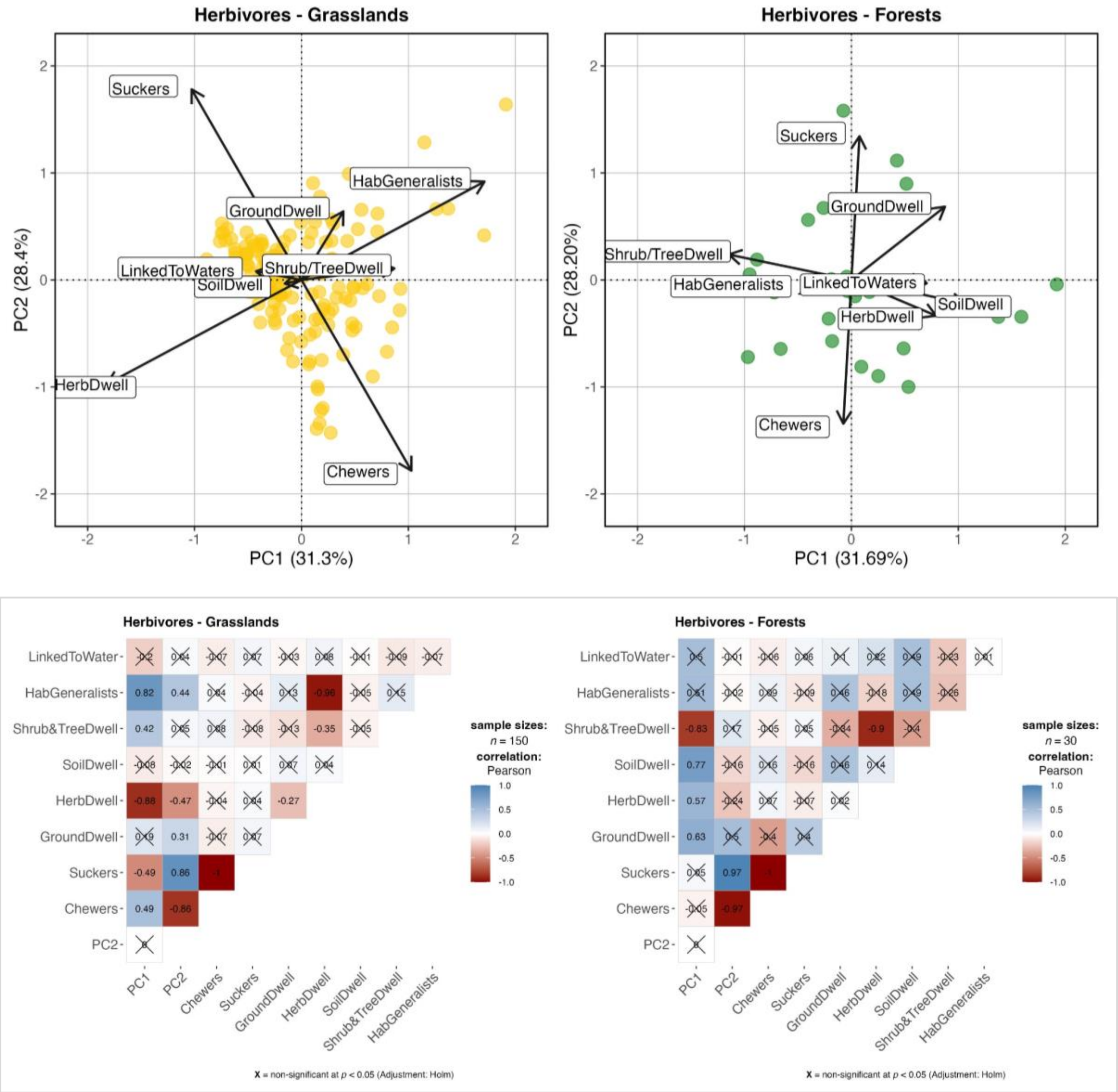

Figure S5. Biplots and correlation matrices showing the correlation between the first two PC axes and the CWMs of the functional traits for herbivores in grasslands and forests. Crossed cells represent Pearson correlation coefficients not significant at $p < 0.05$.

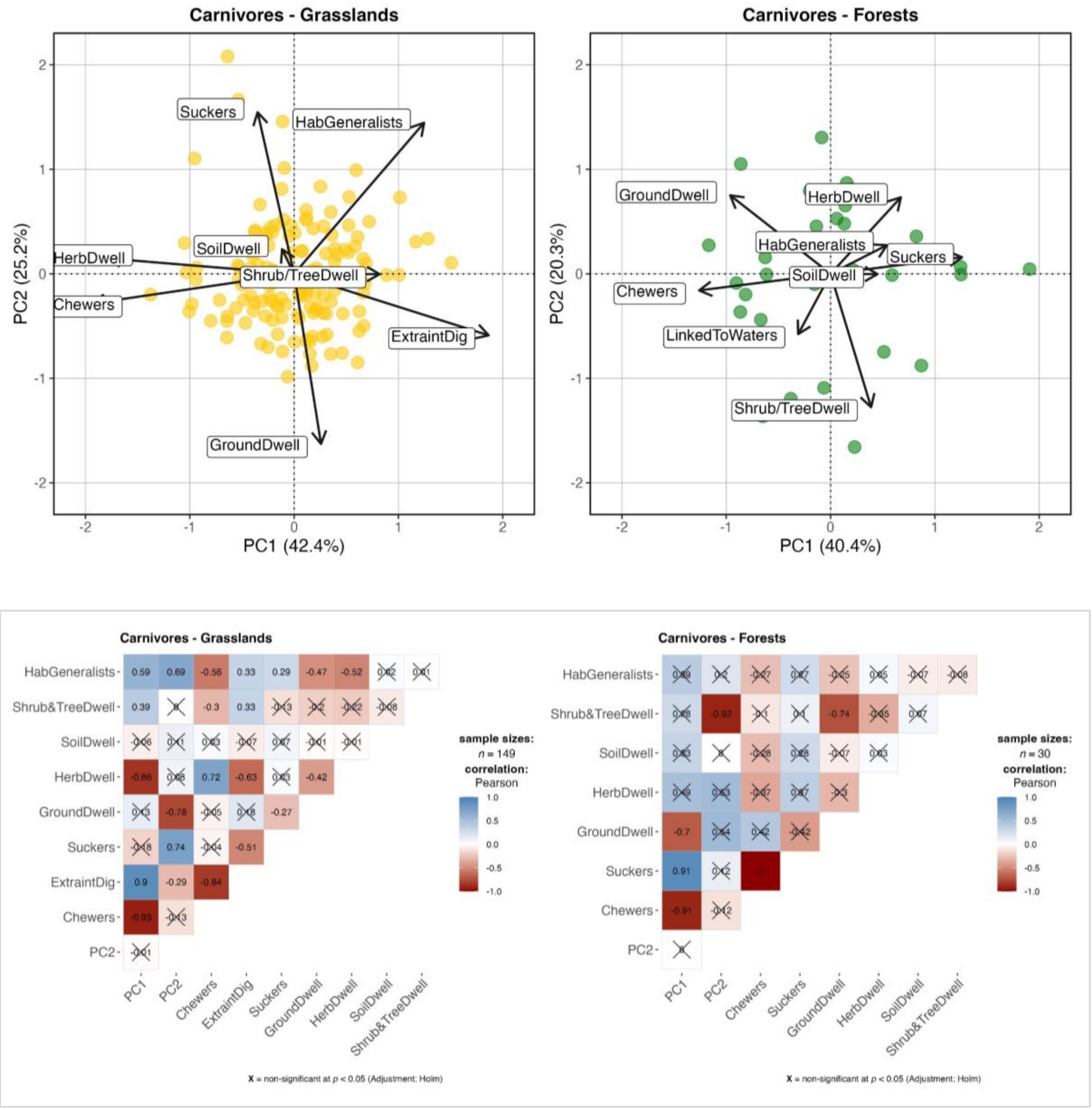

Figure S6. Biplots and correlation matrices showing the correlation between the first two PC axes and the CWMs of the functional traits for carnivores in grasslands and forests. Crossed cells represent Pearson correlation coefficients not significant at $p < 0.05$.

S2C. Interpretation of the PCA performed on individual taxonomic groups and trophic levels. Figures S7 to S10 include the interpretation, and explained variance of the first two Principal Component (PC) axes ran on plot-specific CWM values for individual taxonomic/trophic groups (plants, total arthropods, herbivores and carnivores) in grasslands and forests.

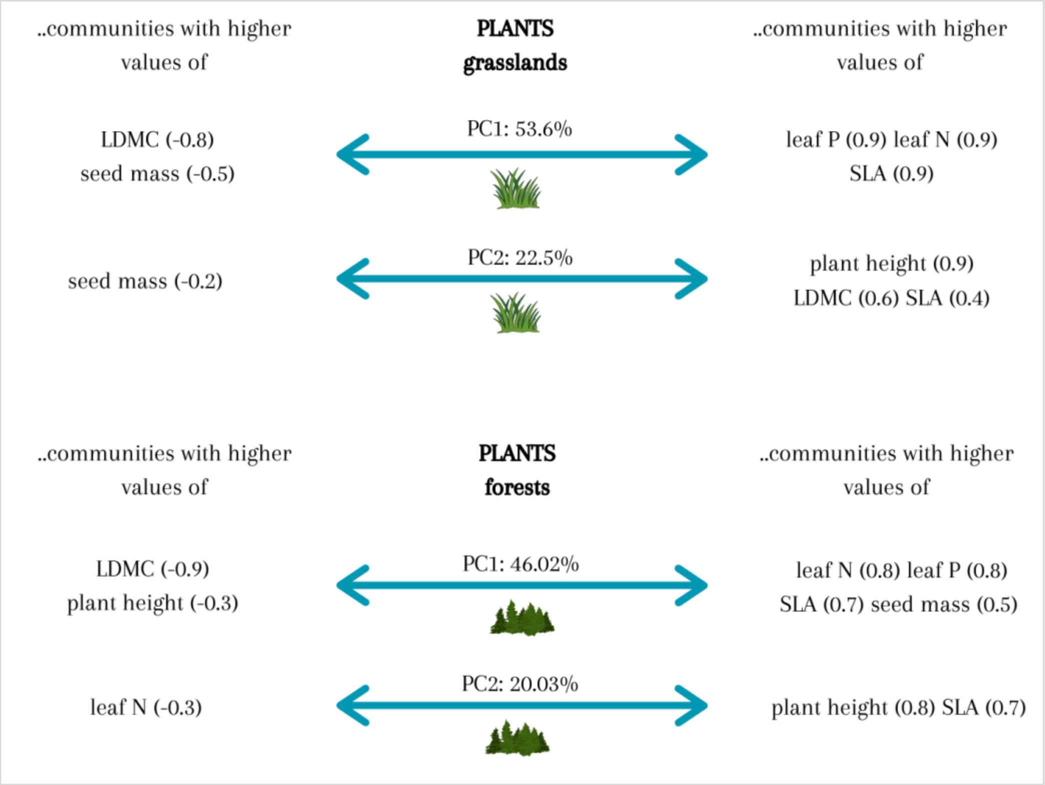

Figure S7. Interpretation, and explained variance of the first two Principal Component (PC) axes ran on plot-specific CWM values for plants in grasslands and forests. Only significant Pearson correlations (see S2B) are reported in the tables. For each variable, the Pearson correlation value is reported within brackets.

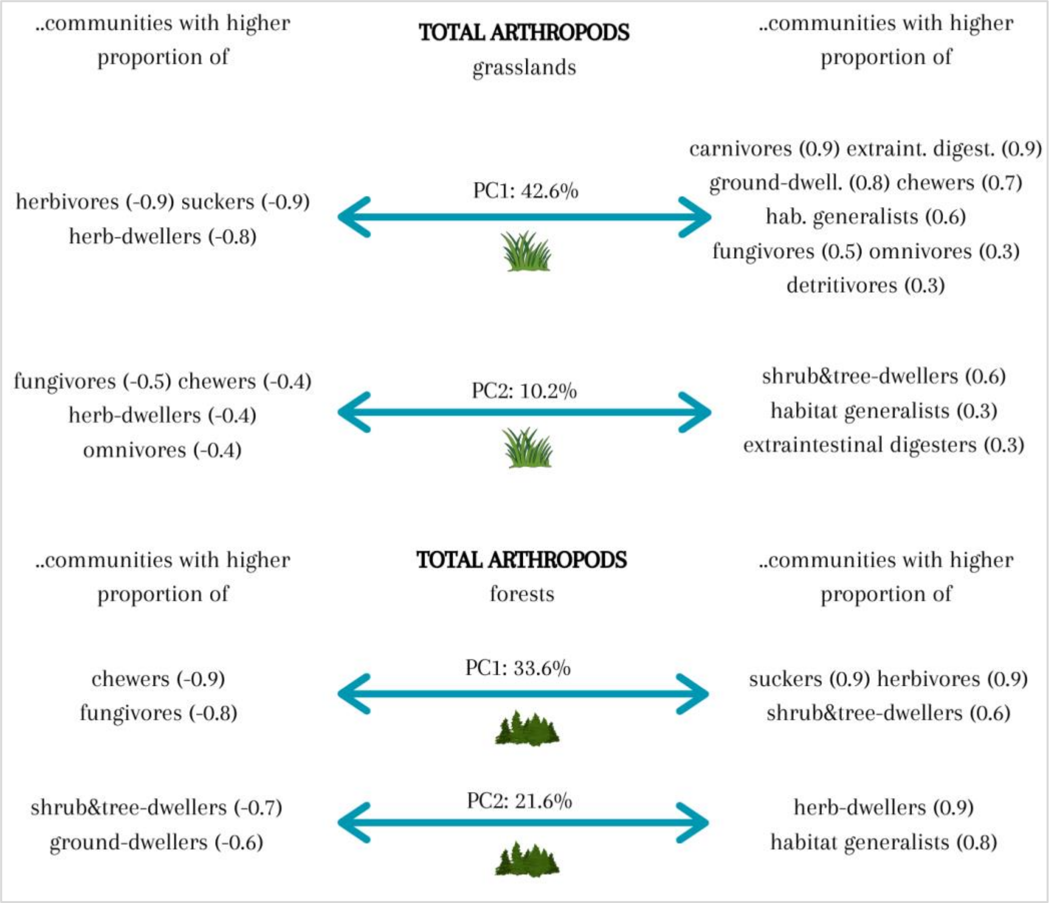

Figure S8. Interpretation, and explained variance of the first two Principal Component (PC) axes ran on plot-specific CWM values for total arthropods in grasslands and forests. Only significant Pearson correlations (see S2B) are reported in the tables. For each variable, the Pearson correlation value is reported within brackets.

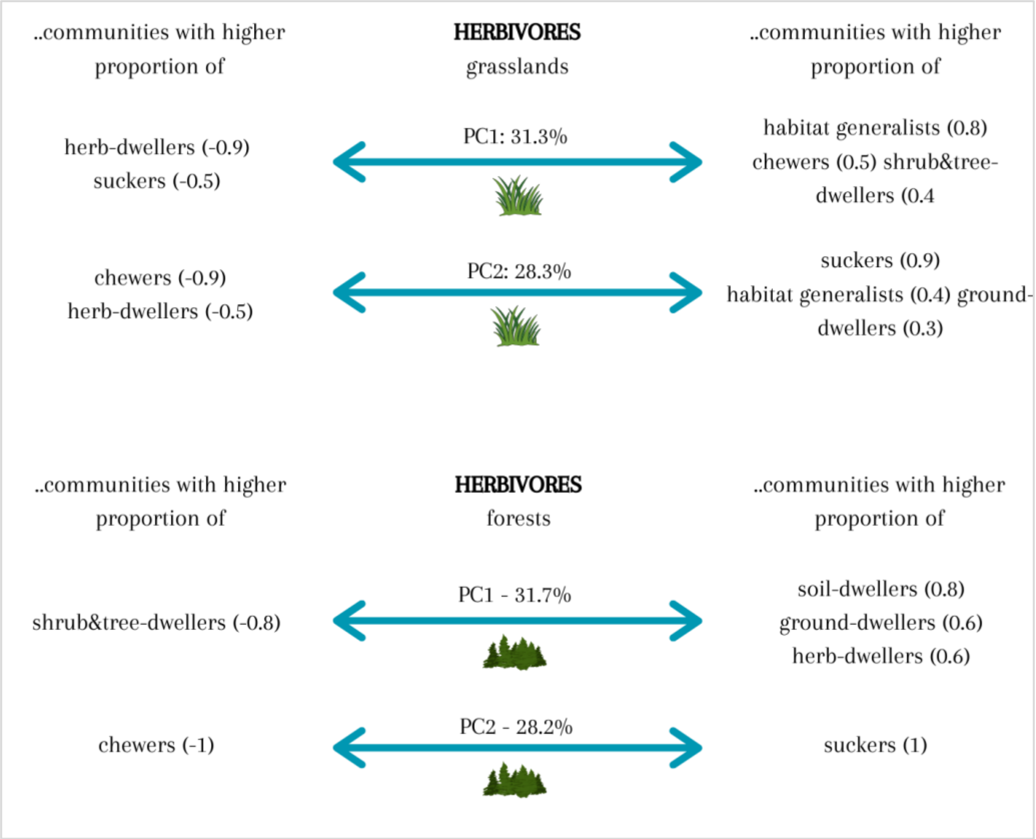

Figure S9. Interpretation, and explained variance of the first two Principal Component (PC) axes ran on plot-specific CWM values for herbivores in grasslands and forests. Only significant Pearson correlations (see S2B) are reported in the tables. For each variable, the Pearson correlation value is reported within brackets.

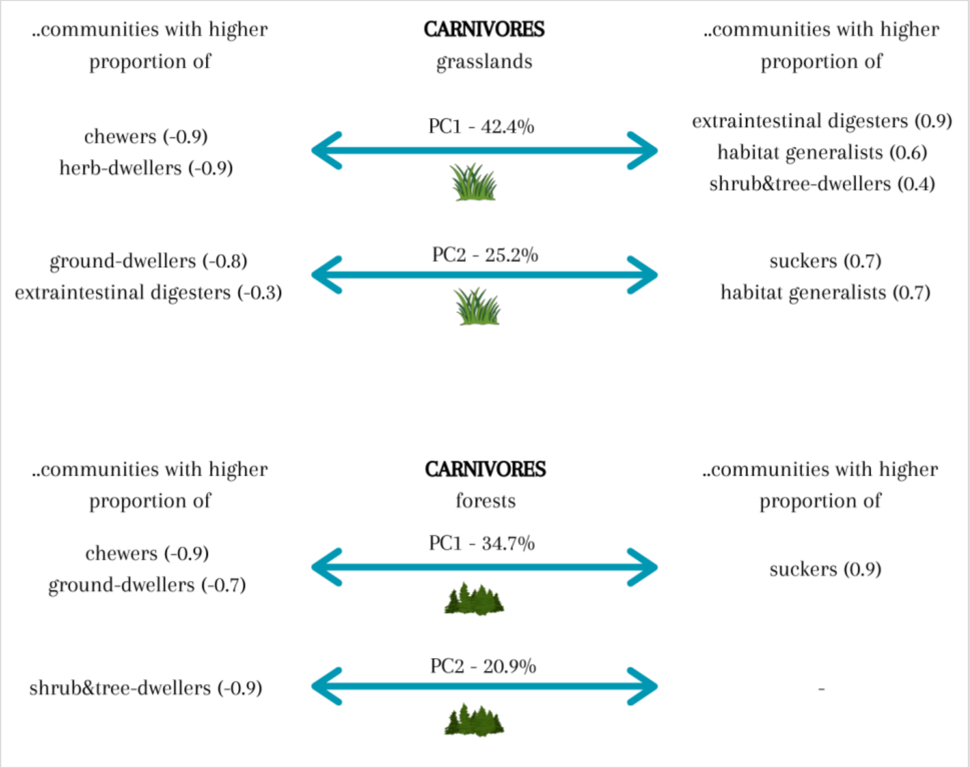

Figure S10. Interpretation, and explained variance of the first two Principal Component (PC) axes ran on plot-specific CWM values for carnivores in grasslands and forests. Only significant Pearson correlations (see S2B) are reported in the tables. For each variable, the Pearson correlation value is reported within brackets.

S2D. Ecological mechanisms underlying the relationship between trade-offs along the leaf-economics spectrum and total vegetation abundance

Ecosystem properties such as total plant cover (used in our study as a proxy for total vegetation abundance) are mechanistically linked to trait indices, and exploring this link allows understanding ecological patterns (*102-103*).

In our dataset, a positive correlation ($r = 0.49$, $t = 6.8341$, $df = 148$, p-value < 0.001) exists between the first principal component (PC1, representing the leaf-economics spectrum as detailed below) and mean total cover. This reflects an ecological mechanism, as further explained. According to the mass-ratio hypothesis, originally proposed by Grime (*17*), dominant species traits can affect total community abundance. In our study, this effect would extend to average community abundance over time, which is the denominator of the coefficient of variation (CV, our measure of stability). This effect is expected to be especially driven by traits associated with the leaf-economics spectrum (e.g., specific leaf area, leaf dry matter content, leaf nitrogen and phosphorus content).

Although the mass-ratio hypothesis was initially formulated in the context of plant traits and biomass, plant cover and biomass are closely related in many ecosystems (*104-107*), making cover a reliable, frequently-used proxy for biomass, especially in non-experimental or large-scale ecological studies (*108*). The two quantities were positively correlated also in our grassland dataset ($r = 0.68$, $t = 11.146$, $df = 148$, p-value < 0.001).

In our study, as also illustrated in S2B and S2C, the first axis of the PCA run on the plot-specific community weighted means (PC1) can be considered a proxy of the leaf-economics spectrum. Positive values of PC1 represent plant communities (i.e. plots) characterised by acquisitive strategies (high values of SLA, leaf nitrogen and leaf phosphorus content), and thus featuring high relative growth and net photosynthetic rates. Conversely, negative PC1 values are associated with plant communities characterised by species with conservative strategies (high LDMC). Hence, the positive relationship between PC1 and mean total cover we observed indicates that plant communities characterised by acquisitive strategies also exhibit higher total cover over time and, given the above-mentioned positive relationship between cover and biomass in our dataset, they are more productive.

S3. Tables illustrating the effects of direct stability drivers on the stability of different taxonomic and trophic groups across various habitats, along with the total effects of land-use intensity on stability.

Table S1. Direct stability drivers for plant and arthropod communities in grasslands (GR) and forests (FOR). MeanRich: mean species richness; wAPV: weighted average population variability; MeanTotAbu: mean total abundance; FD: functional diversity; PC1 and PC2: first and second axes of the Principal Component Analysis ran on community weighted mean values to characterise the dominant species traits. For each driver, its bootstrapped standardised direct effect on stability is reported.

Group	**Hab**	**Synchrony**	**MeanRich**	**wAPV**	**MeanTot Abu**	**FD**	**PC1**	**PC2**
Plants	GR	-0.889	0.264	-0.288		0.112		
	FOR	-0.546		-0.426	0.207	0.158		
Total arthropods	GR	-0.546		-0.461	-0.102			
	FOR	-0.632	0.486	-0.443	-0.281			
Arthropod herbivores	GR	-0.554		-0.494	-0.084			
	FOR	-0.409		-0.630			0.347	0.528
Arthropod carnivores	GR	-0.736	0.339	-0.161	-0.314			
	FOR	-0.715	0.233	-0.416	-0.309			

Table S2. Bootstrapped total effect (i.e. the sum of direct and indirect effects) of direct drivers of stability (i.e. those reported in S3A) and of land-use intensity on community stability for plants and arthropods in grasslands (GR) and forests (FOR). Non-significant effects (i.e. effects where confidence intervals include zero) are shown in light grey. MeanRich: species richness; wAPV: weighted average population variability; MeanTotAbu: mean total abundance; FD: functional diversity. PC1 and PC2: first and second axes of the principal component analysis ran on community weighted mean values to characterise the dominant species traits; MeanTreeCov: mean tree cover (only analysed in forests); LUI: land-use intensity.

**Group**	**Hab**	**Synchrony**	**Mean Rich**	**wAPV**	**MeanTot Abu**	**FD**	**PC1**	**PC2**	**LUI**
Plants	GR	-0.868	0.168	-0.288		0.099			0.058
	FOR	-0.546		-0.426	0.348	-0.129			0.021
Total arthropods	GR	-0.609		-0.461	-0.187				-0.104
	FOR	-0.632	0.283	-0.443	-0.281				0.024
Arthropod herbivores	GR	-0.603		-0.494	-0.171				-0.051
	FOR	-0.409		-0.630			0.525	0.528	-0.072
Arthropod carnivores	GR	-0.736	0.020	-0.161	-0.314				0.029
	FOR	-0.715	-0.026	-0.416	-0.309				-0.085

Table S3. Bootstrapped total effect (i.e. sum of direct and indirect effects) of land-use intensity on direct stability drivers for plants and arthropods in grasslands (GR) and forests (FOR). Non-significant effects (i.e. effects where confidence intervals include zero) are shown in light grey. MeanRich: mean species richness; wAPV: weighted average population variability; MeanTotAbu: mean total abundance; PC1 and PC2: First and second axes of the PCA ran on community weighted mean values to characterise the dominant species traits.

Group	Hab	**Synchrony**	**MeanRich**	**wAPV**	**MeanTotAbu**	**FD**	**PC1**	**PC2**
Plants	GR	-0.220	-0.382	0.050		-0.196		
	FOR			0.076	0.099	0.206		
Total arthropods	GR	0.129		0.088	-0.064			
	FOR	0.009	0.075	-0.019	0.054			
Arthropod herbivores	GR	-0.018		0.131	-0.038			
	FOR			0.445				0.395
Arthropod carnivores	GR	-0.034	-0.177	-0.140	-0.130			
	FOR				0.276			

S4. **Results of the eight Structural Equation Models (SEMs) exploring the interplay between land use, acknowledged stability-driving mechanisms, and temporal stability *within* taxonomic groups (plants, arthropods) and trophic levels (herbivores, carnivores) in the two habitats.**

Table S4. Results of the SEM exploring the interplay between land use, acknowledged stability-driving mechanisms, and temporal stability for plants in grasslands. Results of the Fisher's C test are shown on top of the table. For each response variable within each model of the SEM, direct effects are reported as fully standardised model coefficients, and R^2 values are reported in brackets for each of the constituent models (whose name appears in bold). MeanTreeCov: mean tree cover. MeanRich: mean species richness. FD: functional diversity. MeanTotAbu: mean total abundance. wAPV: weighted average population variability. PC1 and PC2: First and second PCA axes used to define the dominant species traits.

Plants (grassland)

Fisher's C = 32.064 with P-value = 0.191 and on 26 degrees of freedom

Response variable	Predictor	Effect	Bias	Std. Err.	Lower CI	Upper CI	significance
**PC1 [R2: 0.36]**	LUI	0.599	-0.001	0.044	0.504	0.678	*
**MeanRich [R2: 0.64]**	LUI	-0.103	0.002	0.043	-0.190	-0.022	*
	PC2	-0.395	0.001	0.062	-0.517	-0.274	*
	PC1	-0.400	0.002	0.048	-0.491	-0.301	*
	FD	0.205	-0.005	0.056	0.097	0.315	*
**FD [R2: 0.04]**	LUI	-0.196	0.000	0.074	-0.335	-0.045	*
**synchrony [R2: 0.22]**	LUI	-0.220	0.002	0.071	-0.353	-0.074	*
	PC2	-0.423	0.000	0.074	-0.550	-0.254	*
**MeanTotAbu [R2: 0.40]**	PC1	0.187	-0.003	0.059	0.074	0.306	*
	MeanRich	-0.383	0.001	0.053	-0.484	-0.275	*
	synchrony	0.249	0.001	0.048	0.150	0.339	*
**wAPV [R2: 0.32]**	PC2	0.271	-0.004	0.072	0.128	0.410	*
	PC1	0.380	-0.010	0.077	0.225	0.522	*
	MeanRich	0.220	-0.004	0.071	0.083	0.358	*
	FD	0.166	-0.004	0.069	0.035	0.304	*
	MeanTotAbu	-0.297	0.003	0.076	-0.459	-0.159	*
**stability [R2: 0.81]**	MeanRich	0.264	0.000	0.043	0.190	0.363	*
	FD	0.112	-0.002	0.037	0.038	0.183	*
	synchrony	-0.889	0.010	0.020	-0.927	-0.857	*
	wAPV	-0.288	0.003	0.040	-0.380	-0.221	*

Table S5. Results of the SEM exploring the interplay between land use, acknowledged stability-driving mechanisms, and temporal stability for plants in forests. Results of the Fisher's C test are shown on top of the table. For each response variable within each model of the SEM, direct effects are reported as fully standardised model coefficients, and R^2 values are reported in brackets for each of the constituent models (whose name appears in bold). MeanTreeCov: mean tree cover. MeanRich: mean species richness. FD: functional diversity. MeanTotAbu: mean total abundance. wAPV: weighted average population variability. PC1 and PC2: First and second PCA axes used to define the dominant species traits.

Plants (forest)

Fisher's C = 39.647 with P-value = 0.311 and on 36 degrees of freedom

Response variable	Predictor	Effect	Bias	Std. Err.	Lower CI	Upper CI	significance
**PC1 [R2: 0.05]**	Mean tree cover	0.228	-0.004	0.090	0.052	0.398	*
**MeanRich [R2: 0.45]**	Mean tree cover	-0.302	0.004	0.067	-0.440	-0.177	*
	PC2	-0.348	0.009	0.068	-0.475	-0.217	*
	Formi	0.306	-0.004	0.060	0.186	0.423	*
	PC1	0.323	-0.004	0.068	0.190	0.455	*
**FD [R2: 0.15]**	Mean tree cover	-0.180	0.001	0.084	-0.344	-0.012	*
	Formi	0.206	0.001	0.074	0.057	0.348	*
**MeanTotAbu [R2: 0.65]**	Mean tree cover	-0.196	0.003	0.040	-0.277	-0.120	*
	PC2	-0.112	0.004	0.039	-0.193	-0.041	*
	PC1	0.201	-0.003	0.042	0.116	0.283	*
	MeanRich	0.445	-0.006	0.052	0.344	0.544	*
	FD	-0.181	0.006	0.042	-0.271	-0.105	*
**wAPV [R2: 0.53]**	PC1	0.135	-0.002	0.067	-0.006	0.260	
	FD	0.527	-0.003	0.049	0.427	0.621	*
	MeanTotAbu	-0.331	0.003	0.050	-0.428	-0.235	*
**Stability [R2: 0.76]**	synchrony	-0.546	0.003	0.046	-0.639	-0.460	*
	FD	0.158	0.000	0.043	0.078	0.249	*
	MeanTotAbu	0.207	-0.002	0.048	0.111	0.297	*
	wAPV	-0.426	0.009	0.057	-0.557	-0.330	*

Table S6. Results of the SEM exploring the interplay between land use, acknowledged stability-driving mechanisms, and temporal stability for total arthropods in grasslands. For each response variable within each model of the SEM, direct effects are reported as fully standardised model coefficients, and R^2 values are reported in brackets for each of the constituent models (whose name appears in bold). MeanTreeCov: mean tree cover. MeanRich: mean species richness. FD: functional diversity. MeanTotAbu: mean total abundance. wAPV: weighted average population variability. PC1 and PC2: First and second PCA axes used to define the dominant species traits.

Arthropods (grassland)

Fisher's C = 39.344 with P-value = 0.243 and on 34 degrees of freedom

Response variable	Predictor	Effect	Bias	Std. Err.	Lower CI	Upper CI	significance
**PC2 [R2: 0.08]**	LUI	-0.287	0.000	0.076	-0.424	- 0.126	*
**MeanRich [R2: 0.29]**	LUI	-0.274	0.002	0.069	-0.404	-0.133	*
	PC1	-0.462	0.003	0.056	-0.568	-0.349	*
**FD [R2: 0.31]**	PC1	0.560	0.000	0.049	0.446	0.642	*
**synchrony [R2: 0.13]**	LUI	0.226	-0.002	0.083	0.054	0.379	*
	PC2	0.341	0.000	0.066	0.195	0.458	*
**MeanTotAbu [R2: 0.65]**	PC1	-0.208	0.001	0.042	-0.297	-0.130	*
	MeanRich	0.394	0.004	0.059	0.274	0.504	*
	FD	-0.155	0.004	0.041	-0.230	-0.072	*
	synchrony	0.340	-0.004	0.048	0.239	0.425	*
**wAPV [R2: 0.56]**	PC1	0.294	-0.001	0.051	0.193	0.392	*
	MeanRich	-0.364	0.004	0.052	-0.467	-0.262	*
	FD	0.242	0.000	0.059	0.128	0.361	*
	MeanTotAbu	0.185	-0.002	0.050	0.094	0.289	*
**Stability [R2: 0.76]**	synchrony	-0.546	-0.003	0.047	-0.634	-0.450	*
	MeanTotAbu	-0.102	-0.001	0.044	-0.201	-0.027	*
	wAPV	-0.461	0.001	0.057	-0.591	-0.363	*

Table S7. Results of the SEM exploring the interplay between land use, acknowledged stability-driving mechanisms, and temporal stability for total arthropods in forests. For each response variable within each model of the SEM, direct effects are reported as fully standardised model coefficients, and R^2 values are reported in brackets for each of the constituent models (whose name appears in bold). MeanTreeCov: mean tree cover. MeanRich: mean species richness. FD: functional diversity. MeanTotAbu: mean total abundance. wAPV: weighted average population variability. PC1 and PC2: First and second PCA axes used to define the dominant species traits.

Arthropods (forest)

Fisher's C = 60.184 with P-value = 0.397 and on 58 degrees of freedom

Response variable	Predictor	Effect	Bias	Std. Err.	Lower CI	Upper CI	significance
**PC1 [R2: 0.20]**	Formi	-0.447	0.002	0.128	-0.662	-0.155	*
**PC2 [R2: 0.18]**	Formi	-0.421	-0.001	0.157	-0.671	-0.026	*
**MeanRich [R2: 0.53]**	Formi	-0.247	0.005	0.113	-0.439	0.008	
	PC1	-0.720	0.008	0.086	-0.852	-0.514	*
**FD [R2: 0.55]**	PC1	-0.458	0.009	0.090	-0.619	-0.273	*
	PC2	0.580	-0.003	0.109	0.291	0.747	*
**Synchrony [R2: 0.33]**	Mean tree cover	-0.491	0.006	0.162	-0.724	-0.042	*
	FD	-0.235	0.010	0.104	-0.456	-0.043	*
**MeanTotAbu [R2: 0.52]**	MeanRich	0.721	-0.002	0.080	0.517	0.844	*
**wAPV [R2: 0.24]**	FD	0.490	-0.004	0.121	0.203	0.690	*
**Stability [R2: 0.79]**	MeanRich	0.486	-0.014	0.084	0.332	0.661	*
	synchrony	-0.632	0.025	0.101	-0.822	-0.451	*
	MeanTotAbu	-0.281	0.014	0.073	-0.439	-0.155	*
	wAPV	-0.443	0.012	0.111	-0.671	-0.234	*

Table S8. Results of the SEM exploring the interplay between land use, acknowledged stability-driving mechanisms, and temporal stability for herbivores in grasslands. For each response variable within each model of the SEM, direct effects are reported as fully standardised model coefficients, and R^2 values are reported in brackets for each of the constituent models (whose name appears in bold). MeanTreeCov: mean tree cover. MeanRich: mean species richness. FD: functional diversity. MeanTotAbu: mean total abundance. wAPV: weighted average population variability. PC1 and PC2: First and second PCA axes used to define the dominant species traits.

Arthropod herbivores (grassland)

Fisher's C = 40.074 with P-value = 0.103 and on 30 degrees of freedom

Response variable	Predictor	Effect	Bias	Std. Err.	Lower CI	Upper CI	significance
**PC2 [R2: 0.03]**	LUI	-0.179	0.000	0.078	-0.325	-0.022	*
**MeanRich [R2: 0.17]**	LUI	-0.168	0.003	0.081	-0.324	-0.007	*
	PC2	0.334	-0.003	0.081	0.157	0.478	*
**FD [R2: 0.44]**	PC1	0.403	-0.001	0.052	0.299	0.505	*
	PC2	-0.528	0.006	0.060	-0.645	-0.414	*
**synchrony [R2: 0.04]**	FD	-0.192	0.002	0.082	-0.351	-0.031	*
**MeanTotAbu [R2: 0.60]**	LUI	0.111	-0.001	0.040	0.031	0.191	*
	PC1	-0.107	0.000	0.038	-0.179	-0.031	*
	MeanRich	0.548	0.000	0.063	0.419	0.662	*
	FD	-0.205	0.005	0.042	-0.290	-0.125	*
	synchrony	0.284	-0.003	0.050	0.182	0.378	*
**wAPV [R2: 0.47]**	PC1	0.229	0.003	0.050	0.121	0.316	*
	PC2	-0.137	0.005	0.063	-0.267	-0.021	*
	MeanRich	-0.423	0.003	0.057	-0.531	-0.306	*
	FD	0.178	-0.005	0.057	0.070	0.292	*
	MeanTotAbu	0.176	-0.002	0.049	0.087	0.278	*
**Stability [R2: 0.77]**	synchrony	-0.554	-0.002	0.044	-0.639	-0.468	*
	MeanTotAbu	-0.084	0.000	0.038	-0.169	-0.017	*
	wAPV	-0.494	0.001	0.054	-0.616	-0.401	*

Table S9. Results of the SEM exploring the interplay between land use, acknowledged stability-driving mechanisms, and temporal stability for herbivores in forests. For each response variable within each model of the SEM, direct effects are reported as fully standardised model coefficients, and R^2 values are reported in brackets for each of the constituent models (whose name appears in bold). MeanTreeCov: mean tree cover. MeanRich: mean species richness. FD: functional diversity. MeanTotAbu: mean total abundance. wAPV: weighted average population variability. PC1 and PC2: First and second PCA axes used to define the dominant species traits.

Arthropod herbivores (forest)

#Fisher's C = 43.983 with P-value = 0.472 and on 44 degrees of freedom

Response variable	Predictor	Effect	Bias	Std. Err.	Lower CI	Upper CI	significance
**PC2 [R2: 0.16]**	Formi	0.395	-0.001	0.127	0.129	0.632	*
**MeanRich [R2: 0.35]**	PC1	0.591	-0.025	0.176	0.200	0.854	*
**FD [R2: 0.44]**	PC1	0.663	-0.003	0.092	0.427	0.801	*
**Synchrony [R2: 0.19]**	PC1	-0.436	0.004	0.176	-0.706	0.007	
**MeanTotAbu [R2: 0.73]**	PC2	-0.707	0.016	0.086	-0.869	-0.545	*
	MeanRich	0.417	-0.029	0.129	0.176	0.675	*
	FD	-0.361	0.014	0.089	-0.579	-0.217	*
**wAPV [R2: 0.20]**	Formi	0.445	0.001	0.118	0.166	0.641	*
**Stability [R2: 0.88]**	PC1	0.347	-0.012	0.095	0.196	0.578	*
	PC2	0.528	-0.019	0.106	0.342	0.758	*
	synchrony	-0.409	0.022	0.084	-0.580	-0.260	*
	wAPV	-0.630	0.032	0.101	-0.833	-0.461	*

Table S10. Results of the SEM exploring the interplay between land use, acknowledged stability-driving mechanisms, and temporal stability for carnivores in grasslands. For each response variable within each model of the SEM, direct effects are reported as fully standardised model coefficients, and R^2 values are reported in brackets for each of the constituent models (whose name appears in bold). MeanTreeCov: mean tree cover. MeanRich: mean species richness. FD: functional diversity. MeanTotAbu: mean total abundance. wAPV: weighted average population variability. PC1 and PC2: First and second PCA axes used to define the dominant species traits.

Arthropod carnivores (grassland)

Fisher's C = 50.672 with P-value = 0.082 and on 38 degrees of freedom

Response variable	Predictor	Effect	Bias	Std. Err.	Lower CI	Upper CI	significance
**PC2 [R2: 0.04]**	LUI	-0.209	0.000	0.071	-0.337	-0.061	*
**MeanRich [R2: 0.03]**	LUI	-0.177	0.001	0.079	-0.323	-0.017	*
**FD [R2: 0.10]**	PC1	0.322	0.000	0.074	0.167	0.457	*
**synchrony [R2: 0.04]**	MeanRich	0.193	-0.001	0.073	0.052	0.335	*
**MeanTotAbu [R2: 0.64]**	PC1	-0.148	-0.001	0.060	-0.278	-0.037	*
	MeanRich	0.736	-0.001	0.048	0.635	0.825	*
	FD	-0.167	0.002	0.041	-0.250	-0.088	*
**wAPV [R2: 0.17]**	LUI	-0.200	0.002	0.082	-0.350	-0.033	*
	PC1	-0.159	0.002	0.083	-0.329	-0.007	*
	MeanRich	-0.338	0.007	0.080	-0.495	-0.186	*
	FD	0.207	0.000	0.077	0.054	0.354	*
**Stability [R2: 0.74]**	MeanRich	0.339	0.011	0.070	0.182	0.454	*
	synchrony	-0.736	-0.001	0.039	-0.804	-0.645	*
	MeanTotAbu	-0.314	-0.010	0.075	-0.441	-0.144	*
	wAPV	-0.161	0.002	0.032	-0.221	-0.095	*

Table S11. Results of the SEM exploring the interplay between land use, acknowledged stability-driving mechanisms, and temporal stability for carnivores in forests. For each response variable within each model of the SEM, direct effects are reported as fully standardised model coefficients, and R^2 values are reported in brackets for each of the constituent models (whose name appears in bold). MeanTreeCov: mean tree cover. MeanRich: mean species richness. FD: functional diversity. MeanTotAbu: mean total abundance. wAPV: weighted average population variability. PC1 and PC2: First and second PCA axes used to define the dominant species traits.

Arthropod carnivores (forest)

Fisher's C = 6.753 with P-value = 0.344 and on 6 degrees of freedom

Response variable	Predictor	Effect	Bias	Std. Err.	Lower CI	Upper CI	significance
**MeanTotAbu [R2: 0.80]**	MeanRich	0.841	-0.011	0.066	0.682	0.930	*
	Formi	0.276	-0.005	0.099	0.095	0.483	*
**Stability [R2: 0.85]**	MeanRich	0.233	-0.011	0.068	0.111	0.380	*
	synchrony	-0.715	0.033	0.090	-0.886	-0.563	*
	wAPV	-0.416	0.020	0.098	-0.649	-0.253	*
	MeanTotAbu	-0.309	0.012	0.093	-0.522	-0.147	*

S5. Results of the six Structural Equation Models (SEMs; two for arthropods, two for herbivores, two for carnivores) exploring the interplay between land use, acknowledged stability-driving mechanisms, and temporal stability in the two habitats, considering the effect of multi-trophic interactions.

Table S12. Results of the multi-trophic SEM exploring the interplay between land use, acknowledged stability-driving mechanisms, and temporal stability for total arthropods in grasslands. Results of the Fisher's C test are shown on top of the table. For each response variable within each model of the SEM, direct effects are reported as fully standardised model coefficients, and R^2 values are reported in brackets for each of the constituent models (whose name appears in bold). MeanTreeCov: mean tree cover. MeanRich: mean species richness. FD: functional diversity. MeanTotAbu: mean total abundance. wAPV: weighted average population variability. PC1 and PC2: First and second PCA axes used to define the dominant species traits.

Arthropods (grassland; including multi-trophic interactions)

Fisher's C = 54.344 with P-value = 0.745 and on 62 degrees of freedom

Response variable	Predictor	Effect	Bias	Std. Err.	Lower CI	Upper CI	significance
**PlantPC1 [R2: 0.36]**	LUI	0.599	0.000	0.045	0.502	0.679	*
**PlantFD [R2: 0.04]**	LUI	-0.196	0.001	0.074	-0.338	-0.046	*
**PC2 [R2: 0.15]**	PlantPC1	-0.383	0.000	0.059	-0.485	-0.254	*
**MeanRich [R2: 0.34]**	LUI	-0.356	0.002	0.065	-0.486	-0.230	*
	PC1	-0.452	0.004	0.055	-0.559	-0.345	*
	PlantPC1	0.228	-0.001	0.074	0.077	0.369	*
**FD [R2: 0.34]**	PC1	0.553	-0.002	0.051	0.439	0.641	*
	PlantPC1	-0.173	0.000	0.050	-0.265	-0.068	*
**synchrony [R2: 0.13]**	LUI	0.226	-0.002	0.083	0.054	0.379	*
	PC2	0.341	0.000	0.066	0.195	0.458	*
**MeanTotAbu [R2: 0.65]**	PC1	-0.208	0.001	0.042	-0.297	-0.130	*
	MeanRich	0.394	0.004	0.059	0.274	0.504	*
	FD	-0.155	0.004	0.041	-0.230	-0.072	*
	synchrony	0.340	-0.004	0.048	0.239	0.425	*
**wAPV [R2: 0.56]**	PC1	0.294	-0.001	0.051	0.193	0.392	*
	MeanRich	-0.364	0.004	0.052	-0.467	-0.262	*
	FD	0.242	0.000	0.059	0.128	0.361	*
	MeanTotAbu	0.185	-0.002	0.050	0.094	0.289	*
**Stability [R2: 0.76]**	synchrony	-0.546	-0.003	0.047	-0.634	-0.450	*
	MeanTotAbu	-0.102	-0.001	0.043	-0.201	-0.027	*
	wAPV	-0.461	0.001	0.057	-0.591	-0.363	*

Table S13. Results of the multi-trophic SEM exploring the interplay between land use, acknowledged stability-driving mechanisms, and temporal stability for total arthropods in forests. Results of the Fisher's C test are shown on top of the table. For each response variable within each model of the SEM, direct effects are reported as fully standardised model coefficients, and R^2 values are reported in brackets for each of the constituent models (whose name appears in bold). MeanTreeCov: mean tree cover. MeanRich: mean species richness. FD: functional diversity. MeanTotAbu: mean total abundance. wAPV: weighted average population variability. PC1 and PC2: First and second PCA axes used to define the dominant species traits.

Arthropods (forest; including multi-trophic interactions)

Fisher's C = 69.713 with P-value = 0.62 and on 74 degrees of freedom

Response variable	Predictor	Effect	Bias	Std. Err.	Lower CI	Upper CI	significance
**PlantFD [R2: 0.44]**	Formi	0.663	0.007	0.117	0.295	0.830	*
**PC1 [R2: 0.20]**	Formi	-0.447	0.002	0.128	-0.662	-0.155	*
**MeanRich [R2: 0.61]**	Formi	-0.378	0.046	0.142	-0.634	-0.120	*
	PlantFD	0.295	-0.027	0.187	-0.055	0.654	
	PC1	-0.740	0.035	0.102	-0.879	-0.529	*
**FD [R2: 0.55]**	PC2	0.580	-0.003	0.109	0.291	0.747	*
	PC1	-0.458	0.009	0.090	-0.619	-0.273	*
**synchrony [R2: 0.33]**	Mean tree cover	-0.491	0.006	0.162	-0.724	-0.043	*
	FD	-0.235	0.010	0.104	-0.456	-0.043	*
**MeanTotAbu [R2: 0.63]**	PlantFD	-0.236	0.029	0.135	-0.448	0.068	
	MeanRich	0.657	-0.047	0.119	0.430	0.838	*
	synchrony	0.312	-0.013	0.118	0.084	0.5450	*
**wAPV [R2: 0.55]**	Formi	0.454	-0.030	0.134	0.185	0.695	*
	Mean tree cover	0.459	-0.041	0.135	0.203	0.683	*
	PlantPC1	-0.353	0.018	0.124	-0.587	-0.113	*
	FD	0.475	-0.025	0.111	0.283	0.701	*
**Stability [R2: 0.80]**	PlantFD	-0.103	-0.001	0.091	-0.274	0.082	
	MeanRich	0.492	-0.020	0.079	0.361	0.678	*
	synchrony	-0.494	0.036	0.110	-0.758	-0.318	*
	MeanTotAbu	-0.300	0.027	0.077	-0.476	-0.177	*
	wAPV	-0.447	0.030	0.107	-0.688	-0.265	*

Table S14. Results of the multi-trophic SEM exploring the interplay between land use, acknowledged stability-driving mechanisms, and temporal stability for herbivores in grasslands. Results of the Fisher's C test are shown on top of the table. For each response variable within each model of the SEM, direct effects are reported as fully standardised model coefficients, and R^2 values are reported in brackets for each of the constituent models (whose name appears in bold). MeanTreeCov: mean tree cover. MeanRich: mean species richness. FD: functional diversity. MeanTotAbu: mean total abundance. wAPV: weighted average population variability. PC1 and PC2: First and second PCA axes used to define the dominant species traits.

Arthropod herbivores (grassland; including multi-trophic interactions)

Fisher's C = 78.18 with P-value = 0.187 and on 68 degrees of freedom

Response variable	Predictor	Effect	Bias	Std. Err.	Lower CI	Upper CI	significance
**PlantPC1 [R2: 0.36]**	LUI	0.599	0.000	0.045	0.502	0.679	*
**PlantFD [R2: 0.04]**	LUI	-0.196	0.001	0.074	-0.338	-0.046	*
**PC1 [R2: 0.09]**	PlantPC2	-0.298	-0.001	0.069	-0.425	-0.157	*
**MeanRich [R2: 0.25]**	LUI	-0.259	0.003	0.073	-0.402	-0.115	*
	PlantPC2	-0.197	0.003	0.068	-0.328	-0.064	*
	PC2	0.377	-0.006	0.080	0.211	0.524	*
	PlantPC1	0.213	-0.002	0.064	0.089	0.338	*
**FD [R2: 0.48]**	PlantPC2	0.148	0.000	0.050	0.049	0.245	*
	PC2	-0.558	0.010	0.061	-0.676	-0.444	*
	PlantPC1	-0.124	0.002	0.052	-0.227	-0.021	*
	PC1	0.403	-0.003	0.049	0.307	0.500	*
**synchrony [R2: 0.07]**	MeanRich	-0.181	0.001	0.074	-0.324	-0.035	*
	FD	-0.234	0.003	0.078	-0.383	-0.082	*
**MeanTotAbu [R2: 0.59]**	LUI	0.125	0000	0.042	0.041	0.207	*
	MeanRich	0.541	0.002	0.063	0.408	0.656	*
	FD	-0.272	0.004	0.044	-0.359	-0.186	*
	synchrony	0.283	-0.004	0.051	0.178	0.377	*
**wAPV [R2: 0.49]**	PC2	-0.172	0.005	0.063	-0.301	-0.055	*
	PlantPC1	-0.152	0.002	0.064	-0.290	-0.035	*
	PC1	0.221	0.002	0.049	0.116	0.309	*
	MeanRich	-0.422	0.006	0.057	-0.533	-0.310	*
	FD	0.155	-0.005	0.056	0.047	0.266	*
	MeanTotAbu	0.188	-0.003	0.050	0.096	0.293	*
**Stability [R2: 0.77]**	synchrony	-0.554	0.002	0.044	-0.639	-0.468	*
	MeanTotAbu	-0.084	0.000	0.038	-0.169	-0.017	*
	wAPV	-0.494	0.001	0.054	-0.616	-0.401	*

Table S15. Results of the multi-trophic SEM exploring the interplay between land use, acknowledged stability-driving mechanisms, and temporal stability for herbivores in forests. Results of the Fisher's C test are shown on top of the table. For each response variable within each model of the SEM, direct effects are reported as fully standardised model coefficients, and R^2 values are reported in brackets for each of the constituent models (whose name appears in bold). MeanTreeCov: mean tree cover. MeanRich: mean species richness. FD: functional diversity. MeanTotAbu: mean total abundance. wAPV: weighted average population variability. PC1 and PC2: First and second PCA axes used to define the dominant species traits.

Arthropod herbivores (forest; including multi-trophic interactions)

Fisher's C = 69.293 with P-value = 0.569 and on 72 degrees of freedom

Response variable	Predictor	Effect	Bias	Std. Err.	Lower CI	Upper CI	significance
**PlantFD [R2: 0.44]**	Formi	0.663	0.007	0.117	0.295	0.830	*
**PC2 [R2: 0.27]**	PlantFD	0.522	0.008	0.100	0.308	0.699	*
**MeanRich [R2: 0.35]**	PC1	0.591	-0.025	0.176	0.200	0.854	*
**FD [R2: 0.44]**	PC1	0.663	-0.003	0.092	0.427	0.801	*
**synchrony [R2: 0.35]**	PC1	-0.430	-0.003	0.126	-0.653	-0.733	*
	PlantPC2	-0.401	0.028	0.178	-0.733	-0.055	*
**MeanTotAbu [R2: 0.73]**	PC2	-0.707	0.016	0.086	-0.869	-0.545	*
	MeanRich	0.417	-0.029	0.129	0.176	0.675	*
	FD	-0.361	0.014	0.089	-0.579	-0.217	*
**wAPV [R2: 0.20]**	Formi	0.445	0.001	0.118	0.166	0.641	*
**Stability [R2: 0.88]**	PC1	0.347	-0.012	0.095	0.196	0.578	*
	PC2	0.528	-0.019	0.106	0.342	0.758	*
	synchrony	-0.409	0.022	0.084	-0.580	-0.260	*
	wAPV	-0.630	0.032	0.101	-0.833	-0.461	*

Table S16. Results of the multi-trophic SEM exploring the interplay between land use, acknowledged stability-driving mechanisms, and temporal stability for carnivores in grasslands. Results of the Fisher's C test are shown on top of the table. For each response variable within each model of the SEM, direct effects are reported as fully standardised model coefficients, and R^2 values are reported in brackets for each of the constituent models (whose name appears in bold). MeanTreeCov: mean tree cover. MeanRich: mean species richness. FD: functional diversity. MeanTotAbu: mean total abundance. wAPV: weighted average population variability. PC1 and PC2: First and second PCA axes used to define the dominant species traits.

Arthropod carnivores (grassland; including multi-trophic interactions)

Fisher's C = 141.569 with P-value = 0.447 and on 140 degrees of freedom

Response variable	Predictor	Effect	Bias	Std. Err.	Lower CI	Upper CI	significance
**PlantPC1 [R2: 0.36]**	LUI	0.600	-0.001	0.045	0.500	0.680	*
**PlantFD [R2: 0.04]**	LUI	-0.196	0.001	0.074	-0.338	-0.051	*
**HerbPC1 [R2: 0.09]**	PlantPC2	-0.297	-0.002	0.068	-0.421	-0.156	*
**HerbFD [R2: 0.47]**	PlantPC2	0.151	-0.001	0.052	0.050	0.250	*
	HerbPC2	-0.544	0.008	0.062	-0.667	-0.429	*
	PlantPC1	-0.128	0.002	0.053	-0.233	-0.023	*
	HerbPC1	0.407	-0.005	0.050	0.309	0.504	*
**PC1 [R2: 0.33]**	PlantPC2	-0.408	0.003	0.059	-0.520	-0.290	*
	PlantPC1	-0.350	0.006	0.069	-0.482	-0.213	*
	HerbFD	-0.261	0.001	0.066	-0.388	-0.127	*
**PC2 [R2: 0.11]**	LUI	-0.158	0.002	0.069	-0.286	-0.017	*
	HerbPC2	0.262	-0.001	0.081	0.089	0.406	*
**MeanRich [R2: 0.21]**	LUI	-0.305	0.003	0.075	-0.451	-0.158	*
	PlantPC2	0.302	-0.003	0.066	0.165	0.425	*
	PlantPC1	0.240	-0.004	0.069	0.105	0.376	*
	PC2	-0.174	0.002	0.076	-0.325	-0.026	*
**FD [R2: 0.10]**	PC1	0.322	0.000	0.074	0.167	0.457	*
**Synchrony [R2: 0.04]**	MeanRich	0.193	-0.001	0.073	0.052	0.335	*
**MeanTotAbu [R2: 0.64]**	PC1	-0.148	-0.001	0.060	-0.278	-0.037	*
	MeanRich	0.736	-0.001	0.048	0.635	0.825	*
	FD	-0.167	0.002	0.041	-0.250	-0.088	*
**wAPV [R2: 0.17]**	LUI	-0.200	0.002	0.082	-0.350	-0.033	*
	PC1	-0.159	0.002	0.083	-0.329	-0.007	*
	MeanRich	-0.338	0.007	0.080	-0.495	-0.186	*
	FD	0.207	0.000	0.077	0.054	0.354	*
**Stability [R2: 0.74]**	MeanRich	0.339	0.011	0.070	0.182	0.454	*
	synchrony	-0.736	-0.001	0.039	-0.804	-0.645	*
	MeanTotAbu	-0.314	-0.010	0.075	-0.441	-0.144	*
	wAPV	-0.161	0.002	0.032	-0.221	-0.095	*

Table S17. Results of the multi-trophic SEM exploring the interplay between land use, acknowledged stability-driving mechanisms, and temporal stability for carnivores in forests. Results of the Fisher's C test are shown on top of the table. For each response variable within each model of the SEM, direct effects are reported as fully standardised model coefficients, and R^2 values are reported in brackets for each of the constituent models (whose name appears in bold). MeanTreeCov: mean tree cover. MeanRich: mean species richness. FD: functional diversity. MeanTotAbu: mean total abundance. wAPV: weighted average population variability. PC1 and PC2: First and second PCA axes used to define the dominant species traits.

Arthropod carnivores (forest; including multi-trophic interactions)

Fisher's C = 107.211 with P-value = 0.246 and on 98 degrees of freedom

Response variable	Predictor	Effect	Bias	Std. Err.	Lower CI	Upper CI	significance
**PlantFD [R2: 0.44]**	Formi	0.6663	0.007	0.117	0.295	0.830	*
**HerbPC2 [R2: 0.27]**	PlantFD	0.522	0.008	0.100	0.308	0.699	*
**HerbFD [R2: 0.44]**	HerbPC1	0.663	-0.003	0.092	0.427	0.801	*
**PC2 [R2: 0.46]**	HerbPC2	-0.675	0.012	0.115	-0.828	-0.357	*
**MeanRich [R2: 0.34]**	HerbFD	0.581	-0.003	0.111	0.297	0.749	*
**Synchrony [R2: 0.32]**	PlantPC2	0.566	0.002	0.141	0.195	0.778	*
**MeanTotAbu [R2: 0.80]**	Formi	0.276	-0.005	0.099	0.095	0.483	*
	MeanRich	0.841	-0.011	0.066	0.682	0.930	*
**wAPV [R2: 0.30]**	HerbPC1	0.398	-0.002	0.111	0.167	0.605	*
	MeanTotAbu	-0.490	0.001	0.138	-0.711	-0.155	*
**Stability [R2: 0.85]**	MeanRich	0.233	-0.011	0.068	0.111	0.380	*
	synchrony	-0.715	0.033	0.090	-0.886	-0.563	*
	MeanTotAbu	-0.309	0.012	0.093	-0.522	-0.147	*
	wAPV	-0.416	0.020	0.098	-0.649	-0.253	*

S6. Structural Equation Models for total arthropods (A), herbivores (B) and carnivores (C) communities in forests, excluding (S11) and including (S12) trophic interactions.

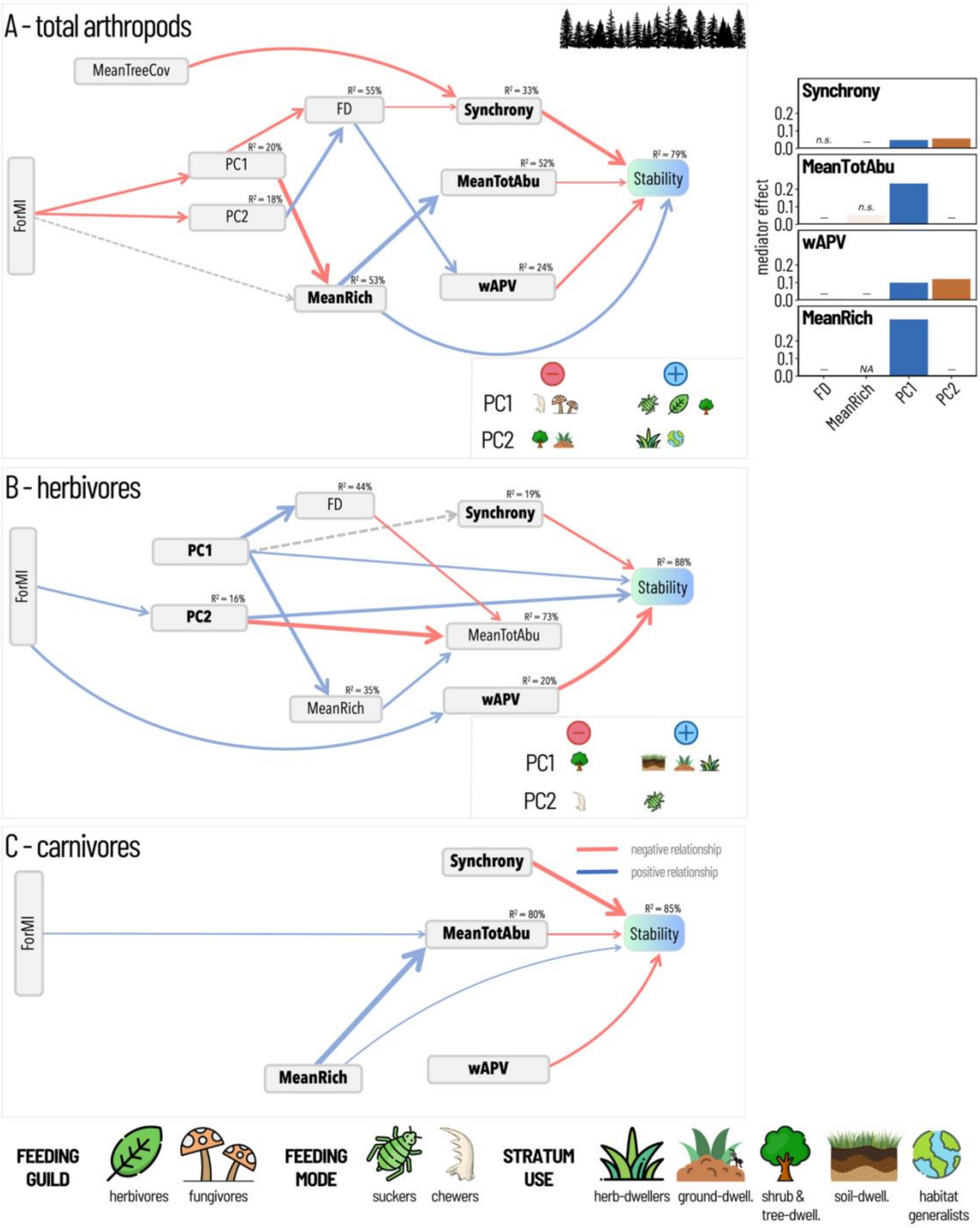

Figure S11. Structural Equation Models for total arthropods (A), herbivores (B) and carnivores (C) communities in forests, excluding trophic interactions. All SEMs showed a good fit (arthropods: Fisher's C = 60.184, df = 58, P = 0.397, n = 30; herbivores: Fisher's C = 43.983, df = 44, P = 0.472, n = 30; carnivores: Fisher's C = 6.753, df = 6, P = 0.344, n = 30). R^2 values for constituent models are reported in Table S4A. For each SEM, the effect of individual land-use intensity mediators (i.e all variables connecting land-use intensity and a direct stability driver, see Box) on direct stability drivers is shown as a barplot on the right. Long dashes replacing bars indicate that the variable on the x axis does not mediate the effect of land-use intensity on a certain direct stability driver (represented on the

y axis). NAs are for the mediating effect of a variable on itself. Bootstrapped mediator effects (i.e. the sum of all indirect paths operating through each mediator, see Box) are shown as absolute values for the purpose of comparing their strength. MeanTreeCov: mean tree cover. PC1 and PC2: First and second PCA axes used to define the dominant species traits. MeanRich: mean species richness. FD: functional diversity. MeanTotAbu: mean total abundance. wAPV: weighted average population variability. Top right drawing: @vector-trend via canva.com.

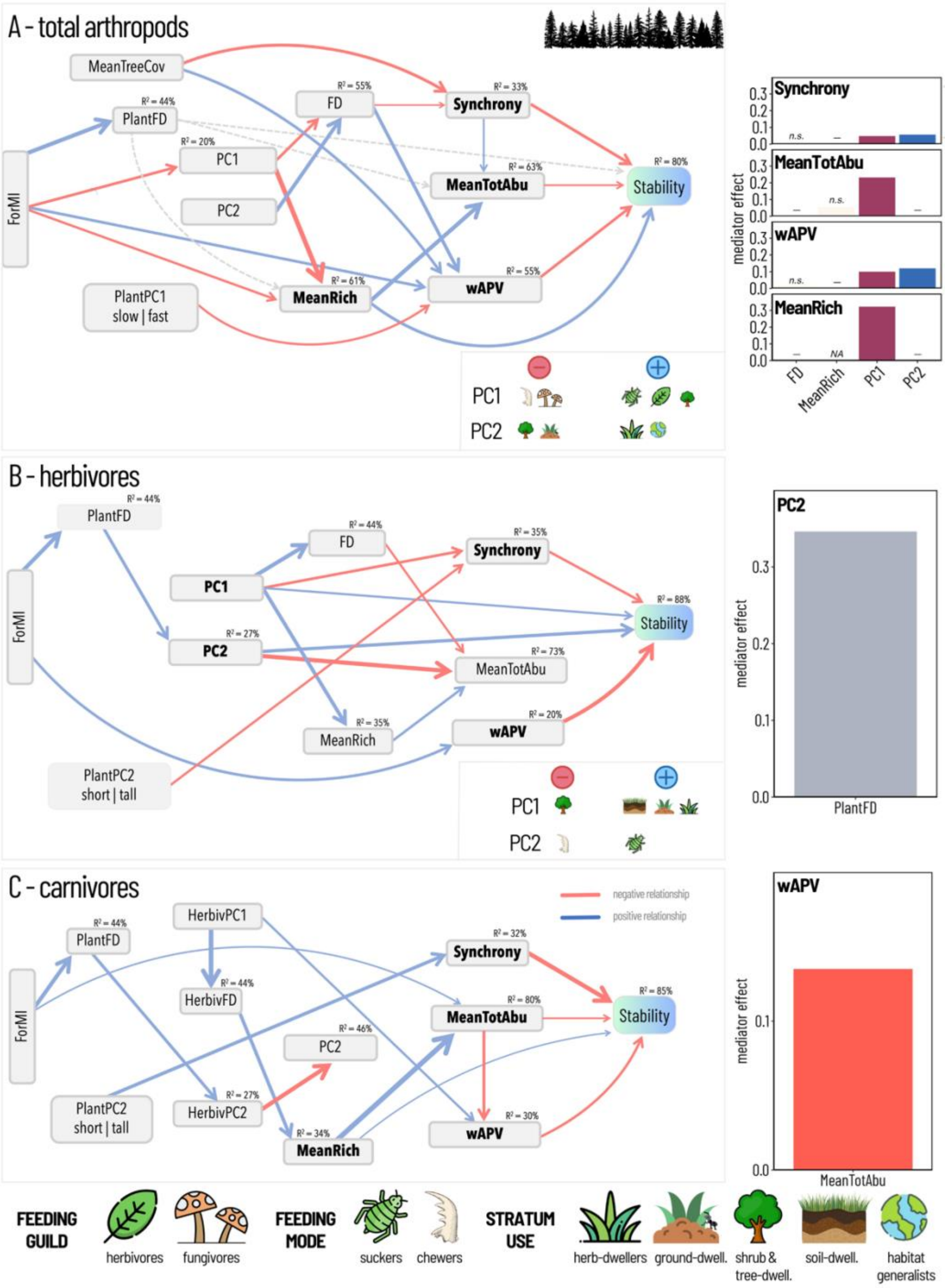

Figure S12. Structural Equation Models for total arthropods (A), herbivores (B) and carnivores (C) communities in forests, including trophic interactions. All SEMs showed a good fit (arthropods: Fisher's C = 69.713, df = 74, P = 0.62, n = 30; herbivores: Fisher's C: 69.293, df = 72, P = 0.569 , n = 30; carnivores: Fisher's C: 107.211; df = 98, P = 0.246, n = 30). R^2 values for constituent models are

reported in Table S4A. For each SEM, the effect of individual land-use intensity mediators (i.e all variables connecting land-use intensity and a direct stability driver, see Box) on direct stability drivers is shown as a barplot on the right. Long dashes replacing bars indicate that the variable on the x axis does not mediate the effect of land-use intensity on a certain direct stability driver (represented on the y axis). NAs are for the mediating effect of a variable on itself. Bootstrapped mediator effects (i.e. the sum of all indirect paths operating through each mediator, see Box) are shown as absolute values for the purpose of comparing their strength. MeanTreeCov: mean tree cover. PC1 and PC2: First and second PCA axes used to define the dominant species traits. MeanRich: mean species richness. FD: functional diversity. MeanTotAbu: mean total abundance. wAPV: weighted average population variability. Top right drawing: @vector-trend via canva.com.

S7. Mediators of the effect of land-use intensity on direct stability drivers within taxonomic groups (plants, arthropods) and across trophic levels (herbivores, carnivores) in the two habitats. For each SEM, land-use mediators (when present) are only reported for direct stability drivers. Direct stability drivers are reported for each SEM in S3A. As no land-use mediators were present for herbivores and carnivores in forests, the tables for these groups are not included. 'Mediator' effects are computed as the sum of all indirect paths operating through each individual mediator. For each stability driver, the strongest effect is highlighted in bold. MeanTreeCov: mean tree cover. MeanRich: mean species richness. FD: functional diversity. MeanTotAbu: mean total abundance. wAPV: weighted average population variability. PC1 and PC2: First and second PCA axes used to define the dominant species traits.

Table S18. Mediators of the effect of land-use intensity on direct stability drivers for plants in grasslands. Land-use mediators (when present) are only reported for direct stability drivers, which are detailed in S3A. 'Mediator' effects are computed as the sum of all indirect paths operating through each individual mediator. For each stability driver, the strongest effect is highlighted in bold. MeanTreeCov: mean tree cover. MeanRich: mean species richness. FD: functional diversity. MeanTotAbu: mean total abundance. wAPV: weighted average population variability. PC1 and PC2: First and second PCA axes used to define the dominant species traits.

Plants (grassland)

Direct stability driver	Land-use mediator	Effect	Bias	Std. Err.	Lower CI	Upper CI	significance
**MeanRich**	PC1	**-0.239**	0.002	0.029	-0.297	-0.183	*
	FD	-0.040	0.000	0.019	-0.088	-0.010	*
**wAPV**	Synchrony	0.016	0.000	0.008	0.006	0.038	*
	MeanRich	**-0.128**	0.004	0.023	-0.175	-0.085	*
	PC1	0.114	-0.002	0.043	0.038	0.210	*
	MeanTotAbu	-0.061	0.002	0.017	-0.102	-0.032	*
	FD	-0.046	0.002	0.021	-0.098	-0.013	*

Table S19. Mediators of the effect of land-use intensity on direct stability drivers for plants in forests. Land-use mediators (when present) are only reported for direct stability drivers, which are detailed in S3A. 'Mediator' effects are computed as the sum of all indirect paths operating through each individual mediator. For each stability driver, the strongest effect is highlighted in bold. MeanTreeCov: mean tree cover. MeanRich: mean species richness. FD: functional diversity. MeanTotAbu: mean total abundance. wAPV: weighted average population variability. PC1 and PC2: First and second PCA axes used to define the dominant species traits.

Plants (forest)

Direct stability driver	Land-use mediator	Effect	Bias	Std. Err.	Lower CI	Upper CI	significance
**MeanTotAbu**	MeanRich	**0.136**	-0.003	0.033	0.083	0.213	*
	FD	-0.037	0.002	0.015	-0.074	-0.014	*
**wAPV**	MeanRich	-0.045	0.001	0.013	-0.078	-0.025	*
	MeanTotAbu	-0.033	0.001	0.013	-0.064	-0.012	*
	FD	**0.121**	-0.001	0.044	0.035	0.210	*

Table S20. Mediators of the effect of land-use intensity on direct stability drivers for total arthropods in grasslands. Land-use mediators (when present) are only reported for direct stability drivers, which are detailed in S3A. 'Mediator' effects are computed as the sum of all indirect paths operating through each individual mediator. For each stability driver, the strongest effect is highlighted in bold. MeanTreeCov: mean tree cover. MeanRich: mean species richness. FD: functional diversity. MeanTotAbu: mean total abundance. wAPV: weighted average population variability. PC1 and PC2: First and second PCA axes used to define the dominant species traits.

Arthropods (grassland)

Direct stability driver	Land-use mediator	Effect	Bias	Std. Err.	Lower CI	Upper CI	significance
**synchrony**	PC2	**-0.098**	0.000	0.032	-0.165	-0.041	*
**MeanTotAbu**	synchrony	0.044	-0.001	0.029	-0.010	0.105	
	MeanRich	**-0.108**	0.000	0.032	-0.179	-0.054	*
	PC2	-0.033	0.001	0.012	-0.061	-0.014	*
**wAPV**	synchrony	0.008	0.000	0.006	-0.001	0.026	
	MeanRich	**0.080**	-0.001	0.026	0.038	0.141	*
	MeanTotAbu	-0.012	0.000	0.008	-0.033	0.001	
	PC2	-0.006	0.000	0.003	-0.015	-0.002	*

Table S21. Mediators of the effect of land-use intensity on direct stability drivers for total arthropods in forests. Land-use mediators (when present) are only reported for direct stability drivers, which are detailed in S3A. 'Mediator' effects are computed as the sum of all indirect paths operating through each individual mediator. For each stability driver, the strongest effect is highlighted in bold. MeanTreeCov: mean tree cover. MeanRich: mean species richness. FD: functional diversity. MeanTotAbu: mean total abundance. wAPV: weighted average population variability. PC1 and PC2: First and second PCA axes used to define the dominant species traits.

Arthropods (forest)

Direct stability driver	Land-use mediator	Effect	Bias	Std. Err.	Lower CI	Upper CI	significance
**synchrony**	FD	0.009	-0.002	0.034	-0.055	0.086	
	PC1	-0.048	0.002	0.029	-0.152	-0.011	*
	PC2	**0.057**	-0.004	0.035	0.009	0.161	*
**MeanRich**	PC1	**0.322**	-0.009	0.087	0.139	0.475	*
**MeanTotAbu**	MeanRich	0.054	-0.006	0.092	-0.163	0.209	
	PC1	**0.232**	-0.009	0.061	0.119	0.355	*
**wAPV**	FD	-0.019	0.005	0.065	-0.130	0.134	
	PC1	0.100	0.000	0.050	0.024	0.223	*
	PC2	**-0.120**	0.005	0.054	-0.267	-0.039	*

Table S22. Mediators of the effect of land-use intensity on direct stability drivers for herbivores in grasslands. Land-use mediators (when present) are only reported for direct stability drivers, which are detailed in S3A. 'Mediator' effects are computed as the sum of all indirect paths operating through each individual mediator. For each stability driver, the strongest effect is highlighted in bold. MeanTreeCov: mean tree cover. MeanRich: mean species richness. FD: functional diversity. MeanTotAbu: mean total abundance. wAPV: weighted average population variability. PC1 and PC2: First and second PCA axes used to define the dominant species traits.

Arthropod herbivores (grassland)

Direct stability driver	Land-use mediator	Effect	Bias	Std. Err.	Lower CI	Upper CI	significance
**synchrony**	FD	**-0.018**	0.001	0.011	-0.050	-0.003	*
	PC2	**-0.018**	0.001	0.011	-0.050	-0.003	*
**MeanTotAbu**	synchrony	-0.005	0.000	0.003	-0.016	-0.001	*
	MeanRich	**-0.125**	0.002	0.044	-0.214	-0.039	*
	FD	-0.025	0.001	0.011	-0.053	-0.006	*
	PC2	-0.057	0.001	0.027	-0.116	-0.010	*
**wAPV**	synchrony	-0.001	0.000	0.001	-0.003	0.000	
	MeanRich	**0.074**	-0.001	0.029	0.024	0.138	*
	MeanTotAbu	-0.007	0.001	0.012	-0.033	0.015	
	FD	0.012	-0.001	0.007	0.003	0.035	*
	PC2	0.057	-0.002	0.024	0.012	0.107	*

Table S23. Mediators of the effect of land-use intensity on direct stability drivers for carnivores in grasslands. Land-use mediators (when present) are only reported for direct stability drivers, which are detailed in S3A. 'Mediator' effects are computed as the sum of all indirect paths operating through each individual mediator. For each stability driver, the strongest effect is highlighted in bold. MeanTreeCov: mean tree cover. MeanRich: mean species richness. FD: functional diversity. MeanTotAbu: mean total abundance. wAPV: weighted average population variability. PC1 and PC2: First and second PCA axes used to define the dominant species traits.

Arthropod carnivores (grassland)

Direct stability driver	Land-use mediator	Effect	Bias	Std. Err.	Lower CI	Upper CI	significance
**synchrony**	MeanRich	**-0.034**	0.001	0.020	-0.085	-0.005	*
**MeanTotAbu**	MeanRich	**-0.130**	0.000	0.060	-0.248	-0.015	*
**wAPV**	MeanRich	**0.060**	-0.001	0.031	0.012	0.138	*

Table S24. Mediators of the effect of land-use intensity on direct stability drivers for total arthropods in grasslands, considering multi-trophic interactions. Land-use mediators (when present) are only reported for direct stability drivers, which are detailed in S3A. 'Mediator' effects are computed as the sum of all indirect paths operating through each individual mediator. For each stability driver, the strongest effect is highlighted in bold. MeanTreeCov: mean tree cover. MeanRich: mean species richness. FD: functional diversity. MeanTotAbu: mean total abundance. wAPV: weighted average population variability. PC1 and PC2: First and second PCA axes used to define the dominant species traits.

Arthropods (grassland; including multi-trophic interactions)

Direct stability driver	Land-use mediator	Effect	Bias	Std. Err.	Lower CI	Upper CI	significance
synchrony	PC2	**-0.078**	0.000	0.021	-0.124	-0.042	*
	PlantPC1	**-0.078**	0.000	0.021	-0.124	-0.042	*
MeanTotAbu	synchrony	0.050	-0.001	0.029	-0.003	0.110	
	MeanRich	**-0.087**	0.000	0.026	-0.146	-0.042	*
	FD	0.016	0.000	0.007	0.006	0.033	*
	PC2	-0.027	0.000	0.008	-0.046	-0.014	*
	PlantPC1	0.043	0.001	0.023	0.002	0.092	*
wAPV	synchrony	0.009	0.000	0.006	0.000	0.028	
	MeanRich	0.064	-0.001	0.021	0.030	0.115	*
	MeanTotAbu	-0.004	0.000	0.007	-0.020	0.010	
	FD	-0.022	0.000	0.009	-0.045	-0.008	*
	PC2	-0.005	0.000	0.002	-0.011	-0.002	*
	PlantPC1	**-0.067**	0.000	0.020	-0.110	-0.033	*

Table S25. Mediators of the effect of land-use intensity on direct stability drivers for total arthropods in forests, considering multi-trophic interactions. Land-use mediators (when present) are only reported for direct stability drivers, which are detailed in S3A. 'Mediator' effects are computed as the sum of all indirect paths operating through each individual mediator. For each stability driver, the strongest effect is highlighted in bold. MeanTreeCov: mean tree cover. MeanRich: mean species richness. FD: functional diversity. MeanTotAbu: mean total abundance. wAPV: weighted average population variability. PC1 and PC2: First and second PCA axes used to define the dominant species traits.

Arthropods (forest; including multi-trophic interactions)

Direct stability driver	Land-use mediator	Effect	Bias	Std. Err.	Lower CI	Upper CI	significance
synchrony	FD	**-0.048**	0.002	0.029	-0.152	-0.011	*
	PC1	**-0.048**	0.002	0.029	-0.152	-0.011	*
MeanRich	PC1	**0.331**	-0.021	0.088	0.175	0.500	*
	PlantFD	0.195	-0.033	0.109	-0.038	0.369	
MeanTotAbu	synchrony	-0.015	0.000	0.012	-0.067	-0.002	*
	MeanRich	0.098	-0.016	0.068	-0.037	0.219	
	FD	-0.015	0.000	0.012	-0.067	-0.002	*
	PC1	**0.203**	-0.030	0.061	0.115	0.366	*
	PlantFD	-0.028	-0.013	0.132	-0.335	0.185	
wAPV	FD	**0.097**	-0.006	0.041	0.032	0.199	*
	PC1	**0.097**	-0.006	0.041	0.032	0.199	*

Table S26. Mediators of the effect of land-use intensity on direct stability drivers for herbivores in grasslands, considering multi-trophic interactions. Land-use mediators (when present) are only reported for direct stability drivers, which are detailed in S3A. 'Mediator' effects are computed as the sum of all indirect paths operating through each individual mediator. For each stability driver, the strongest effect is highlighted in bold. MeanTreeCov: mean tree cover. MeanRich: mean species richness. FD: functional diversity. MeanTotAbu: mean total abundance. wAPV: weighted average population variability. PC1 and PC2: First and second PCA axes used to define the dominant species traits.

Arthropod herbivores (grassland; including multi-trophic interactions)

Direct stability driver	Land-use mediator	Effect	Bias	Std. Err.	Lower CI	Upper CI	significance
**synchrony**	MeanRich	**0.024**	0.000	0.018	0.001	0.076	*
	FD	0.017	0.000	0.010	0.004	0.045	*
	PlantPC1	-0.006	0.000	0.015	-0.038	0.024	
**MeanTotAbu**	synchrony	0.012	0.000	0.006	0.003	0.030	*
	MeanRich	-0.064	0.001	0.033	-0.136	-0.005	*
	FD	0.025	-0.001	0.012	0.006	0.053	*
	PlantPC1	**0.088**	-0.001	0.024	0.046	0.141	*
**wAPV**	synchrony	0.002	0.000	0.001	0.000	0.007	
	MeanRich	0.043	-0.001	0.024	0.004	0.100	*
	MeanTotAbu	0.016	0.000	0.012	-0.003	0.046	
	FD	-0.007	0.001	0.005	-0.022	0.000	
	PlantPC1	**-0.140**	0.002	0.044	-0.236	-0.063	*

Table S27. Mediators of the effect of land-use intensity on direct stability drivers for herbivores in forests, considering multi-trophic interactions. Land-use mediators (when present) are only reported for direct stability drivers, which are detailed in S3A. 'Mediator' effects are computed as the sum of all indirect paths operating through each individual mediator. For each stability driver, the strongest effect is highlighted in bold. MeanTreeCov: mean tree cover. MeanRich: mean species richness. FD: functional diversity. MeanTotAbu: mean total abundance. wAPV: weighted average population variability. PC1 and PC2: First and second PCA axes used to define the dominant species traits.

Arthropod herbivores (forest; including multi-trophic interactions)

Direct stability driver	Land-use mediator	Effect	Bias	Std. Err.	Lower CI	Upper CI	significance
**PC2**	PlantFD	**0.346**	0.015	0.112	0.118	0.546	*

Table S28. Mediators of the effect of land-use intensity on direct stability drivers for carnivores in grasslands, considering multi-trophic interactions. Land-use mediators (when present) are only reported for direct stability drivers, which are detailed in S3A. 'Mediator' effects are computed as the sum of all indirect paths operating through each individual mediator. For each stability driver, the strongest effect is highlighted in bold. MeanTreeCov: mean tree cover. MeanRich: mean species richness. FD: functional diversity. MeanTotAbu: mean total abundance. wAPV: weighted average population variability. PC1 and PC2: First and second PCA axes used to define the dominant species traits.

Arthropod carnivores (grassland; including multi-trophic interactions)

Direct stability driver	Land-use mediator	Effect	Bias	Std. Err.	Lower CI	Upper CI	significance
**synchrony**	MeanRich	-0.026	0.001	0.015	-0.066	-0.004	*
	PC2	0.005	0.000	0.004	0.001	0.019	*
	PlantPC1	**0.028**	-0.001	0.012	0.010	0.059	*
**MeanRich**	PC2	0.027	0.000	0.019	0.002	0.079	*
	PlantPC1	**0.144**	-0.002	0.044	0.062	0.234	*
**MeanTotAbu**	MeanRich	-0.099	0.001	0.046	-0.197	-0.015	*
	FD	0.010	0.000	0.004	0.004	0.022	*
	PC1	0.038	-0.001	0.013	0.018	0.074	*
	PC2	0.020	0.000	0.014	0.001	0.059	*
	Herbrao	-0.004	0.000	0.003	-0.013	-0.001	*
	PlantPC1	**0.144**	-0.003	0.035	0.082	0.219	*
**wAPV**	MeanRich	**0.045**	-0.001	0.024	0.009	0.110	*
	FD	-0.013	0.000	0.007	-0.032	-0.004	*
	PC1	0.018	0.000	0.017	-0.007	0.064	
	PC2	-0.009	0.000	0.006	-0.029	-0.001	*
	Herbrao	-0.002	0.000	0.002	-0.010	0.000	
	PlantPC1	-0.031	0.001	0.026	-0.089	0.014	

Table S29. Mediators of the effect of land-use intensity on direct stability drivers for carnivores in forests, considering multi-trophic interactions. Land-use mediators (when present) are only reported for direct stability drivers, which are detailed in S3A. 'Mediator' effects are computed as the sum of all indirect paths operating through each individual mediator. For each stability driver, the strongest effect is highlighted in bold. MeanTreeCov: mean tree cover. MeanRich: mean species richness. FD: functional diversity. MeanTotAbu: mean total abundance. wAPV: weighted average population variability. PC1 and PC2: First and second PCA axes used to define the dominant species traits.

Arthropod carnivores (forest; including multi-trophic interactions)

Direct stability driver	Land-use mediator	Effect	Bias	Std. Err.	Lower CI	Upper CI	significance
**wAPV**	MeanTotAbu	**-0.135**	0.002	0.064	-0.307	-0.038	*

S8. Correlation matrices showing the correlation between the first two axes of the Principal Component Analysis (PCA), functional diversity and the community weighted means (CWM) of body size and dispersal ability for total arthropods, herbivores and carnivores in grasslands and forests. Crossed cells represent Pearson correlation coefficients not significant at $p < 0.05$. FD: functional diversity.

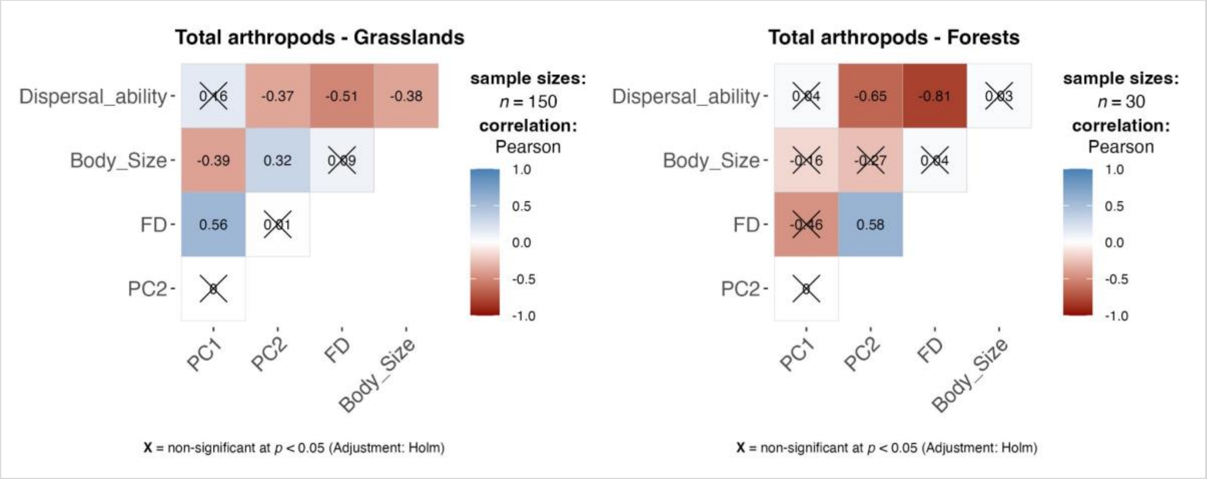

Figure S13. Correlation matrices showing the correlation between the first two PC axes, functional diversity and the CWMs of body size and dispersal ability for total arthropods in grasslands and forests. Crossed cells represent Pearson correlation coefficients not significant at $p < 0.05$. FD: functional diversity.

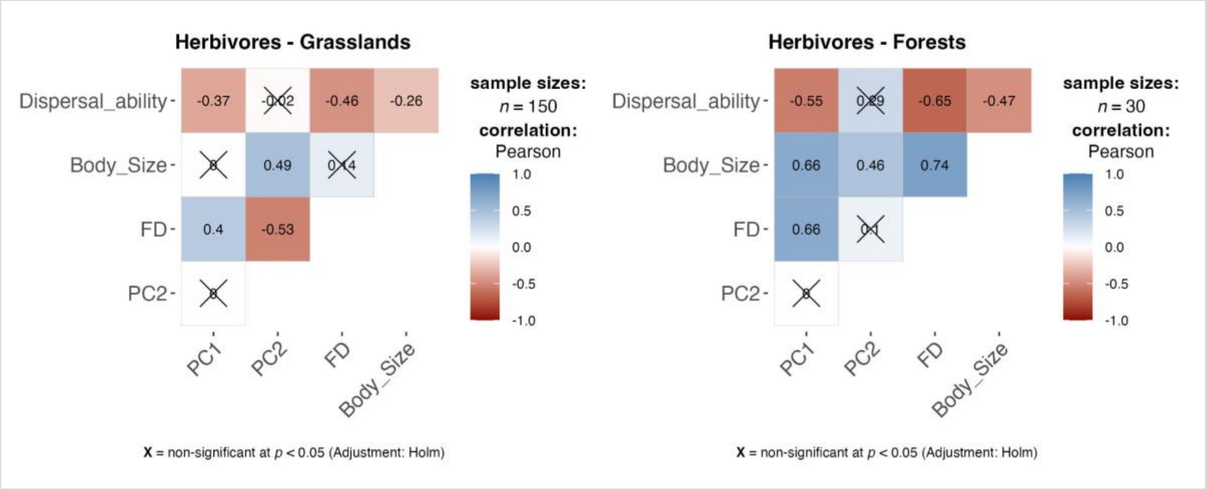

Figure S14. Correlation matrices showing the correlation between the first two PC axes, functional diversity and the CWMs of body size and dispersal ability for herbivores in grasslands and forests. Crossed cells represent Pearson correlation coefficients not significant at $p < 0.05$. FD: functional diversity.

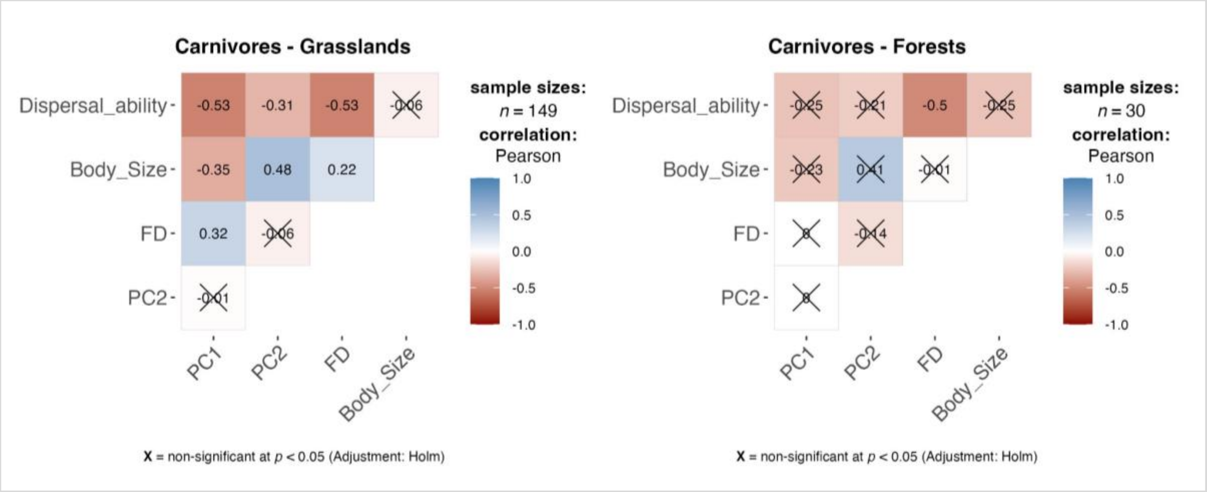

Figure S15. Correlation matrices showing the correlation between the first two PC axes, functional diversity and the CWMs of body size and dispersal ability for herbivores in grasslands and forests. Crossed cells represent Pearson correlation coefficients not significant at $p < 0.05$. FD: functional diversity.

REFERENCES AND NOTES

1. D. Tilman, P. B. Reich, J. M. H. Knops, Biodiversity and ecosystem stability in a decade-long grassland experiment. *Nature* **441**, 629–632 (2006).

2. S. Dıaz, J. Settele, E. Brondızio, H. Ngo, M. Guèze, J. Agard, A. Arneth, P. Balvanera, K. Brauman, S. Butchart, “Summary for policy makers of the global assessment report on biodiversity and ecosystem services of the Intergovernmental Science-Policy Platform on Biodiversity and Ecosystem Services” (IPBES Secretariat, Intergovernmental Science-Policy Platform on Biodiversity and Ecosystem Services, 2019).

3. G. Ceballos, P. R. Ehrlich, A. D. Barnosky, A. García, R. M. Pringle, T. M. Palmer, Accelerated modern human–induced species losses: Entering the sixth mass extinction. *Sci. Adv.* **1**, e1400253 (2015).

4. R. van Klink, D. E. Bowler, K. B. Gongalsky, M. Shen, S. R. Swengel, J. M. Chase, Disproportionate declines of formerly abundant species underlie insect loss. *Nature*, **628**, 359–364 (2024).

5. L. M. Hallett, J. S. Hsu, E. E. Cleland, S. L. Collins, T. L. Dickson, E. C. Farrer, L. A. Gherardi, K. L. Gross, R. J. Hobbs, L. Turnbull, K. N. Suding, Biotic mechanisms of community stability shift along a precipitation gradient. *Ecology* **95**, 1693–1700 (2014).

6. Y. Hautier, E. W. Seabloom, E. T. Borer, P. B. Adler, W. S. Harpole, H. Hillebrand, E. M. Lind, A. S. MacDougall, C. J. Stevens, J. D. Bakker, Y. M. Buckley, C. Chu, S. L. Collins, P. Daleo, E. I. Damschen, K. F. Davies, P. A. Fay, J. Firn, D. S. Gruner, V. L. Jin, J. A. Klein, J. M. H. Knops, K. J. La Pierre, W. Li, R. L. McCulley, B. A. Melbourne, J. L. Moore, L. R. O’Halloran, S. M. Prober, A. C. Risch, M. Sankaran, M. Schuetz, A. Hector, Eutrophication weakens stabilizing effects of diversity in natural grasslands. *Nature* **508**, 521–525 (2014).

7. D. Craven, N. Eisenhauer, W. D. Pearse, Y. Hautier, F. Isbell, C. Roscher, M. Bahn, C. Beierkuhnlein, G. Bönisch, N. Buchmann, C. Byun, J. A. Catford, B. E. L. Cerabolini, J. H. C. Cornelissen, J. M. Craine, E. De Luca, A. Ebeling, J. N. Griffin, A. Hector, J. Hines, A. Jentsch, J. Kattge, J. Kreyling, V. Lanta, N. Lemoine, S. T. Meyer, V. Minden, V. Onipchenko, H. W. Polley, P. B. Reich, J. Van Ruijven, B. Schamp, M. D. Smith, N. A. Soudzilovskaia, D. Tilman, A. Weigelt, B. Wilsey, P. Manning,

Multiple facets of biodiversity drive the diversity–stability relationship. *Nat. Ecol. Evol.* **2**, 1579–1587 (2018).

8. E. Valencia, F. De Bello, T. Galland, P. B. Adler, J. Lepš, A. E-Vojtkó, R. Van Klink, C. P. Carmona, J. Danihelka, J. Dengler, D. J. Eldridge, M. Estiarte, R. García-González, E. Garnier, D. Gómez-García, S. P. Harrison, T. Herben, R. Ibáñez, A. Jentsch, N. Juergens, M. Kertész, K. Klumpp, F. Louault, R. H. Marrs, R. Ogaya, G. Ónodi, R. J. Pakeman, I. Pardo, M. Pärtel, B. Peco, J. Peñuelas, R. F. Pywell, M. Rueda, W. Schmidt, U. Schmiedel, M. Schuetz, H. Skálová, P. Šmilauer, M. Šmilauerová, C. Smit, M. Song, M. Stock, J. Val, V. Vandvik, D. Ward, K. Wesche, S. K. Wiser, B. A. Woodcock, T. P. Young, F.-H. Yu, M. Zobel, L. Götzenberger, Synchrony matters more than species richness in plant community stability at a global scale. *Proc. Natl. Acad. Sci. U.S.A.* **117**, 24345–24351 (2020).

9. M. Loreau, C. De Mazancourt, Species synchrony and its drivers: Neutral and nonneutral community dynamics in fluctuating environments. *Am. Nat.* **172**, E48–E66 (2008).

10. J. Lepš, M. Májeková, A. Vítová, J. Doležal, F. De Bello, Stabilizing effects in temporal fluctuations: Management, traits, and species richness in high-diversity communities. *Ecology* **99**, 360–371 (2018).

11. I. Hanski, Single-species metapopulation dynamics: Concepts, models and observations. *Biol. J. Linn. Soc. Lond.* **42**, 17–38 (1991).

12. M. Loreau, C. De Mazancourt, Biodiversity and ecosystem stability: A synthesis of underlying mechanisms. *Ecol. Lett.* **16**, 106–115 (2013).

13. J. Lepš, M. Šmilauerová, P. Šmilauer, Competition among functional groups increases asynchrony of their temporal fluctuations in a temperate grassland. *J. Veg. Sci.* **30**, 1068–1077 (2019).

14. D. F. Doak, D. Bigger, E. K. Harding, M. A. Marvier, R. E. O'Malley, D. Thomson, The statistical inevitability of stability-diversity relationships in community ecology. *Am. Nat.* **151**, 264–276 (1998).

15. K. S. McCann, The diversity–stability debate. *Nature* **405**, 228–233 (2000).

16. L. M. Thibaut, S. R. Connolly, Understanding diversity–stability relationships: Towards a unified model of portfolio effects. *Ecol. Lett.* **16**, 140–150 (2013).

17. J. P. Grime, Benefits of plant diversity to ecosystems: Immediate, filter and founder effects. *J. Ecol.* **86**, 902–910 (1998).

18. M. Májeková, F. De Bello, J. Doležal, J. Lepš, Plant functional traits as determinants of population stability. *Ecology* **95**, 2369–2374 (2014).

19. E. Allan, P. Manning, F. Alt, J. Binkenstein, S. Blaser, N. Blüthgen, S. Böhm, F. Grassein, N. Hölzel, V. H. Klaus, T. Kleinebecker, E. K. Morris, Y. Oelmann, D. Prati, S. C. Renner, M. C. Rillig, M. Schaefer, M. Schloter, B. Schmitt, I. Schöning, M. Schrumpf, E. Solly, E. Sorkau, J. Steckel, I. Steffen-Dewenter, B. Stempfhuber, M. Tschapka, C. N. Weiner, W. W. Weisser, M. Werner, C. Westphal, W. Wilcke, M. Fischer, Land use intensification alters ecosystem multifunctionality via loss of biodiversity and changes to functional composition. *Ecol. Lett.* **18**, 834–843 (2015).

20. Y. Hautier, D. Tilman, F. Isbell, E. W. Seabloom, E. T. Borer, P. B. Reich, Anthropogenic environmental changes affect ecosystem stability via biodiversity. *Science* **348**, 336–340 (2015).

21. N. Blüthgen, N. K. Simons, K. Jung, D. Prati, S. C. Renner, S. Boch, M. Fischer, N. Hölzel, V. H. Klaus, T. Kleinebecker, M. Tschapka, W. W. Weisser, M. M. Gossner, Land use imperils plant and animal community stability through changes in asynchrony rather than diversity. *Nat. Commun.* **7**, 10697 (2016).

22. J. Lepš, Variability in population and community biomass in a grassland community affected by environmental productivity and diversity. *Oikos* **107**, 64–71 (2004).

23. N. M. Haddad, G. M. Crutsinger, K. Gross, J. Haarstad, J. M. H. Knops, D. Tilman, Plant species loss decreases arthropod diversity and shifts trophic structure. *Ecol. Lett.* **12**, 1029–1039 (2009).

24. N.-F. Wan, X.-R. Zheng, L.-W. Fu, L. P. Kiær, Z. Zhang, R. Chaplin-Kramer, M. Dainese, J. Tan, S.-Y. Qiu, Y.-Q. Hu, W.-D. Tian, M. Nie, R.-T. Ju, J.-Y. Deng, J.-X. Jiang, Y.-M. Cai, B. Li, Global synthesis of effects of plant species diversity on trophic groups and interactions. *Nat. Plants* **6**, 503–510 (2020).

25. C. Violle, M. Navas, D. Vile, E. Kazakou, C. Fortunel, I. Hummel, E. Garnier, Let the concept of trait be functional! *Oikos* **116**, 882–892 (2007).

26. F. De Bello, S. Lavorel, L. M. Hallett, E. Valencia, E. Garnier, C. Roscher, L. Conti, T. Galland, M. Goberna, M. Májeková, A. Montesinos-Navarro, J. G. Pausas, M. Verdú, A. E-Vojtkó, L. Götzenberger, J. Lepš, Functional trait effects on ecosystem stability: Assembling the jigsaw puzzle. *Trends Ecol. Evol.* **36**, 822–836 (2021).

27. N. K. Simons, W. W. Weisser, M. M. Gossner, Multi-taxa approach shows consistent shifts in arthropod functional traits along grassland land-use intensity gradient. *Ecology* **97**, 754–764 (2016).

28. V. Busch, V. H. Klaus, D. Schäfer, D. Prati, S. Boch, J. Müller, M. Chisté, K. Mody, N. Blüthgen, M. Fischer, N. Hölzel, T. Kleinebecker, Will I stay or will I go? Plant species-specific response and tolerance to high land-use intensity in temperate grassland ecosystems. *J. Veg. Sci.* **30**, 674–686 (2019).

29. L. M. Hallett, C. Stein, K. N. Suding, Functional diversity increases ecological stability in a grazed grassland. *Oecologia* **183**, 831–840 (2017).

30. R. Van Klink, J. Lepš, R. Vermeulen, F. De Bello, Functional differences stabilize beetle communities by weakening interspecific temporal synchrony. *Ecology* **100**, e02748 (2019).

31. I. J. Wright, P. B. Reich, M. Westoby, D. D. Ackerly, Z. Baruch, F. Bongers, J. Cavender-Bares, T. Chapin, J. H. C. Cornelissen, M. Diemer, J. Flexas, E. Garnier, P. K. Groom, J. Gulias, K. Hikosaka, B. B. Lamont, T. Lee, W. Lee, C. Lusk, J. J. Midgley, M.-L. Navas, Ü. Niinemets, J. Oleksyn, N. Osada, H. Poorter, P. Poot, L. Prior, V. I. Pyankov, C. Roumet, S. C. Thomas, M. G. Tjoelker, E. J. Veneklaas, R. Villar, The worldwide leaf economics spectrum. *Nature* **428**, 821–827 (2004).

32. L. Zheng, K. E. Barry, N. R. Guerrero-Ramírez, D. Craven, P. B. Reich, K. Verheyen, M. Scherer-Lorenzen, N. Eisenhauer, N. Barsoum, J. Bauhus, H. Bruelheide, J. Cavender-Bares, J. Dolezal, H. Auge, M. V. Fagundes, O. Ferlian, S. Fiedler, D. I. Forrester, G. Ganade, T. Gebauer, J. Haase, P. Hajek, A. Hector, B. Hérault, D. Hölscher, K. B. Hulvey, B. Irawan, H. Jactel, J. Koricheva, H. Kreft, V. Lanta, J. Leps, S. Mereu, C. Messier, F. Montagnini, M. Mörsdorf, S. Müller, B. Muys, C. A. Nock, A. Paquette, W. C. Parker, J. D. Parker, J. A. Parrotta, G. B. Paterno, M. P. Perring, D. Piotto, H. W. Polley, Q. Ponette, C. Potvin, J. Quosh, B. Rewald, D. L. Godbold, J. van Ruijven, R. J. Standish, A. Stefanski, L. Sundawati, J. Urgoiti, L. J. Williams, B. J. Wilsey, B. Yang, L. Zhang, Z. Zhao, Y. Yang, H. Sandén, A. Ebeling, B. Schmid, M. Fischer, M. M. Kotowska, C. Palmborg, D. Tilman, E. Yan, Y.

Hautier, Effects of plant diversity on productivity strengthen over time due to trait-dependent shifts in species overyielding. *Nat. Commun.* **15**, 2078 (2024).

33. M. Moretti, F. De Bello, S. Ibanez, S. Fontana, G. B. Pezzatti, F. Dziock, C. Rixen, S. Lavorel, Linking traits between plants and invertebrate herbivores to track functional effects of land-use changes. *J. Veg. Sci.* **24**, 949–962 (2013).

34. R. J. Pakeman, J. A. Stockan, Drivers of carabid functional diversity: Abiotic environment, plant functional traits, or plant functional diversity? *Ecology* **95**, 1213–1224 (2014).

35. C. N. Weiner, M. Werner, K. E. Linsenmair, N. Blüthgen, Land-use impacts on plant–pollinator networks: Interaction strength and specialization predict pollinator declines. *Ecology* **95**, 466–474 (2014).

36. S. Lavorel, J. Storkey, R. D. Bardgett, F. De Bello, M. P. Berg, X. Le Roux, M. Moretti, C. Mulder, R. J. Pakeman, S. Díaz, R. Harrington, A novel framework for linking functional diversity of plants with other trophic levels for the quantification of ecosystem services. *J. Veg. Sci.* **24**, 942–948 (2013).

37. F. Neff, N. Blüthgen, M. N. Chisté, N. K. Simons, J. Steckel, W. W. Weisser, C. Westphal, L. Pellissier, M. M. Gossner, Cross-scale effects of land use on the functional composition of herbivorous insect communities. *Landsc. Ecol.* **34**, 2001–2015 (2019).

38. H. W. Polley, F. I. Isbell, B. J. Wilsey, Plant functional traits improve diversity-based predictions of temporal stability of grassland productivity. *Oikos* **122**, 1275–1282 (2013).

39. M. Valerio, R. Ibáñez, A. Gazol, L. Götzenberger, Long-term and year-to-year stability and its drivers in a Mediterranean grassland. *J. Ecol.* **110**, 1174–1188 (2022).

40. L. Conti, E. Valencia, T. Galland, L. Götzenberger, J. Lepš, A. E-Vojtkó, C. P. Carmona, M. Májeková, J. Danihelka, J. Dengler, D. J. Eldridge, M. Estiarte, R. García-González, E. Garnier, D. Gómez, V. Hadincová, S. P. Harrison, T. Herben, R. Ibáñez, A. Jentsch, N. Juergens, M. Kertész, K. Klumpp, F. Krahulec, F. Louault, R. H. Marrs, G. Ónodi, R. J. Pakeman, M. Pärtel, B. Peco, J. Peñuelas, M. Rueda, W. Schmidt, U. Schmiedel, M. Schuetz, H. Skalova, P. Šmilauer, M. Šmilauerová, C. Smit, M. Song, M. Stock, J. Val, V. Vandvik, D. Ward, K. Wesche, S. K. Wiser, B. A. Woodcock, T. P. Young, F.-H. Yu,

M. Zobel, F. de Bello, Functional trait trade-offs define plant population stability across different biomes. *Proc. R. Soc. Lond. B Biol. Sci.* **290**, 20230344 (2023).

41. M. Fischer, O. Bossdorf, S. Gockel, F. Hänsel, A. Hemp, D. Hessenmöller, G. Korte, J. Nieschulze, S. Pfeiffer, D. Prati, S. Renner, I. Schöning, U. Schumacher, K. Wells, F. Buscot, E. K. V. Kalko, K. E. Linsenmair, E.-D. Schulze, W. W. Weisser, Implementing large-scale and long-term functional biodiversity research: The biodiversity exploratories. *Basic Appl. Ecol.* **11**, 473–485 (2010).

42. L. Zhang, W. Bai, Y. Zhang, H. Lambers, W.-H. Zhang, Ecosystem stability is determined by plant defence functional traits and population stability under mowing in a semi-arid temperate steppe. *Funct. Ecol.* **37**, 2413–2424 (2023).

43. F. Neff, D. Prati, R. Achury, D. Ambarlı, R. Bolliger, M. Brändle, M. Freitag, N. Hölzel, T. Kleinebecker, A. Knecht, D. Schäfer, P. Schall, S. Seibold, M. Staab, W. W. Weisser, L. Pellissier, M. M. Gossner, Reduction of invertebrate herbivory by land use is only partly explained by changes in plant and insect characteristics. *Ecological monographs* **93**, e1571 (2023).

44. M. M. Gossner, W. W. Weisser, S. T. Meyer, Invertebrate herbivory decreases along a gradient of increasing land-use intensity in German grasslands. *Basic Appl. Ecol.* **15**, 347–352 (2014).

45. S. T. Meyer, L. Heuss, H. Feldhaar, W. W. Weisser, M. M. Gossner, Land-use components, abundance of predatory arthropods, and vegetation height affect predation rates in grasslands. *Agric. Ecosyst. Environ.* **270-271**, 84–92 (2019).

46. E. T. Borer, E. W. Seabloom, D. Tilman, Plant diversity controls arthropod biomass and temporal stability. *Ecol. Lett.* **15**, 1457–1464 (2012).

47. Z. Li, H. Zhang, Y. Xu, S. Wang, Composition of ‘fast–slow’ traits drives avian community stability over North America. *Funct. Ecol.* **35**, 2831–2840 (2021).

48. M. Huang, S. Wang, X. Liu, M. Nie, S. Zhou, Y. Hautier, Intra- and interspecific variability of specific leaf area mitigate the reduction of community stability in response to warming and nitrogen addition. *Oikos* **2022**, e09207 (2022).

49. P. B. Reich, The world-wide 'fast–slow' plant economics spectrum: A traits manifesto. *J. Ecol.* **102**, 275–301 (2014).

50. D. Schäfer, V. H. Klaus, T. Kleinebecker, R. S. Boeddinghaus, J. Hinderling, E. Kandeler, S. Marhan, S. Nowak, I. Sonnemann, S. Wurst, M. Fischer, N. Hölzel, U. Hamer, D. Prati, Recovery of ecosystem functions after experimental disturbance in 73 grasslands differing in land-use intensity, plant species richness and community composition. *J. Ecol.* **107**, 2635–2649 (2019).

51. M. Neyret, G. L. Provost, A. L. Boesing, F. D. Schneider, D. Baulechner, J. Bergmann, F. de Vries, A. M. Fiore-Donno, S. Geisen, K. Goldmann, A. Merges, R. A. Saifutdinov, N. K. Simons, J. A. Tobias, A. S. Zaitsev, M. M. Gossner, K. Jung, E. Kandeler, J. Krauss, C. Penone, M. Schloter, S. Schulz, M. Staab, V. Wolters, A. Apostolakis, K. Birkhofer, S. Boch, R. S. Boeddinghaus, R. Bolliger, M. Bonkowski, F. Buscot, K. Dumack, M. Fischer, H. Y. Gan, J. Heinze, N. Hölzel, K. John, V. H. Klaus, T. Kleinebecker, S. Marhan, J. Müller, S. C. Renner, M. Rillig, N. V. Schenk, I. Schöning, M. Schrumpf, S. Seibold, S. Socher, E. F. Solly, M. Teuscher, M. van Kleunen, T. Wubet, P. Manning, A fast-slow trait continuum at the level of entire communities. bioRxiv 548516 [Preprint] (2023). https://doi.org/10.1101/2023.07.12.548516.

52. R. Macarthur, R. Levins, The limiting similarity, convergence, and divergence of coexisting species. *Am. Nat.* **101**, 377–385 (1967).

53. A. R. Ives, K. Gross, J. L. Klug, Stability and variability in competitive communities. *Science* **286**, 542–544 (1999).

54. F. van der Plas, T. Schröder-Georgi, A. Weigelt, K. Barry, S. Meyer, A. Alzate, R. L. Barnard, N. Buchmann, H. de Kroon, A. Ebeling, N. Eisenhauer, C. Engels, M. Fischer, G. Gleixner, A. Hildebrandt, E. Koller-France, S. Leimer, A. Milcu, L. Mommer, P. A. Niklaus, Y. Oelmann, C. Roscher, C. Scherber, M. Scherer-Lorenzen, S. Scheu, B. Schmid, E.-D. Schulze, V. Temperton, T. Tscharntke, W. Voigt, W. Weisser, W. Wilcke, C. Wirth, Plant traits alone are poor predictors of ecosystem properties and long-term ecosystem functioning. *Nat. Ecol. Evol.* **4**, 1602–1611 (2020).

55. J. L. Funk, J. E. Larson, G. M. Ames, B. J. Butterfield, J. Cavender-Bares, J. Firn, D. C. Laughlin, A. E. Sutton-Grier, L. Williams, J. Wright, Revisiting the holy grail: Using plant functional traits to understand ecological processes. *Biol. Rev.* **92**, 1156–1173 (2017).

56. S. Seibold, M. W. Cadotte, J. S. MacIvor, S. Thorn, J. Müller, The necessity of multitrophic approaches in community ecology. *Trends Ecol. Evol.* **33**, 754–764 (2018).

57. C. Frenette-Dussault, B. Shipley, Y. Hingrat, Linking plant and insect traits to understand multitrophic community structure in arid steppes. *Funct. Ecol.* **27**, 786–792 (2013).

58. F. Van Der Plas, T. M. Anderson, H. Olff, Trait similarity patterns within grass and grasshopper communities: Multitrophic community assembly at work. *Ecology* **93**, 836–846 (2012).

59. A. Ebeling, M. Rzanny, M. Lange, N. Eisenhauer, L. R. Hertzog, S. T. Meyer, W. W. Weisser, Plant diversity induces shifts in the functional structure and diversity across trophic levels. *Oikos* **127**, 208–219 (2018).

60. F. Neff, J. Hagge, R. Achury, D. Ambarlı, C. Ammer, P. Schall, S. Seibold, M. Staab, W. W. Weisser, M. M. Gossner, Hierarchical trait filtering at different spatial scales determines beetle assemblages in deadwood. *Funct. Ecol.* **36**, 2929–2942 (2022).

61. N. Blüthgen, C. F. Dormann, D. Prati, V. H. Klaus, T. Kleinebecker, N. Hölzel, F. Alt, S. Boch, S. Gockel, A. Hemp, J. Müller, J. Nieschulze, S. C. Renner, I. Schöning, U. Schumacher, S. A. Socher, K. Wells, K. Birkhofer, F. Buscot, Y. Oelmann, C. Rothenwöhrer, C. Scherber, T. Tscharntke, C. N. Weiner, M. Fischer, E. K. V. Kalko, K. E. Linsenmair, E.-D. Schulze, W. W. Weisser, A quantitative index of land-use intensity in grasslands: Integrating mowing, grazing and fertilization. *Basic Appl. Ecol.* **13**, 207–220 (2012).

62. A. Ostrowski, K. Lorenzen, E. Petzold, S. Schindler, Land use intensity index (LUI) calculation tool of the Biodiversity Exploratories project for grassland survey data from three different regions in Germany since 2006, BEXIS 2 module, version v2.0.0, Zenodo (2020); https://doi.org/10.5281/ZENODO.3865579.

63. T. Kahl, J. Bauhus, An index of forest management intensity based on assessment of harvested tree volume, tree species composition and dead wood origin. *Nat. Conserv.* **7**, 15–27 (2014).

64. M. Chytrý, Z. Otýpková, Plot sizes used for phytosociological sampling of European vegetation. *J. Veget. Sci.* **14**, 563–570 (2003).

65. T. J. Stohlgren, *Measuring Plant Diversity: Lessons From the Field* (Oxford Univ. Press, 2007).

66. S. Seibold, M. M. Gossner, N. K. Simons, N. Blüthgen, J. Müller, D. Ambarlı, C. Ammer, J. Bauhus, M. Fischer, J. C. Habel, K. E. Linsenmair, T. Nauss, C. Penone, D. Prati, P. Schall, E.-D. Schulze, J. Vogt, S. Wöllauer, W. W. Weisser, Arthropod decline in grasslands and forests is associated with landscape-level drivers. *Nature* **574**, 671–674 (2019).

67. M. M. Gossner, T. M. Lewinsohn, T. Kahl, F. Grassein, S. Boch, D. Prati, K. Birkhofer, S. C. Renner, J. Sikorski, T. Wubet, H. Arndt, V. Baumgartner, S. Blaser, N. Blüthgen, C. Börschig, F. Buscot, T. Diekötter, L. R. Jorge, K. Jung, A. C. Keyel, A.-M. Klein, S. Klemmer, J. Krauss, M. Lange, J. Müller, J. Overmann, E. Pašalić, C. Penone, D. J. Perović, O. Purschke, P. Schall, S. A. Socher, I. Sonnemann, M. Tschapka, T. Tscharntke, M. Türke, P. C. Venter, C. N. Weiner, M. Werner, V. Wolters, S. Wurst, C. Westphal, M. Fischer, W. W. Weisser, E. Allan, Land-use intensification causes multitrophic homogenization of grassland communities. *Nature* **540**, 266–269 (2016).

68. W. W. Weisser, M. Goßner, E. Pasalic, M. Lange, M. Türke, I. Gallenberger, N. Simons, M. Staab, Sweep net samples from grasslands since 2008: Araneae, Coleoptera, Hemiptera, Orthoptera, version 9, Biodiversity Exploratories Information System (2023); www.bexis.uni-jena.de/ddm/data/Showdata/21969.

69. W. W. Weisser, M. M. Gossner, E. Pasalic, M. Türke, M. Staab, Window traps on forest VIPs and EPs, 2008, 2011, 2014, 2017: Coleoptera and Hemiptera, version 6, Biodiversity Exploratories Information System (2022); www.bexis.uni-jena.de/ddm/data/Showdata/22008.

70. W. W. Weisser, M. M. Gossner, E. Pasalic, M. Türke, M. Lange, M. Staab, Window traps on forest VIPs, 2008-2017: Coleoptera and Hemiptera, version 8, Biodiversity Exploratories Information System (2021); www.bexis.uni-jena.de/ddm/data/Showdata/22007.

71. M. Neyret, G. Le Provost, A. L. Boesing, F. D. Schneider, D. Baulechner, J. Bergmann, F. T. de Vries, A. M. Fiore-Donno, S. Geisen, K. Goldmann, A. Merges, R. A. Saifutdinov, N. K. Simons, J. A. Tobias, A. S. Zaitsev, M. M. Gossner, K. Jung, E. Kandeler, J. Krauss, C. Penone, M. Schloter, S. Schulz, M. Staab, V. Wolters, A. Apostolakis, K. Birkhofer, S. Boch, R. S. Boeddinghaus, R. Bolliger, M. Bonkowski, F. Buscot, K. Dumack, M. Fischer, H. Y. Gan, J. Heinze, N. Hölzel, K. John, V. H. Klaus, T. Kleinebecker, S. Marhan, J. Müller, S. C. Renner, M. C. Rillig, N. V. Schenk, I. Schöning, M. Schrumpf, S. Seibold, S. A. Socher, E. F. Solly, M. Teuscher, M. van Kleunen, T. Wubet, P. Manning, A slow-fast trait continuum at the whole community level in relation to land-use intensification. *Nat. Commun.* **15**, 1251 (2024).

72. X. Lu, X. Zhao, T. Tachibana, K. Uchida, T. Sasaki, Y. Bai, Plant quantity and quality regulate the diversity of arthropod communities in a semi-arid grassland. *Funct. Ecol.* **35**, 601–613 (2021).

73. C. R. Rao, Diversity and dissimilarity coefficients: A unified approach. *Theor. Popul. Biol.* **21**, 24–43 (1982).

74. F. de Bello, Z. Botta-Dukát, J. Lepš, P. Fibich, Towards a more balanced combination of multiple traits when computing functional differences between species. *Methods Ecol. Evol.* **12**, 443–448 (2021).

75. J. Oksanen, G. L. Simpson, F. G. Blanchet, R. Kindt, P. Legendre, P. R. Minchin, R. B. O'Hara, P. Solymos, M. H. H. Stevens, E. Szoecs, H. Wagner, M. Barbour, M. Bedward, B. Bolker, D. Borcard, G. Carvalho, M. Chirico, M. D. Caceres, S. Durand, H. B. A. Evangelista, R. FitzJohn, M. Friendly, B. Furneaux, G. Hannigan, M. O. Hill, L. Lahti, D. McGlinn, M.-H. Ouellette, E. R. Cunha, T. Smith, A. Stier, C. J. F. T. Braak, J. Weedon, vegan: Community Ecology Package, version 2.6-4, CRAN (2022); https://cran.r-project.org/web/packages/vegan/index.html.

76. M. Májeková, T. Paal, N. S. Plowman, M. Bryndová, L. Kasari, A. Norberg, M. Weiss, T. R. Bishop, S. H. Luke, K. Sam, Y. Le Bagousse-Pinguet, J. Lepš, L. Götzenberger, F. De Bello, Evaluating functional diversity: Missing trait data and the importance of species abundance structure and data transformation. *PLOS ONE* **11**, e0149270 (2016).

77. J. S. Lefcheck, PIECEWISESEM: Piecewise structural equation modelling in R for ecology, evolution, and systematics. *Methods Ecol. Evol.* **7**, 573–579 (2016).

78. B. Shipley, Confirmatory path analysis in a generalized multilevel context. *Ecology* **90**, 363–368 (2009).

79. C. F. Dormann, M. Bagnara, S. Boch, J. Hinderling, A. Janeiro-Otero, D. Schäfer, P. Schall, F. Hartig, Plant species richness increases with light availability, but not variability, in temperate forests understorey. *BMC Ecol.* **20**, 43 (2020).

80. K. Bartoń, MuMIn: Multi-Model Inference, version 1.47.5, CRAN (2023); https://cran.r-project.org/web/packages/MuMIn/index.html.

81. K. P. Burnham, D. R. Anderson, Eds., *Model Selection and Multimodel Inference* (Springer, 2004); http://link.springer.com/10.1007/b97636.

82. F. Massol, P. David, D. Gerdeaux, P. Jarne, The influence of trophic status and large-scale climatic change on the structure of fish communities in Perialpine lakes. *J. Anim. Ecol.* **76**, 538–551 (2007).

83. M. V. Murphy, semEff: Automatic Calculation of Effects for Piecewise Structural Equation Models, version 0.6.1, CRAN (2022); https://cran.r-project.org/web/packages/semEff/index.html.

84. D. Lüdecke, M. Ben-Shachar, I. Patil, P. Waggoner, D. Makowski, performance: An R package for assessment, comparison and testing of statistical models. *J. Open Source. Softw.* **6**, 3139 (2021).

85. P. Schall, E.-D. Schulze, M. Fischer, M. Ayasse, C. Ammer, Relations between forest management, stand structure and productivity across different types of Central European forests. *Basic Appl. Ecol.* **32**, 39–52 (2018).

86. J. Vogt, V. Klaus, S. Both, C. Fürstenau, S. Gockel, M. Gossner, J. Heinze, A. Hemp, N. Hölzel, K. Jung, T. Kleinebecker, R. Lauterbach, K. Lorenzen, A. Ostrowski, N. Otto, D. Prati, S. Renner, U. Schumacher, S. Seibold, N. K. Simons, I. Steitz, M. Teuscher, J. Thiele, S. Weithmann, K. Wells, K. Wiesner, M. Ayasse, N. Blüthgen, M. Fischer, W. Weisser, Eleven years' data of grassland management in Germany. *Biodivers. Data J.* **7**, e36387 (2019).

87. R. Wisskirchen, H. Häupler, *Standardliste der Farn- und Blütenpflanzen Deutschlands* (Verlag Eugen Ulmer, 1998).

88. S. A. Socher, D. Prati, S. Boch, J. Müller, V. H. Klaus, N. Hölzel, M. Fischer, Direct and productivity-mediated indirect effects of fertilization, mowing and grazing on grassland species richness. *J. Ecol.* **100**, 1391–1399 (2012).

89. S. Boch, D. Prati, J. Müller, S. Socher, H. Baumbach, F. Buscot, S. Gockel, A. Hemp, D. Hessenmöller, E. K. V. Kalko, K. E. Linsenmair, S. Pfeiffer, U. Pommer, I. Schöning, E.-D. Schulze, C. Seilwinder, W. W. Weisser, K. Wells, M. Fischer, High plant species richness indicates management-related disturbances rather than the conservation status of forests. *Basic Appl. Ecol.* **14**, 496–505 (2013).

90. S. A. Socher, D. Prati, S. Boch, J. Müller, H. Baumbach, S. Gockel, A. Hemp, I. Schöning, K. Wells, F. Buscot, E. K. V. Kalko, K. E. Linsenmair, E.-D. Schulze, W. W. Weisser, M. Fischer, Interacting effects of fertilization, mowing and grazing on plant species diversity of 1500 grasslands in Germany differ between regions. *Basic Appl. Ecol.* **14**, 126–136 (2013).

91. T. Kleinebecker, V. Busch, N. Hölzel, U. Hamer, D. Schäfer, D. Prati, M. Fischer, A. Hemp, R. Lauterbach, V. H. Klaus, And the winner is …. ! A test of simple predictors of plant species richness in agricultural grasslands. *Ecol. Indic.* **87**, 296–301 (2018).

92. I. Hahn, I. Scheuring, The effect of measurement scales on estimating vegetation cover: A computer-assisted experiment. *Commun. Ecol.* **4**, 29–33 (2003).

93. J. Dengler, I. Dembicz, Should we estimate plant cover in percent or on ordinal scales? *Veget. Classif. Surv.* **4**, 131–138 (2023).

94. E. Breitschwerdt, H. Bruelheide, U. Jandt, Leaf traits self measured (BERICH, 2011), version 2, Biodiversity Exploratories Information System (2015); www.bexis.uni-jena.de/ddm/data/Showdata/17535?version=2.

95. D. Prati, M. Gössner, F. Neff, Leaf traits of most abundant plant species from all EPs, 2017/2018, version 2, Biodiversity Exploratories Information System (2019); www.bexis.uni-jena.de/ddm/data/Showdata/24807?version=2.

96. J. Kattge, G. Bönisch, S. Díaz, S. Lavorel, I. C. Prentice, P. Leadley, S. Tautenhahn, G. D. A. Werner, T. Aakala, M. Abedi, A. T. R. Acosta, G. C. Adamidis, K. Adamson, M. Aiba, C. H. Albert, J. M.

Alcántara, C. C. Alcázar, I. Aleixo, H. Ali, B. Amiaud, C. Ammer, M. M. Amoroso, M. Anand, C. Anderson, N. Anten, J. Antos, D. M. G. Apgaua, T. Ashman, D. H. Asmara, G. P. Asner, M. Aspinwall, O. Atkin, I. Aubin, L. Baastrup-Spohr, K. Bahalkeh, M. Bahn, T. Baker, W. J. Baker, J. P. Bakker, D. Baldocchi, J. Baltzer, A. Banerjee, A. Baranger, J. Barlow, D. R. Barneche, Z. Baruch, D. Bastianelli, J. Battles, W. Bauerle, M. Bauters, E. Bazzato, M. Beckmann, H. Beeckman, C. Beierkuhnlein, R. Bekker, G. Belfry, M. Belluau, M. Beloiu, R. Benavides, L. Benomar, M. L. Berdugo-Lattke, E. Berenguer, R. Bergamin, J. Bergmann, M. B. Carlucci, L. Berner, M. Bernhardt-Römermann, C. Bigler, A. D. Bjorkman, C. Blackman, C. Blanco, B. Blonder, D. Blumenthal, K. T. Bocanegra-González, P. Boeckx, S. Bohlman, K. Böhning-Gaese, L. Boisvert-Marsh, W. Bond, B. Bond-Lamberty, A. Boom, C. C. F. Boonman, K. Bordin, E. H. Boughton, V. Boukili, D. M. J. S. Bowman, S. Bravo, M. R. Brendel, M. R. Broadley, K. A. Brown, H. Bruelheide, F. Brumnich, H. H. Bruun, D. Bruy, S. W. Buchanan, S. F. Bucher, N. Buchmann, R. Buitenwerf, D. E. Bunker, J. Bürger, S. Burrascano, D. F. R. P. Burslem, B. J. Butterfield, C. Byun, M. Marques, M. C. Scalon, M. Caccianiga, M. Cadotte, M. Cailleret, J. Camac, J. J. Camarero, C. Campany, G. Campetella, J. A. Campos, L. Cano-Arboleda, R. Canullo, M. Carbognani, F. Carvalho, F. Casanoves, B. Castagneyrol, J. A. Catford, J. Cavender-Bares, B. E. L. Cerabolini, M. Cervellini, E. Chacón-Madrigal, K. Chapin, F. S. Chapin, S. Chelli, S. Chen, A. Chen, P. Cherubini, F. Chianucci, B. Choat, K. Chung, M. Chytrý, D. Ciccarelli, L. Coll, C. G. Collins, L. Conti, D. Coomes, J. H. C. Cornelissen, W. K. Cornwell, P. Corona, M. Coyea, J. Craine, D. Craven, J. P. G. M. Cromsigt, A. Csecserits, K. Cufar, M. Cuntz, A. C. Da Silva, K. M. Dahlin, M. Dainese, I. Dalke, M. Dalle Fratte, A. T. Dang-Le, J. Danihelka, M. Dannoura, S. Dawson, A. J. De Beer, A. De Frutos, J. R. De Long, B. Dechant, S. Delagrange, N. Delpierre, G. Derroire, A. S. Dias, M. H. Diaz-Toribio, P. G. Dimitrakopoulos, M. Dobrowolski, D. Doktor, P. Dřevojan, N. Dong, J. Dransfield, S. Dressler, L. Duarte, E. Ducouret, S. Dullinger, W. Durka, R. Duursma, O. Dymova, A. E-Vojtkó, R. L. Eckstein, H. Ejtehadi, J. Elser, T. Emilio, K. Engemann, M. B. Erfanian, A. Erfmeier, A. Esquivel-Muelbert, G. Esser, M. Estiarte, T. F. Domingues, W. F. Fagan, J. Fagúndez, D. S. Falster, Y. Fan, J. Fang, E. Farris, F. Fazlioglu, Y. Feng, F. Fernandez-Mendez, C. Ferrara, J. Ferreira, A. Fidelis, B. Finegan, J. Firn, T. J. Flowers, D. F. B. Flynn, V. Fontana, E. Forey, C. Forgiarini, L. François, M. Frangipani, D. Frank, C. Frenette-Dussault, G. T. Freschet, E. L. Fry, N. M. Fyllas, G. G. Mazzochini, S. Gachet, R. Gallagher, G. Ganade, F. Ganga, P. García-Palacios, V. Gargaglione, E. Garnier, J. L. Garrido, A. L. De Gasper, G. Gea-Izquierdo, D. Gibson, A. N. Gillison, A. Giroldo, M. Glasenhardt, S. Gleason, M. Gliesch, E. Goldberg, B. Göldel, E. Gonzalez-Akre, J. L. Gonzalez-Andujar, A. González-Melo, A. González-

Robles, B. J. Graae, E. Granda, S. Graves, W. A. Green, T. Gregor, N. Gross, G. R. Guerin, A. Günther, A. G. Gutiérrez, L. Haddock, A. Haines, J. Hall, A. Hambuckers, W. Han, S. P. Harrison, W. Hattingh, J. E. Hawes, T. He, P. He, J. M. Heberling, A. Helm, S. Hempel, J. Hentschel, B. Hérault, A. Hereş, K. Herz, M. Heuertz, T. Hickler, P. Hietz, P. Higuchi, A. L. Hipp, A. Hirons, M. Hock, J. A. Hogan, K. Holl, O. Honnay, D. Hornstein, E. Hou, N. Hough-Snee, K. A. Hovstad, T. Ichie, B. Igić, E. Illa, M. Isaac, M. Ishihara, L. Ivanov, L. Ivanova, C. M. Iversen, J. Izquierdo, R. B. Jackson, B. Jackson, H. Jactel, A. M. Jagodzinski, U. Jandt, S. Jansen, T. Jenkins, A. Jentsch, J. R. P. Jespersen, G. Jiang, J. L. Johansen, D. Johnson, E. J. Jokela, C. A. Joly, G. J. Jordan, G. S. Joseph, D. Junaedi, R. R. Junker, E. Justes, R. Kabzems, J. Kane, Z. Kaplan, T. Kattenborn, L. Kavelenova, E. Kearsley, A. Kempel, T. Kenzo, A. Kerkhoff, M. I. Khalil, N. L. Kinlock, W. D. Kissling, K. Kitajima, T. Kitzberger, R. Kjøller, T. Klein, M. Kleyer, J. Klimešová, J. Klipel, B. Kloeppel, S. Klotz, J. M. H. Knops, T. Kohyama, F. Koike, J. Kollmann, B. Komac, K. Komatsu, C. König, N. J. B. Kraft, K. Kramer, H. Kreft, I. Kühn, D. Kumarathunge, J. Kuppler, H. Kurokawa, Y. Kurosawa, S. Kuyah, J. Laclau, B. Lafleur, E. Lallai, E. Lamb, A. Lamprecht, D. J. Larkin, D. Laughlin, Y. Le Bagousse-Pinguet, G. Le Maire, P. C. Le Roux, E. Le Roux, T. Lee, F. Lens, S. L. Lewis, B. Lhotsky, Y. Li, X. Li, J. W. Lichstein, M. Liebergesell, J. Y. Lim, Y. Lin, J. C. Linares, C. Liu, D. Liu, U. Liu, S. Livingstone, J. Llusià, M. Lohbeck, Á. López-García, G. Lopez-Gonzalez, Z. Lososová, F. Louault, B. A. Lukács, P. Lukeš, Y. Luo, M. Lussu, S. Ma, C. M. R. Pereira, M. Mack, V. Maire, A. Mäkelä, H. Mäkinen, A. C. M. Malhado, A. Mallik, P. Manning, S. Manzoni, Z. Marchetti, L. Marchino, V. Marcilio-Silva, E. Marcon, M. Marignani, L. Markesteijn, A. Martin, C. Martínez-Garza, J. Martínez-Vilalta, T. Mašková, K. Mason, N. Mason, T. J. Massad, J. Masse, I. Mayrose, J. McCarthy, M. L. McCormack, K. McCulloh, I. R. McFadden, B. J. McGill, M. Y. McPartland, J. S. Medeiros, B. Medlyn, P. Meerts, Z. Mehrabi, P. Meir, F. P. L. Melo, M. Mencuccini, C. Meredieu, J. Messier, I. Mészáros, J. Metsaranta, S. T. Michaletz, C. Michelaki, S. Migalina, R. Milla, J. E. D. Miller, V. Minden, R. Ming, K. Mokany, A. T. Moles, A. Molnár, J. Molofsky, M. Molz, R. A. Montgomery, A. Monty, L. Moravcová, A. Moreno-Martínez, M. Moretti, A. S. Mori, S. Mori, D. Morris, J. Morrison, L. Mucina, S. Mueller, C. D. Muir, S. C. Müller, F. Munoz, I. H. Myers-Smith, R. W. Myster, M. Nagano, S. Naidu, A. Narayanan, B. Natesan, L. Negoita, A. S. Nelson, E. L. Neuschulz, J. Ni, G. Niedrist, J. Nieto, Ü. Niinemets, R. Nolan, H. Nottebrock, Y. Nouvellon, A. Novakovskiy, The Nutrient Network, K. O. Nystuen, A. O'Grady, K. O'Hara, A. O'Reilly-Nugent, S. Oakley, W. Oberhuber, T. Ohtsuka, R. Oliveira, K. Öllerer, M. E. Olson, V. Onipchenko, Y. Onoda, R. E. Onstein, J. C. Ordonez, N. Osada, I. Ostonen, G. Ottaviani, S. Otto, G. E.

Overbeck, W. A. Ozinga, A. T. Pahl, C. E. T. Paine, R. J. Pakeman, A. C. Papageorgiou, E. Parfionova, M. Pärtel, M. Patacca, S. Paula, J. Paule, H. Pauli, J. G. Pausas, B. Peco, J. Penuelas, A. Perea, P. L. Peri, A. C. Petisco-Souza, A. Petraglia, A. M. Petritan, O. L. Phillips, S. Pierce, V. D. Pillar, J. Pisek, A. Pomogaybin, H. Poorter, A. Portsmuth, P. Poschlod, C. Potvin, D. Pounds, A. S. Powell, S. A. Power, A. Prinzing, G. Puglielli, P. Pyšek, V. Raevel, A. Rammig, J. Ransijn, C. A. Ray, P. B. Reich, M. Reichstein, D. E. B. Reid, M. Réjou-Méchain, V. R. De Dios, S. Ribeiro, S. Richardson, K. Riibak, M. C. Rillig, F. Riviera, E. M. R. Robert, S. Roberts, B. Robroek, A. Roddy, A. V. Rodrigues, A. Rogers, E. Rollinson, V. Rolo, C. Römermann, D. Ronzhina, C. Roscher, J. A. Rosell, M. F. Rosenfield, C. Rossi, D. B. Roy, S. Royer-Tardif, N. Rüger, R. Ruiz-Peinado, S. B. Rumpf, G. M. Rusch, M. Ryo, L. Sack, A. Saldaña, B. Salgado-Negret, R. Salguero-Gomez, I. Santa-Regina, A. C. Santacruz-García, J. Santos, J. Sardans, B. Schamp, M. Scherer-Lorenzen, M. Schleuning, B. Schmid, M. Schmidt, S. Schmitt, J. V. Schneider, S. D. Schowanek, J. Schrader, F. Schrodt, B. Schuldt, F. Schurr, G. Selaya Garvizu, M. Semchenko, C. Seymour, J. C. Sfair, J. M. Sharpe, C. S. Sheppard, S. Sheremetiev, S. Shiodera, B. Shipley, T. A. Shovon, A. Siebenkäs, C. Sierra, V. Silva, M. Silva, T. Sitzia, H. Sjöman, M. Slot, N. G. Smith, D. Sodhi, P. Soltis, D. Soltis, B. Somers, G. Sonnier, M. V. Sørensen, E. E. Sosinski, N. A. Soudzilovskaia, A. F. Souza, M. Spasojevic, M. G. Sperandii, A. B. Stan, J. Stegen, K. Steinbauer, J. G. Stephan, F. Sterck, D. B. Stojanovic, T. Strydom, M. L. Suarez, J. Svenning, I. Svitková, M. Svitok, M. Svoboda, E. Swaine, N. Swenson, M. Tabarelli, K. Takagi, U. Tappeiner, R. Tarifa, S. Tauugourdeau, C. Tavsanoglu, M. Te Beest, L. Tedersoo, N. Thiffault, D. Thom, E. Thomas, K. Thompson, P. E. Thornton, W. Thuiller, L. Tichý, D. Tissue, M. G. Tjoelker, D. Y. P. Tng, J. Tobias, P. Török, T. Tarin, J. M. Torres-Ruiz, B. Tóthmérész, M. Treurnicht, V. Trivellone, F. Trolliet, V. Trotsiuk, J. L. Tsakalos, I. Tsiripidis, N. Tysklind, T. Umehara, V. Usoltsev, M. Vadeboncoeur, J. Vaezi, F. Valladares, J. Vamosi, P. M. Van Bodegom, M. Van Breugel, E. Van Cleemput, M. Van De Weg, S. Van Der Merwe, F. Van Der Plas, M. T. Van Der Sande, M. Van Kleunen, K. Van Meerbeek, M. Vanderwel, K. A. Vanselow, A. Vårhammar, L. Varone, M. Y. Vasquez Valderrama, K. Vassilev, M. Vellend, E. J. Veneklaas, H. Verbeeck, K. Verheyen, A. Vibrans, I. Vieira, J. Villacís, C. Violle, P. Vivek, K. Wagner, M. Waldram, A. Waldron, A. P. Walker, M. Waller, G. Walther, H. Wang, F. Wang, W. Wang, H. Watkins, J. Watkins, U. Weber, J. T. Weedon, L. Wei, P. Weigelt, E. Weiher, A. W. Wells, C. Wellstein, E. Wenk, M. Westoby, A. Westwood, P. J. White, M. Whitten, M. Williams, D. E. Winkler, K. Winter, C. Womack, I. J. Wright, S. J. Wright, J. Wright, B. X. Pinho, F. Ximenes, T. Yamada, K. Yamaji, R. Yanai, N. Yankov, B. Yguel, K. J. Zanini, A. E. Zanne, D. Zelený, Y. Zhao, J. Zheng, J.

Zheng, K. Ziemińska, C. R. Zirbel, G. Zizka, I. C. Zo-Bi, G. Zotz, C. Wirth, TRY plant trait database – Enhanced coverage and open access. *Glob. Chang. Biol.* **26**, 119–188 (2020).

97. M. Neyret, P. Manning, Trait data for all plant species, cleaned and aggregated from TRY, version 5, Biodiversity Exploratories Information System (2023); www.bexis.uni-jena.de/ddm/data/Showdata/27610?version=5.

98. M. M. Gossner, T. Lachat, J. Brunet, G. Isacsson, C. Bouget, H. Brustel, R. Brandl, W. W. Weisser, J. Müller, Current near-to-nature forest management effects on functional trait composition of saproxylic beetles in beech forests. *Conserv. Biol.* **27**, 605–614 (2013).

99. M. M. Gossner, N. K. Simons, R. Achtziger, T. Blick, W. H. O. Dorow, F. Dziock, F. Köhler, W. Rabitsch, W. W. Weisser, A summary of eight traits of Coleoptera, Hemiptera, Orthoptera and Araneae, occurring in grasslands in Germany. *Sci. Data* **2**, 150013 (2015).

100. S. Seibold, R. Brandl, J. Buse, T. Hothorn, J. Schmidl, S. Thorn, J. Müller, Association of extinction risk of saproxylic beetles with ecological degradation of forests in Europe. *Conserv. Biol.* **29**, 382–390 (2015).

101. M. Staab, N. Simons, M. Gössner, Body size and life-history traits of arthropod species, version 28, Biodiversity Exploratories Information System (2024); www.bexis.uni-jena.de/ddm/data/Showdata/31122?version=28.

102. S. Díaz, S. Lavorel, F. De Bello, F. Quétier, K. Grigulis, T. M. Robson, Incorporating plant functional diversity effects in ecosystem service assessments. *Proc. Natl. Acad. Sci. U.S.A.* **104**, 20684–20689 (2007).

103. A. T. C. Dias, M. P. Berg, F. de Bello, A. R. Van Oosten, K. Bílá, M. Moretti, An experimental framework to identify community functional components driving ecosystem processes and services delivery. *J. Ecol.* **101**, 29–37 (2013).

104. P. Muukkonen, R. Mäkipää, R. Laiho, K. Minkkinen, H. Vasander, L. Finér, Relationship between biomass and percentage cover in understorey vegetation of boreal coniferous forests. *Silva Fenn.* **40**, 231–245 2006).

105. I. Axmanová, L. Tichý, Z. Fajmonová, P. Hájková, E. Hettenbergerová, C.-F. Li, K. Merunková, M. Nejezchlebová, Z. Otýpková, M. Vymazalová, D. Zelený, Estimation of herbaceous biomass from species composition and cover. *Appl. Veget. Sci.* **15**, 580–589 (2012).

106. Y. Jiang, Y. Zhang, Y. Wu, R. Hu, J. Zhu, J. Tao, T. Zhang, Relationships between aboveground biomass and plant cover at two spatial scales and their determinants in northern Tibetan grasslands. *Ecol. Evol.* **7**, 7954–7964 (2017).

107. M. Fayiah, S. Dong, Y. Li, Y. Xu, X. Gao, S. Li, H. Shen, J. Xiao, Y. Yang, K. Wessell, The relationships between plant diversity, plant cover, plant biomass and soil fertility vary with grassland type on Qinghai-Tibetan Plateau. *Agric. Ecosyst. Environ.* **286**, 106659 (2019).

108. S. Lavorel, K. Grigulis, S. McIntyre, N. S. G. Williams, D. Garden, J. Dorrough, S. Berman, F. Quétier, A. Thébault, A. Bonis, Assessing functional diversity in the field – Methodology matters! *Funct. Ecol.* **22**, 134–147 (2008).
